# Zinc-metallochaperones of *Aspergillus fumigatus* are involved in ROS production and folate biosynthesis during zinc deficiency

**DOI:** 10.1128/spectrum.02279-25

**Published:** 2025-09-23

**Authors:** Clara Inés Sánchez, Verónica Díaz, Laura Alcázar, Jorge Amich, Laura Marín, José Antonio Calera

**Affiliations:** 1 Instituto de Biología Funcional y Genómica (IBFG-CSIC), Universidad de Salamanca 16779, Salamanca, Spain; 2 Laboratorio de Referencia e Investigación en Micología, Centro Nacional de Microbiología, Instituto de Salud Carlos III 91837 , Madrid, Spain; 3 Departamento de Microbiología y Genética, Universidad de Salamanca 16779, Salamanca, Spain; Universidade de Sao Paulo, Sao Paulo, Brazil

**Keywords:** *Aspergillus fumigatus*, zinc-metallochaperones, COG0523 proteins, folate biosynthesis, zinc deficiency, ROS production

## Abstract

The filamentous fungus *Aspergillus fumigatus* is equipped with an efficient zinc uptake system that allows this fungus to survive and grow within the very zinc-limiting environment provided by the lungs of immunosuppressed patients. To deal with zinc scarcity, *A. fumigatus* deploys a homeostatic and adaptive response that enables it to scavenge for and uptake zinc from host tissues. Finally, zinc ions are distributed intracellularly and lodged in fungal proteins that require them for normal functioning. It is believed that most zinc-requiring proteins acquire zinc ions to become properly metalated by competition with cellular zinc proteins. However, certain zinc proteins may exhibit inherent thermodynamic and/or physicochemical properties that hamper them from competing for zinc with other proteins during zinc deficiency, such that they can only be properly metalated if aided by specific metallochaperones. In this study, we report a comprehensive approach to the role of the zinc metallochaperones of *A. fumigatus* (MchA, MchB, and MchC) on both fungal physiology during zinc deficiency and fungal pathogenesis. Our data suggest that MchA might play a role in supplying zinc to one or more proteins operating in a biosynthetic pathway that use tetrahydrofolate (THF) as a cofactor; MchB is required for reactive oxygen species (ROS) production as an adaptive response to zinc deficiency, whereas MchC plays a role in THF biosynthesis, most likely by supplying zinc to GTP cyclohydrolase I. This is the first study that provides insights into the role of zinc-metallochaperones in a fungal pathogen and how they could be exploited as antifungal targets.

**IMPORTANCE:**

*Aspergillus fumigatus* is able to suppress nutritional immunity and obtain zinc from the lungs of immunosuppressed patients, allowing it to grow and cause invasive pulmonary aspergillosis. To combat this lethal infection, there is an urgent need for new antifungals. In this regard, tetrahydrofolate (THF) biosynthesis is a promising target. However, antifungal drugs against this process have not been developed yet, likely because only a few antifolates used as antibacterials are also active against a limited number of fungal pathogens. Our research may provide the explanation of the sensitivity to antifolates of those pathogens (*Pneumocystis jirovecii*, *Paracoccidioides brasiliensis,* and *Histoplasma capsulatum*), being that all lack MchC-like proteins. Moreover, we foresee that inhibition of THF biosynthesis in MchC-bearing fungal pathogens could be enhanced by inhibiting MchC activity. Also, our findings suggest the notion that ROS overproduction typically occurring in all cells during zinc deficiency may rely on proper metalation of certain zinc-dependent proteins.

## INTRODUCTION

Although *Aspergillus fumigatus* is a saprophyte filamentous fungus, it exhibits several attributes that enable it to grow within the lungs of immunosuppressed patients and cause one of the deadliest infectious diseases among this group, known as invasive pulmonary aspergillosis (1).

The ability of *A. fumigatus* to surpass the nutritional immunity imposed by the host to obtain zinc from host tissues is one of the most important biological traits that enables *A. fumigatus* to grow as a pathogen and cause disease (2, 3). Referred to zinc homeostasis, the physiological state of the fungal cells can be described either as zinc deficiency or zinc starvation. Zinc deficiency is attained when fungal cells grow in media without a zinc supplement just containing traces of zinc (i.e., zinc-limiting media), whereas zinc starvation is attained after traces of zinc present in zinc-limiting media are consumed due to fungal growth. Zinc deficiency causes a decrease in growth, altered metabolism, and deployment of an adaptive response, whereas zinc starvation causes the stop of cell growth. In contrast, zinc excess may cause mis-metalation of certain proteins, which often leads to their malfunction or inactivation (4).

Under normal physiological conditions, most zinc ions (Zn^2+^) in the host are forming complexes with certain metabolites and proteins, which strongly restricts the access of pathogens to host zinc. Actually, host tissues are so very zinc-limiting that pathogens growing within them show clear signs of zinc deficiency. Thus, *A. fumigatus* deals with zinc deficiency by deploying an adaptive response for zinc acquisition that includes both scavenging and uptake of zinc from host tissues (2, 5–8). However, zinc acquisition by itself might not be sufficient to grant fungal growth within host tissues if Zn^2+^ ions were not delivered to and properly inserted in the proteins that require them for normal functioning. Hence, metalation of zinc-requiring proteins would be the last hindrance to overcome for fungal growth under zinc deficiency.

The principles of selection and acquisition of the correct metal (i.e., metalation) by apoproteins (i.e., proteins without the metal cofactor) have been established mostly based on investigations carried out in prokaryotic cells, but it is assumed that the same principles of metalation are largely applicable to eukaryotic apoproteins (9). Although proteins tend to select essential divalent metal ions with a ranked order of preference that follows the Irving-Williams series (10), the correct metalation *in vivo* occurs because the cytoplasm is a metal-controlled environment where the concentration of free metals is the inverse of the Irving-Williams stability constant series (11, 12). Thus, restriction of metal availability within cells allows proteins to compete with other proteins for limited pools of metals. It has been estimated that 70% of metalloenzymes compete for metals from buffered metal pools, whereas fidelity in metalation of the remaining 30% of metalloenzymes is assisted by specialized proteins referred to as metallochaperones (9, 13, 14). Metallochaperones are metal- and protein-specific and differ in their mechanism of action. It has been estimated that 25% of metalloenzymes acquire metal cofactors preassembled by means of chelatases (e.g., heme [Fe] or chlorophyll [Mg], cobalamine [Co] and molybdenum cofactor [Mo]), whereas the remaining 5% of metalloenzymes are assisted by metallochaperones to discriminate very accurately the most competitive metals (i.e., Cu and Ni) that usually deliver and insert them directly into specific apoproteins (14). Thus, the yeast Cu metallochaperones were described in the first place (15), followed by Ni-metallochaperones for bacterial hydrogenases and ureases (16, 17). However, although Zn is also one of the most competitive metals and the number of Zn-requiring proteins is very high compared to that of the Cu- and Ni-requiring proteins, only a few prokaryotic zinc metallochaperones have been described, namely, ZnuA (18), ZinT (19), ZraP (YjaI) (20), AztD (21), YeiR (22), YjiA (23), ZigA (24), and ZagA (YciC) (25). Half of these prokaryotic zinc metallochaperones (YeiR, YjiA, ZigA, and ZagA) fit within the COG0523 subfamily of P-loop GTPases that belongs to the G3E family of GTPases (26), linked to zinc homeostasis (27). The function of the single COG0523 protein encoded by the *YNR029C/ZNG1* gene of the yeast *Saccharomyces cerevisiae* and the Zng1E human ortholog has also been related to zinc homeostasis during zinc deficiency (28, 29). Unlike yeast and humans, *A. fumigatus* has three different kinds of COG0523 proteins putatively related to zinc homeostasis that we previously named as MchA, MchB, and MchC (for **m**etallo**ch**aperones) (5). In this paper, we report a comprehensive study of the COG0523 genes of *A. fumigatus* on both fungal physiology during zinc deficiency and fungal pathogenesis.

## RESULTS

### COG0523 genes in *Aspergillus fumigatus*

**Fig 1:**
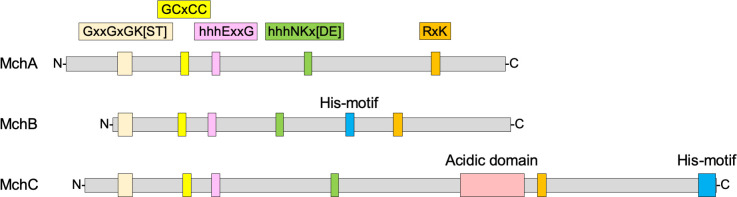
Schematic representation of the primary structure of the Mch proteins of *Aspergillus fumigatus.* The MchA, MchB, and MchC proteins of *A. fumigatus* show in their N-terminal half all highly conserved motifs characterizing the COG0523 subfamily of the G3E family of P-loop GTPases, including the Walter A motif GxxGxGK[ST], the GChCC motif for metal binding (where h is a hydrophobic residue), the Walker B motif (hhhExxG) and the distal hhhNKx[DE] motif. In the C-terminal half of the proteins is also located the conserved RxK motif. His-rich sequences are present in the C-terminal half of MchB and in the C-terminal end of MchC. An acidic domain is shown only in the C-terminal half of MchC.

*Aspergillus fumigatus* has three different genes encoding COG0523 proteins, namely AFUA_2G11720/AFUB_027470 (*mchA*), AFUA_4G07990/AFUB_065100 (*mchB*) and AFUA_8G02620/AFUB_083970 (*mchC*). The Mch proteins of *A. fumigatus* showed in their N-terminal half all highly conserved motifs characterizing the COG0523 subfamily of the G3E family of P-loop GTPases (Fig. 1) (26, 27), but they also exhibit distinctive structural features (see supplementary text and Fig. S1A through D). In addition, a phylogenetic analysis of the Mch proteins revealed that the MchA- and MchC-like proteins represent two different branches of the same clade, whereas the MchB-like proteins belong to a separate clade (Fig. S1E).

### Transcription of *mch* genes is upregulated under very zinc-limiting conditions.

**Fig 2:**
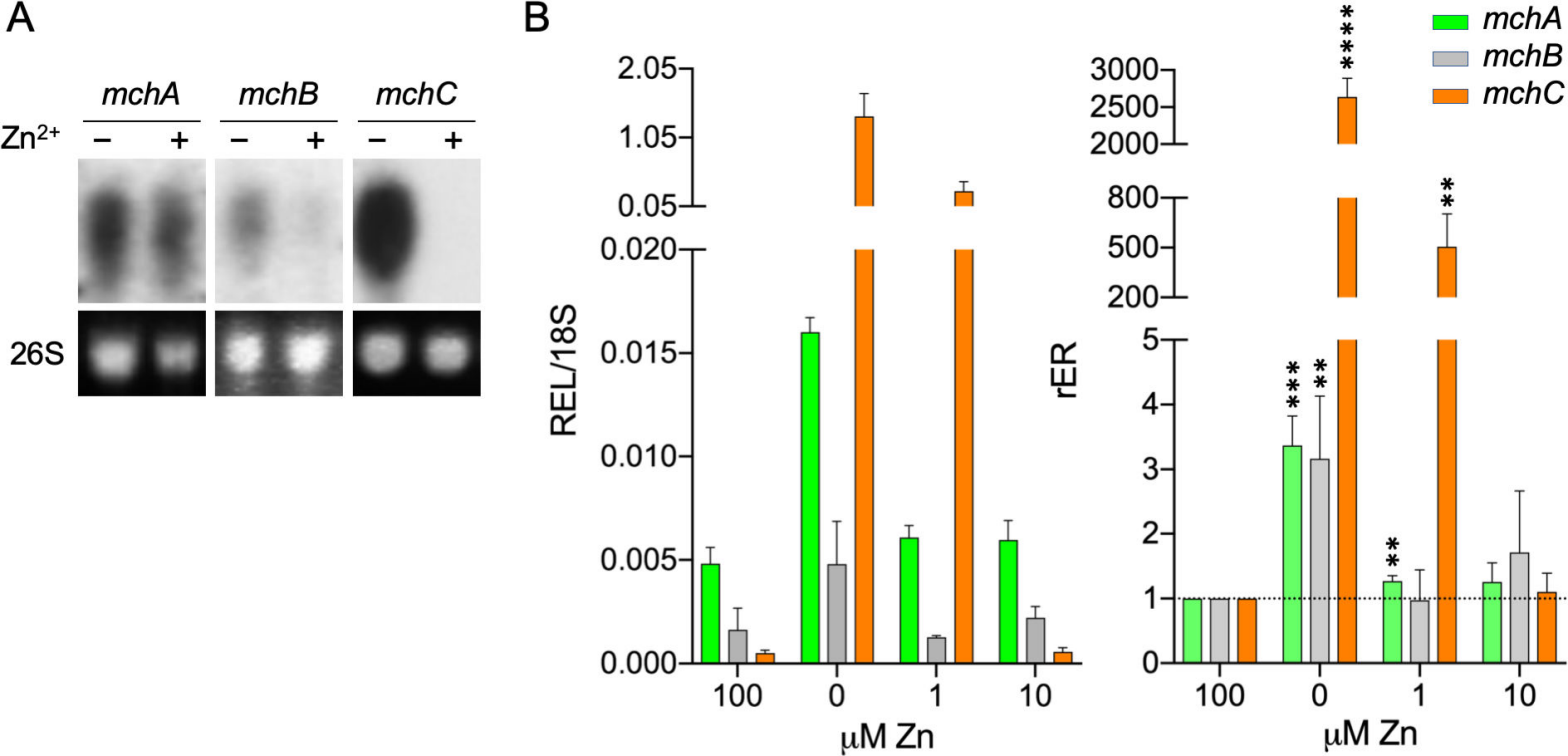
Expression of the *mchA, mchB,* and *mchC* genes. (**A**) The RNA samples used for Northern blot were obtained from the mycelia of a wild-type strain of *Aspergillus fumigatus* inoculated to a density of 1.5 × 10^6^ spores/mL in 100 mL of the SDN–Zn zinc-limiting medium (–Zn) or 50 mL of this medium supplemented with 100 µM zinc (+Zn) and for 20 hours at 37°C with shaking at 200 rpm. The 26S rRNA was included as a loading control. The probes used in Northern blot are listed in Table S6. The nylon membranes probed for the *mchA* and *mchB* genes were exposed for 96 h before developing the films, whereas the nylon membrane probed for the *mchC* gene was exposed for 4 h. (**B**) The RNA samples used for RT-qPCR were obtained from mycelia of a wild-type strain of *A. fumigatus* grown in the liquid SDN–Zn zinc-limiting medium and in this medium supplemented with 1, 10, and 100 µM zinc for 20 hours at 37°C with shaking at 200 rpm. The relative expression level (REL) of these genes was measured by RT-qPCR using the 18S rRNA as an internal reference (left graph). The relative expression ratios (rER) of these genes were calculated with respect to their expression level in the fungus grown in the medium supplemented with 100 µM Zn^2+^ (right graph). RT-qPCR results are the average of three biological replicates. Error bars indicate standard deviation. Data were analyzed statistically by applying a non-paired, two-tailed *t*-test taking the rER of the wild-type strain grown in medium supplemented with 100 µM zinc as a reference. Only statistically significant differences are indicated (*P = 0.05-0.01; **P = 0.01–0.001; ***P = 0.001–0.0001; ****P < 0.0001).

To investigate the effect of zinc availability on the transcription of *mchA*, *mchB,* and *mchC*, we cultured a wild-type strain of *A. fumigatus* under both zinc-limiting and zinc-replete conditions, and the expression of these genes was analyzed by Northern blot (Fig. 2A). The expression levels of both *mchA* and *mchB* were very low, such that their transcripts could be only detected by Northern blot after a very long exposure time. In contrast, the expression level of *mchC* was very high, and it was readily detected by Northern blot after a short exposure time. To ascertain more accurately the effect of zinc on the expressions of these genes, we measured their relative expression levels (REL/18S) by semiquantitative RT-qPCR in the fungus grown in media supplemented with increasing amounts of zinc (0–100 µM) (Fig. 2B). The relative expression ratio (rER) for each gene in the fungus grown without a zinc supplement and supplemented with 1 and 10 µM Zn^2+^ was calculated with respect to their expression levels in the medium supplemented with 100 µM Zn^2+^ (Fig. 2B). Without a zinc supplement, the expression level of both *mchA* and *mchB* increased about threefold, whereas that of *mchC* increased about 2,600-fold because it is the only *mch* gene whose expression is induced directly by ZafA (30). In summary, the expression profile of the *mchA* and *mchB* genes suggests that they are required for optimal fungal growth under both zinc-limiting and zinc-replete conditions. In contrast, *mchC* would be only required for fungal growth during zinc deficiency.

### MchB and MchC are required for fungal growth in liquid media when environmental zinc becomes consumed and fungus undergoes zinc starvation.

**Fig 3:**
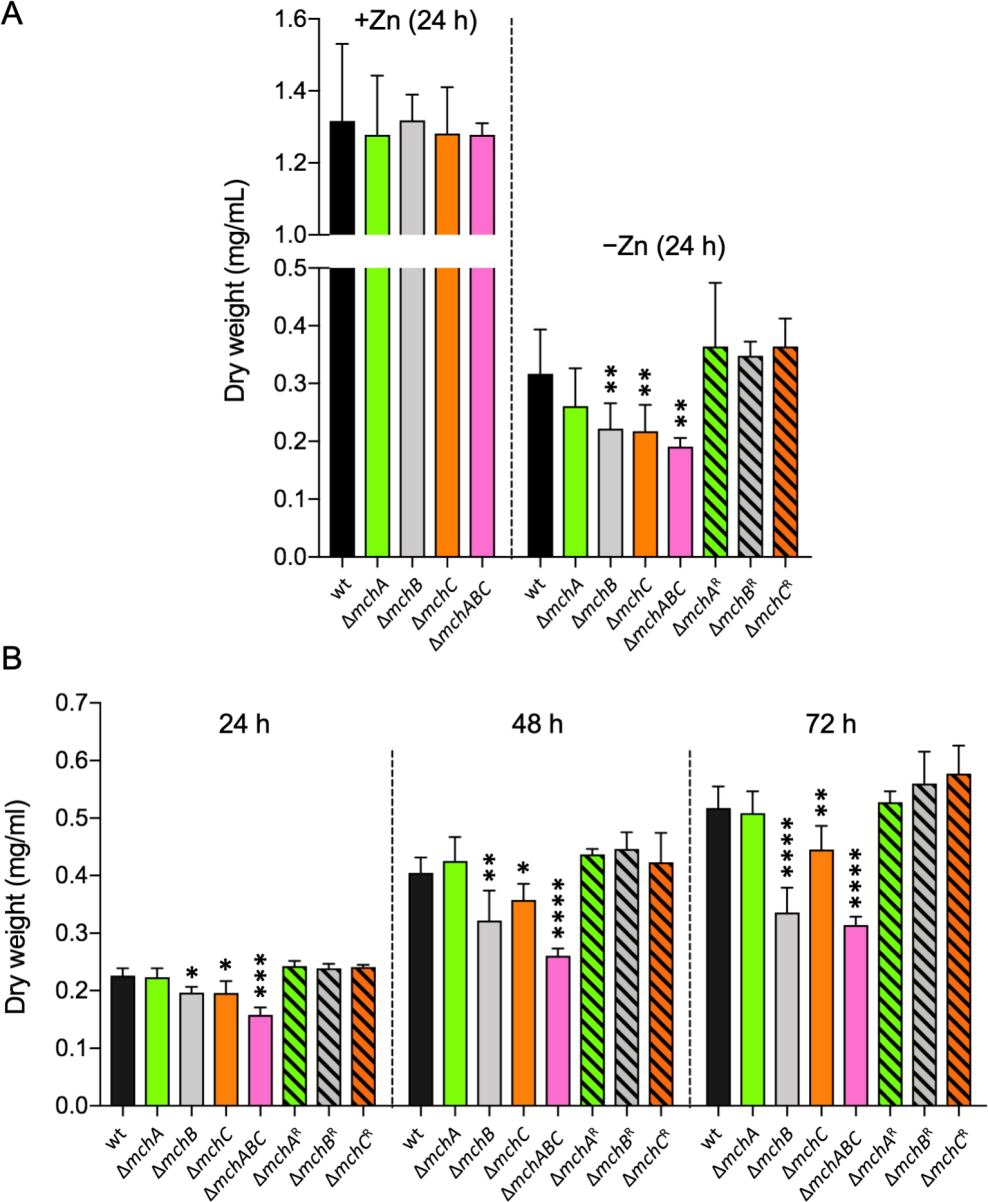
Growth rates and net growth rates of the Δ*mch* mutant strains in liquid media. (**A**) Conidia from the Δ*mchA*, Δ*mchB*, Δ*mchC*, and ΔmchAΔmchBΔmchC (Δ*mchABC*) mutant strains were inoculated in 200 mL of the SDN–Zn zinc-limiting medium and in this medium supplemented with 100 µM Zn^2+^ (SDN + Zn). The reconstituted Δ*mchA*^R^, Δ*mchB,*^R^ and Δ*mchC*^R^ strains were also inoculated in the SDN–Zn medium. (**B**) Conidia from each strain were inoculated in 100 mL of the AMM–Zn zinc-limiting medium. All cultures were inoculated with 5 × 10^5^ conidia/mL and incubated as indicated for 24–72 h at 37°C with shaking at 200 rpm before mycelia were harvested, dried, and weighed. Results are the average of four independent experiments. Bars indicate standard deviation. Data were analyzed statistically by applying a nonpaired, two-tailed *t*-test taking the dry weight of the wild-type strain as a reference. Only statistically significant differences are indicated (*P = 0.05–0.01; **P = 0.01–0.001; ***P = 0.001–0.0001; ****P < 0.0001).

To ascertain the relevance of these genes on both the growth rate and net growth of *A. fumigatus* during zinc deficiency, we generated the Δ*mchA*, Δ*mchB,* and Δ*mchC* mutant strains; the Δ*mchA*Δ*mchB*, Δ*mchA*Δ*mchC,* and Δ*mchB*Δ*mchC* mutant strains; the revertant Δ*mchA*^R^, Δ*mchB,*^R^ and Δ*mchC*^R^ strains; and a Δ*mchA*Δ*mchB*Δ*mchC* mutant strain (Table 1; Fig. S2 and S3) and measured their growth rates and net growths under both zinc-limiting and zinc-replete conditions. No statistically significant differences were observed between the growth rate of every mutant and wild-type strain under both zinc-replete and zinc-limiting conditions (Fig. S4). Similarly, no statistically significant differences were observed between the net growth of the wild-type and that of the Δ*mchA*, Δ*mchB*, Δ*mchC,* and Δ*mchA*Δ*mchB*Δ*mchC* mutants cultured for 24 hours under zinc-replete conditions (Fig. 3A). However, the net growth of the Δ*mchB*, Δ*mchC,* and Δ*mchA*Δ*mchB*Δ*mchC* mutants was significantly lower than that attained by the wild-type after 24 hours of incubation under zinc-limiting conditions (Fig. 3A). If the *mch* genes played a role under extremely zinc-limiting conditions, it would be expected that consumption of traces of zinc during the first 24 hours of incubation increases the growth defect of the mutant strains compared with the wild-type after incubation periods longer than 24 h. Thus, we cultured the same mutant strains under zinc-limiting conditions and measured their net fungal growth after 24, 48, and 72 hours of incubation (Fig. 3B). Deletion of the *mchB* and *mchC* genes significantly reduced the net growth of the Δ*mchB* and Δ*mchC* mutants after 48 and 72 hours of incubation compared to the wild-type. In summary, the lack of any *mch* gene did not change the growth rate measured during the exponential growth phase in liquid media, most likely because this growth phase is reached relatively early in the cultures before the fungus tackles a severe zinc starving condition caused by the own fungal growth. In contrast, deletion of *mchB* and *mchC* reduced the net growth in zinc-limiting liquid cultures, and this reduction increases with the incubation period, which indicates that MchB and MchC are required to sustain fungal growth in the long run when environmental zinc is consumed. Intriguingly, MchA appeared to be not much useful for fungal growth even when fungus undergoes zinc starvation.

**TABLE 1:** *Aspergillus fumigatus* strains used in this study

Strain	Parental strain	Genotype	Brief genotype (uracil auxotrophy)
CEA10	–^*a*^	Wild-type (31)	wt
CEA17	CEA10	*pyrG*^C756G^ (*pyrG1*) (31)	wt (PyrG^–^)
AF121	CEA17	Δ*mchA::hisG-pyrG-hisG*	Δ*mchA*
AF117	CEA17	Δ*mchB:: lacI-pyrG-lacI*	Δ*mchB*
AF114	CEA17	Δ*mchC::PpyrG-pyrG-PpyrG*	Δ*mchC*
AF127	AF121	Δ*mchA::hisG*	Δ*mchA* (PyrG^–^)
AF125	AF117	Δ*mchB::lacI*	Δ*mchB* (PyrG^–^)
AF115	AF114	Δ*mchC::PpyrG*	Δ*mchC* (PyrG^–^)
AF1271	AF127	Δ*mchA::hisG* [*TAP-mchA*]^pyrG^	Δ*mchA*^R^
AF1251	AF125	Δ*mchB::lacI* [*TAP-mchB*]^pyrG^	Δ*mchB*^R^
AF1381	AF115	Δ*mchC::PpyrG* [*TAP-mchC*]^pyrG^	Δ*mchC*^R^
AF131	AF127	Δ*mchA::hisG* Δ*mchB::lacI-pyrG-lacI*	Δ*mchAB*
AF124	AF115	Δ*mchA::hisG-pyrG-hisG*Δ*mchC::PpyrG*	Δ*mchAC*
AF119	AF115	Δ*mchB::lacI-pyrG-lacI*Δ*mchC::PpyrG*	Δ*mchBC*
AF133	AF124	Δ*mchA::hisG*Δ*mchC::PpyrG*	Δ*mchAB* (PyrG^–^)
AF136	AF133	Δ*mchA::hisG*Δ*mchB::lacI-pyrG-lacI*Δ*mchC::PpyrG*	Δ*mchABC*
AF145	AF136	Δ*mchA::hisG*Δ*mchC::PpyrG*Δ*mchB::lacI*	Δ*mchABC* (PyrG^–^)
AF147	AF145	Δ*mchA::hisG*Δ*mchC::PpyrG*Δ*mchB::lacI*	Δ*mchABC* (PyrG^+^ isogenic to CEA10)

^*a*^ "–”, not applicable.

### MchA, MchB, and MchC are required for normal fungal growth and development on solid media when zinc deficiency becomes severe.

**Fig 4:**
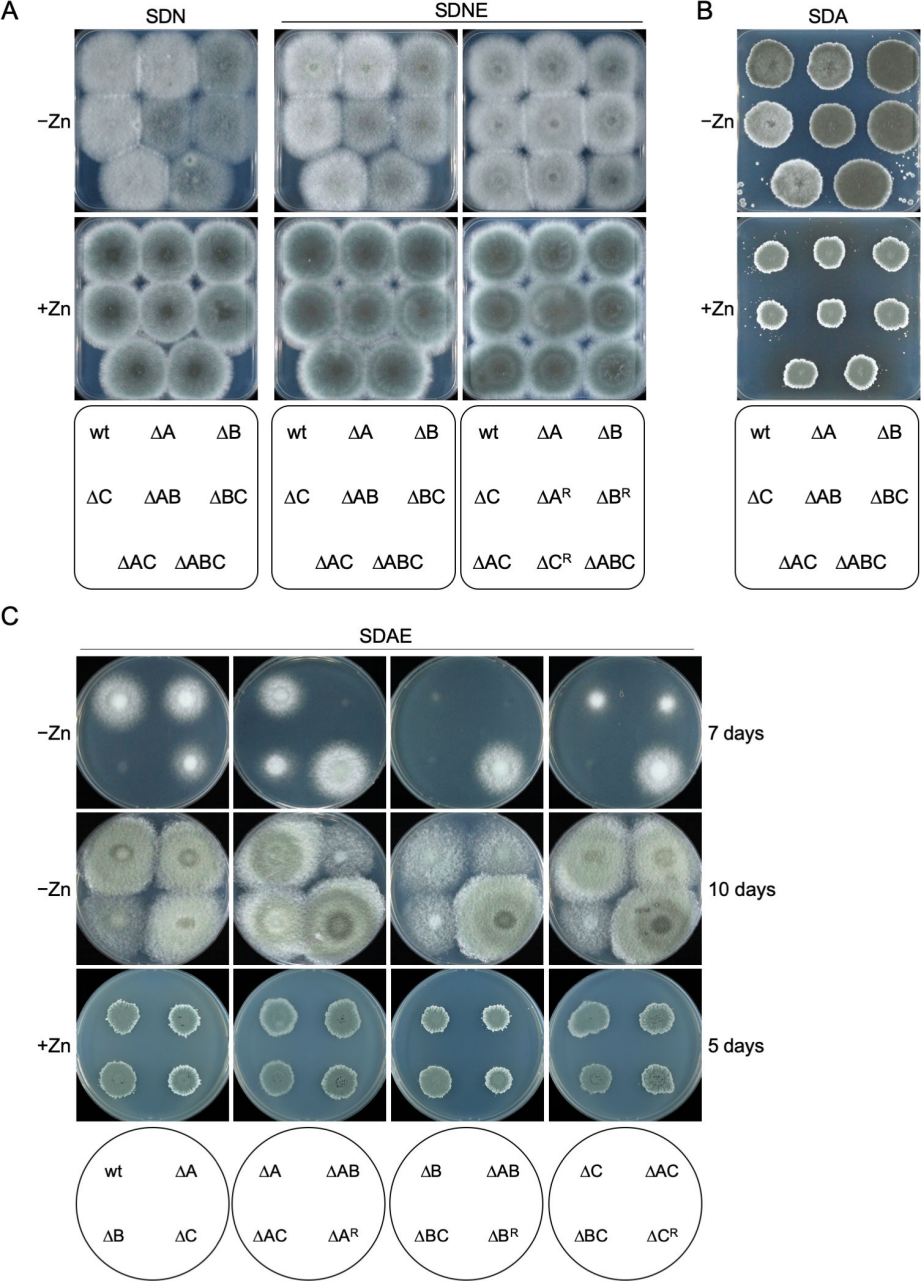
Phenotypic analysis of the Δ*mch* mutant strains growing on solid media. (**A**) Conidia of each Δ*mch* mutant strain were spotted onto agar plates of the slightly alkaline SDN–Zn and on this medium supplemented with 0.25 mM EDTA (–Zn) and/or 100 µM Zn^2+^ (+Zn). (**B**) Conidia of each Δ*mch* mutant strain were spotted onto agar plates of the acidic SDA–Zn medium (–Zn) and on plates of this medium supplemented with 100 µM Zn^2+^ (+Zn). (**C**) Conidia of each Δ*mch* mutant strain were spotted onto agar plates of the acidic SDAE–Zn zinc-limiting medium (–Zn) and on plates of this medium supplemented with 100 µM Zn^2+^ (+Zn). In the acidic SDA–Zn medium, NH_4_^+^ uptake by fungal cells leads to acidification of the medium, such that the pH around fungal cells may drop below pH 2.5 (33). When EDTA is present in this medium, its acidification favors that nearly 20% of EDTA becomes protonated (H_4_Y) (32), which can readily enter the cells; when inside the cells, EDTA ionizes at the neutral pH of the cytoplasm and recovers its chelating properties, leading to the seizure of intracellular metal ions. Therefore, the growability of fungal strains with any deficiency in zinc acquisition or allocation is more impacted by EDTA on the acidic SDAE–Zn zinc-limiting solid medium than on the alkaline SDNE–Zn zinc-limiting solid medium. All agar plates were inoculated with spots of 5 µL containing 10^3^ conidia and incubated at 37°C in a humid atmosphere for 3 days (panel A), 5 days (panel B), or 5–10 days (panel C) before pictures were taken.

To enhance the detection of growth deficiencies under extremely zinc-limiting conditions linked to deletion of the different *mch* genes, we cultured all Δ*mch* mutant strains onto agar plates of the slightly alkaline SDN–Zn medium (pH 7.5) and on this medium supplemented with 250 µM EDTA (i.e., SDNE–Zn). In this alkaline medium, a high fraction of EDTA (>90%) is highly charged (HY^2–^/HY^3–^) (32) and stays extracellularly where it binds zinc with high affinity. Most of the Δ*mch* mutant strains grew onto these solid media as a wild-type strain, except the Δ*mchB* mutant strains that, under zinc-limiting conditions, formed colonies much less dense than that developed by the wild-type or any other Δ*mch* mutant, such that they appeared darkened when photographed on a black background (Fig. 4A). In addition, we also tested the growth ability of the Δ*mch* mutants onto agar plates of the acidic SDA–Zn medium (pH 4.5) (Fig. 4B) and on this medium supplemented with 250 µM EDTA (i.e., SDAE–Zn) (Fig. 4C). When all strains had completed the development of their colonies after 5 days of incubation onto the acidic SDA–Zn solid medium, compared to the wild-type, the Δ*mchA*, Δ*mchC,* and Δ*mchA*Δ*mchC* mutants showed pale colonies (darkening of colonies by *mchB* deletion counteracted the pale aspect conferred by deletion of *mchA* or *mchC* in the Δ*mchB* mutants) (Fig. 4B). In contrast, onto the acidic SDAE–Zn medium, both the wild-type and all Δ*mch* mutants initially grew very slowly until they became adapted to grow on this medium. Thus, the growth ability of the Δ*mchA,* Δ*mchC,* and Δ*mchA*Δ*mchC* mutants after 7 days of incubation was reduced to different extents compared to that of the wild-type, whereas all strains carrying the Δ*mchB* deletion were just starting to grow (Fig. 4C). Once all strains had completed the development of their colonies after 10 days of incubation, the Δ*mchB* strains formed very loose and fluffy colonies (Fig. 4C). In summary, the phenotypic analysis carried out under appropriate conditions to achieve a high degree of intracellular zinc deficiency compatible with fungal growth (i.e., in acidic media supplemented with EDTA) revealed that the three Mch proteins of *A. fumigatus* are required to different extents for optimal fungal growth on solid media when zinc deficiency becomes severe.

### MchB is linked to ROS production under zinc-limiting conditions, while deletion of *mchB* paradoxically increases the sensitivity to oxidative stress induced by H_2_O._2_

**Fig 5:**
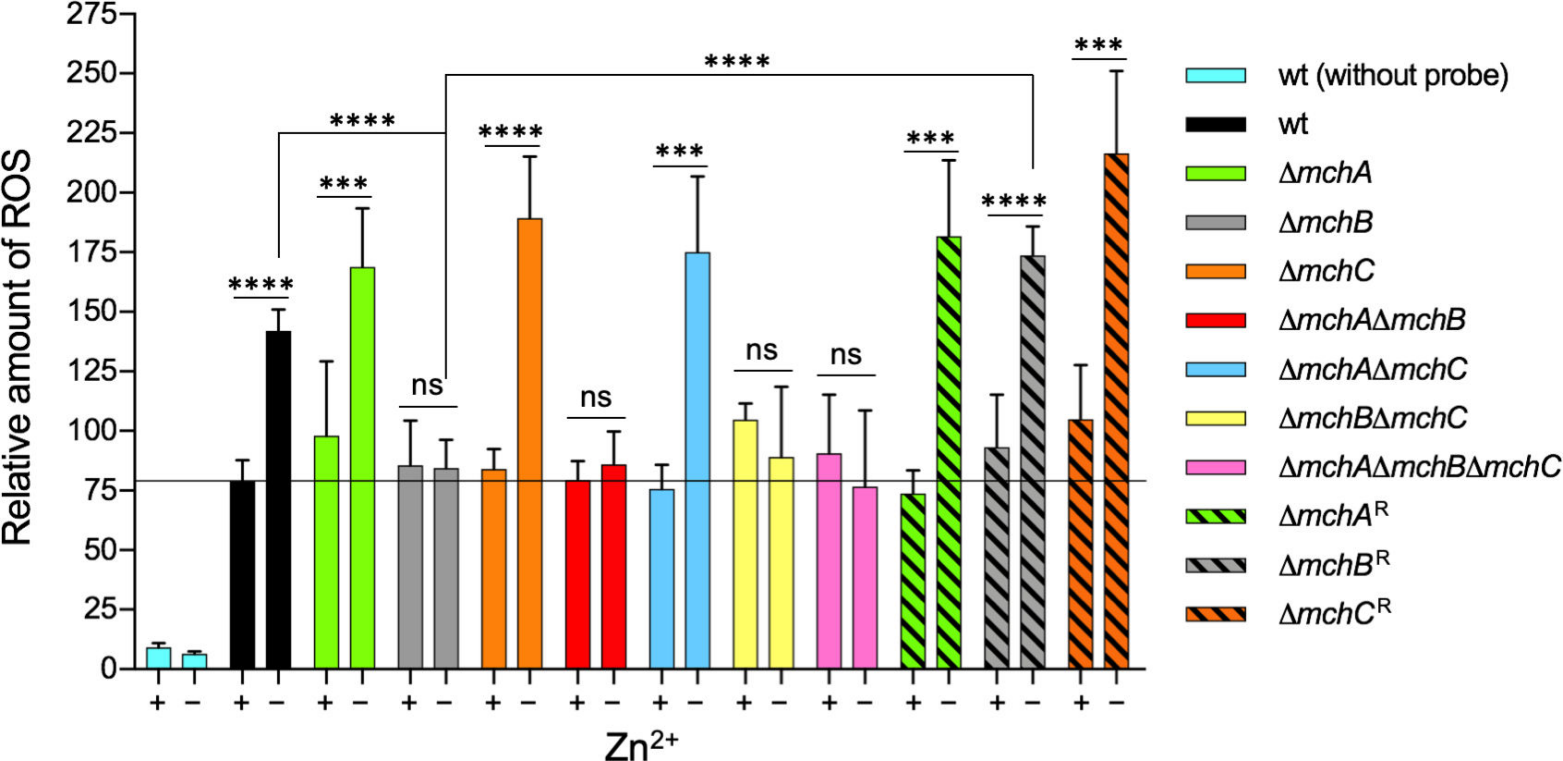
Relative quantification of ROS production by Δ*mch* mutant strains. The fungal strains were cultured in the AMM zinc-limiting medium (–Zn) and in this medium supplemented with 100 µM Zn^2+^ (+Zn). All cultures were incubated at 37°C and 200 rpm for 22 h before adding the DCFH_2_-DA probe at a final concentration of 10 µM. Mycelia were harvested 2.5 h after adding the fluorescent probe, and ROS production was measured as described in the *Material and Methods* section. The relative amount of ROS refers to fluorescence units normalized with respect to protein concentration in each sample. Results are the average of four independent experiments. Bars indicated standard deviation. Blue bars refer to values measured in the wild-type strain in the absence of probe. Data were analyzed statistically by applying a non-paired, two-tailed *t*-test taking the dry weight of the wild-type strain as a reference (ns, not significant; *P = 0.05–0.01; **P = 0.01–0.001; ***P = 0.001–0.0001; ****P < 0.0001).

Increase in ROS production is one of the major adaptive responses to zinc deficiency. However, both the underlying molecular mechanism and the biological significance of this adaptive response are completely unknown, though several hypotheses to explain this phenomenon have been proposed (34). As expected, the relative amount of ROS produced by most Δ*mch* mutant strains was significantly higher under zinc-limiting than under zinc-replete conditions. However, strains carrying the Δ*mchB* deletion produced the same amount of ROS under zinc-limiting conditions as in zinc-replete media, i.e., the Δ*mchB* mutant strains failed in producing ROS as an adaptive response to zinc deficiency (Fig. 5). In an attempt to explain this, we hypothesized that ROS production in *A. fumigatus* under zinc-limiting conditions could be paradoxically an adaptive response to oxidative stress promoted by overproduction of H_2_O_2_ during zinc deficiency. Thus, to ascertain whether the Δ*mch* mutant strains showed any deficiency in the adaptive response to exogenously H_2_O_2_-induced oxidative stress, each mutant was seeded onto both zinc-limiting and zinc-replete agar media with a well in the middle filled with 0.3 mL of a fresh 3% H_2_O_2_ solution. The inhibition zone diameters were measured after 48 and 60 hours of incubation (Fig. S5). Under zinc-replete conditions, and for the same incubation period, no significant differences were observed between the inhibition zone diameter of each mutant strain and that of the wild-type (Fig. S5A). In contrast, under zinc-limiting conditions, and for the same incubation period, the inhibition zone diameters produced by strains carrying the Δ*mchB* deletion were significantly higher than that produced by the wild-type strain or any other mutant strain (Fig. S5B). This showed that deletion of *mchB* increases the fungal sensitivity to H_2_O_2_ during zinc deficiency. In summary, these results suggested that MchB is required for increasing ROS production as an adaptive fungal response to grow in zinc-limiting media, most likely in response to oxidative stress caused by H_2_O_2_ produced during zinc deficiency.

### MchC is required for dihydrofolate biosynthesis during zinc deficiency.

The prokaryotic COG0523 proteins ZigA from *Acinetobacter baumanii* and ZagA from *Bacillus subtilis* (25, 35) are far more closely related to MchC than the MchA protein (Fig. S6). ZigA activates a zinc-dependent histidase (HutH) that is required during zinc deficiency to break down histidine (His) to allow both bacterial growth with His as a sole carbon and to mobilize the His-associated zinc pool (24). In contrast, ZagA delivers zinc to the zinc-dependent GTP cyclohydrolase I (GCYH-IA) MtrA/FolE1 to sustain tetrahydrofolate (THF) biosynthesis (25).

Unlike *Acinetobacter*, *Aspergillus* cannot use His as a sole carbon source. However, *Aspergillus* can use His as the sole nitrogen source because it harnesses ammonia released upon breakdown of His by its histidase (36). Hence, we reasoned that if MchC provides zinc to the histidase of *A. fumigatus* (AFUA_2G09110), a Δ*mchC* mutant strain, compared to a wild-type strain, would grow poorly under zinc-limiting conditions using L-His as the sole nitrogen source. However, all Δ*mch* mutant strains carrying the Δ*mchC* deletion grew as a wild type in zinc-limiting media with L-His as the sole nitrogen source. Hence, *mchC* does not appear to be involved in His catabolism under zinc-limiting conditions.

**Fig 6:**
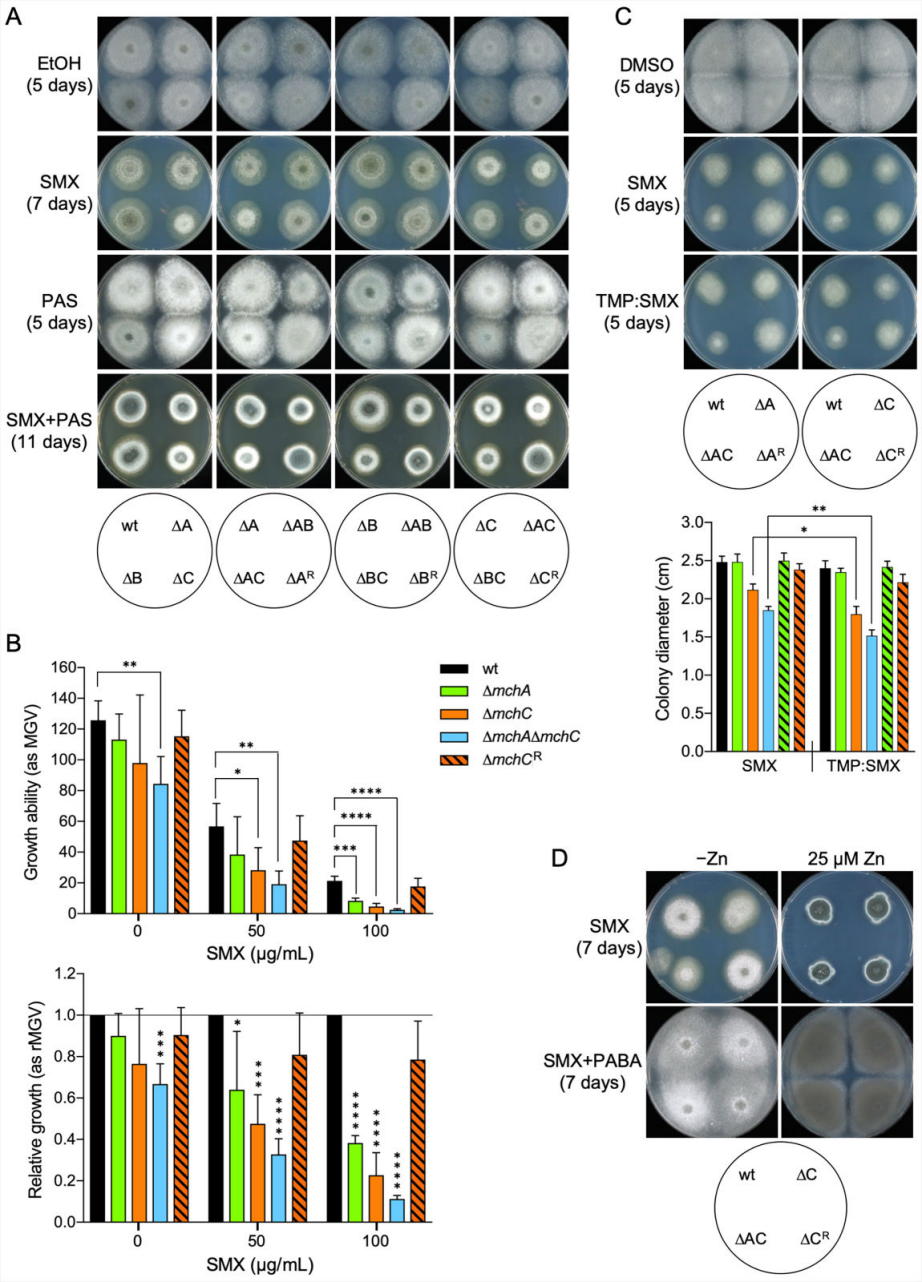
Effect of inhibitors of THF biosynthesis on the growth ability of the Δ*mch* mutant strains. (**A**) The Δ*mch* mutant strains were spotted onto agar plates of the AMME–Zn zinc-limiting medium supplemented with 50 µg/mL SMX, 0.6 mg/mL PAS, 50 µg/mL SMX plus 0.45 mg/mL PAS, or just 1.3% (vol/vol) ethanol (as vehicle for SMX and PAS). (**B**) The measurement of the effect of SMX on fungal growth was carried out in microcultures of 1.0 mL dispensed in 24-well culture plates containing 0.8 × AMME–Zn (750 µL) medium, 0.05% Tween-20 (10 µL), 10^4^ conidia (40 µL), 145 µL sterile water and 5 µL ethanol (as vehicle for SMX), or 50 µg of SMX (stock 10 mg/mL in ethanol) or 100 µg of SMX (stock 20 mg/mL in ethanol). Plates were incubated at 37°C in a humid atmosphere for 5 days before scanning the plates. The growth ability of all mutant strains was measured as mean gray values (MGV) using ImageJ/Fiji software (upper panel). Relative growth abilities were calculated taking wild-type MGV data in the absence of SMX or in the presence of 50 or 100 µg/mL SMX as references (lower panel). (**C**) Conidia from the wild-type, Δ*mchA*, Δ*mchC*, Δ*mchA*Δ*mchC,* Δ*mchA,*^R^ and Δ*mchC*^R^ strains were spotted onto agar plates of the AMME–Zn medium supplemented with 50 µg/mL SMX, TMP:SMX (1:5), i.e., 10 µg/mL TMP plus 50 µg/mL SMX or just 0.3% (vol/vol) DMSO (as vehicle for SMX and TMP). (**D**) Conidia from the wild-type, Δ*mchC*, Δ*mchA*Δ*mchC,* and Δ*mchC*^R^ strains were spotted onto agar plates of the media AMME–Zn and AMME +25 µM Zn^2+^, both supplemented with 50 µg/mL SMX or with 50 µg/mL SMX plus 5 µg/mL PABA. All plates were inoculated with spots of 5 µL containing 10^3^ conidia and incubated at 37°C in a humid atmosphere for 5–11 days as indicated, before pictures were taken. Results shown in panels **B** and **C** are the average of five independent experiments. Bars indicate standard deviation. Data were analyzed statistically by applying a non-paired, two-tailed *t*-test taking the dry weight of the wild-type strain as a reference (*P = 0.05–0.01; **P = 0.01–0.001; ***P = 0.001–0.0001; ****P < 0.0001).

Unlike *Bacillus*, *Aspergillus* lacks a zinc-independent GCYH-I and only has the canonical enzyme, zinc-dependent GCYH-I (GchA) encoded by the AFUA_5G03140/*gchA* gene. This enzyme converts GTP into 7,8-dihydroneopterin triphosphate (DHN-P_3_) (Fig. S7). This metabolite is the precursor of 6-hydroxymethyl-7,8-dihydropterin diphosphate (HMDHP-P_2_) that is condensed with *p*-aminobenzoic acid (PABA) to generate 7,8-dihydropteroate (DHP). It has been demonstrated that sulfamethoxazole (SMX) competes with PABA for ligation with HMDHP-P_2_ and forms a dead-end complex with this metabolite, such that either an increase in PABA synthesis or a PABA supplement confers SMX resistance (37). We hypothesized that if MchC supplied zinc to the GCYH-I enzyme of *A. fumigatus* during zinc deficiency, the fungal strains carrying the Δ*mchC* deletion should exhibit a GCYH-I activity lower than that of the wild-type strain, such that the Δ*mchC* mutant strains should exhibit a growth defect in the presence of SMX that could be rescued, at least partially, by PABA and totally by zinc. To investigate the putative role of MchC on THF biosynthesis, we cultured the Δ*mch* mutant strains onto the AMME–Zn zinc-limiting medium supplemented with 50 µg/mL SMX (Fig. 6A, upper panels). All fungal strains grew in the presence of SMX slower than in the absence of SMX. However, we noticed that the fungal strains lacking *mchC* grew more slowly than the wild-type strain (in the Δ*mchB*Δ*mchC* mutant strain, deletion of *mchB* disguised the aspect of the colonies of the Δ*mchC* mutant strain in the presence of SMX). To investigate further whether the Δ*mchC* mutant strains showed a growth defect in the presence of other PABA analogs, such as *p*-aminosalicylic acid (PAS) (Fig. S7), we also cultured the Δ*mch* mutant strains in the AMME–Zn zinc-limiting medium supplemented with 0.5 mg/mL PAS (Fig. 6A, middle panels). Indeed, in the presence of PAS, the fungal strains carrying the Δ*mchC* mutation formed colonies slightly less pigmented than those developed by the wild-type and the Δ*mchC* revertant strain. In addition, we also noticed that strains lacking *mchA* developed fluffy colonies and that the Δ*mchA*Δ*mchC* mutant strain formed very white colonies that appeared even whiter than the Δ*mchC* mutant strain. This was more clearly observed in the AMME–Zn medium supplemented with 0.5 mg/mL PAS plus 50 µg/mL SMX (Fig. 6A, lower panels). To measure accurately the effect of SMX on the growth ability of the Δ*mchA*, Δ*mchC*, Δ*mchA*Δ*mchC,* and Δ*mchC*^R^ mutant strains, mycelial growth of these strains was compared with that of a wild-type after 5 days of incubation in the liquid AMME–Zn medium supplemented with 50 and 100 µg/mL SMX (Fig. 6B). In the absence of SMX, only the Δ*mchA*Δ*mchC* mutant showed a significant growth defect compared to the wild-type. However, as the concentration of SMX increased, the growth ability of the Δ*mchC* and Δ*mchA*Δ*mchC* strains significantly reduced compared with that of the wild-type. Thus, a supplement of 50 µg/mL SMX reduced the growth ability of the Δ*mchC* and Δ*mchA*Δ*mchC* mutant strains, compared to the wild-type, by twofold and threefold, respectively, whereas a supplement of 100 µg/mL SMX decreased their growth ability, compared to the wild-type, by 3.5-fold and 10-fold, respectively. In addition, it was also noteworthy that a supplement of 100 µg/mL SMX diminished the growth ability of the Δ*mchA* mutant strain, compared to the wild-type, by 2.5-fold (Fig. 6B).

The combination 1:5 of trimethoprim (TMP) and SMX (TMP:SMX), also known as cotrimoxazole, is effective to treat pneumonia caused by the fungal pathogen *Pneumocystis jirovecii* (38). TMP not only inhibits the enzyme 7,8-dihydrofolate reductase but also potentiates SMX activity by inhibiting HMDHP-P_2_ biosynthesis (39). Although it is known that TMP does not disturb apparently the growth of a wild-type strain of *A. fumigatus* (40), it could be possible that TMP inhibits to a certain extent the growth ability of the Δ*mchA*, Δ*mchC*, or ΔmchAΔmchC mutant strains. To ascertain further the relevance of the *mchA* and *mchC* genes in THF biosynthesis, we tested the effect of TMP on the growth ability of the Δ*mchA*, Δ*mchC,* and Δ*mchA*Δ*mchC* strains. However, TMP did not apparently have any effect on the fungal growth ability of these mutants (data not shown), which indicated that TMP alone either was completely harmless against these mutants or had a minor effect on THF biosynthesis with a negligible impact on fungal physiology. To investigate the later possibility, we tested the effect of the combination TMP:SMX (1:5) on the growth ability of the Δ*mchA*, Δ*mchC,* and Δ*mchA*Δ*mchC* mutants (Fig. 6C). This combination did not change the growth ability of the Δ*mchA* mutant, but it significantly reduced the growth of the Δ*mchC* and Δ*mchA*Δ*mchC* mutant strains' colonies compared to treatment with SMX alone. Finally, to confirm that the growth defect of the Δ*mchC* and Δ*mchA*Δ*mchC* mutants observed in the presence of SMX was due to a defect in THF biosynthesis, we supplemented this medium with PABA to suppress the effect of SMX, and/or with Zn^2+^ to allow zinc acquisition by GchA without being assisted by MchC (Fig. 6D). As shown, the PABA supplement partially recovered at a similar extent to the growth ability of both strains under zinc-limiting conditions in the presence of SMX, such that the colonies of the Δ*mchA*Δ*mchC* mutant strain, compared to that of the Δ*mchC* colonies, exhibited a lower level of conidiophore differentiation, as visualized by the less pigmented colonies. In contrast, a Zn^2+^ supplement restored completely the growth ability of both strains at the wild-type level both in the presence and absence of PABA. In summary, taken together, these results indicated that MchC is required for optimal THF biosynthesis under zinc-limiting conditions. In contrast, the negligible effect of antifolates on the growth ability of the Δ*mchA* mutant strain, along with the fact that the inhibitory effect of antifolates is higher against a Δ*mchA*Δ*mchC* mutant strain than against a Δ*mchC* mutant strain, strongly suggests that MchA is most likely related to metalation of a protein involved in a THF-dependent process rather than with THF biosynthesis itself.

### The *mch* genes are not required for fungal virulence in a leucopenic murine model of IPA.

**Fig 7:**
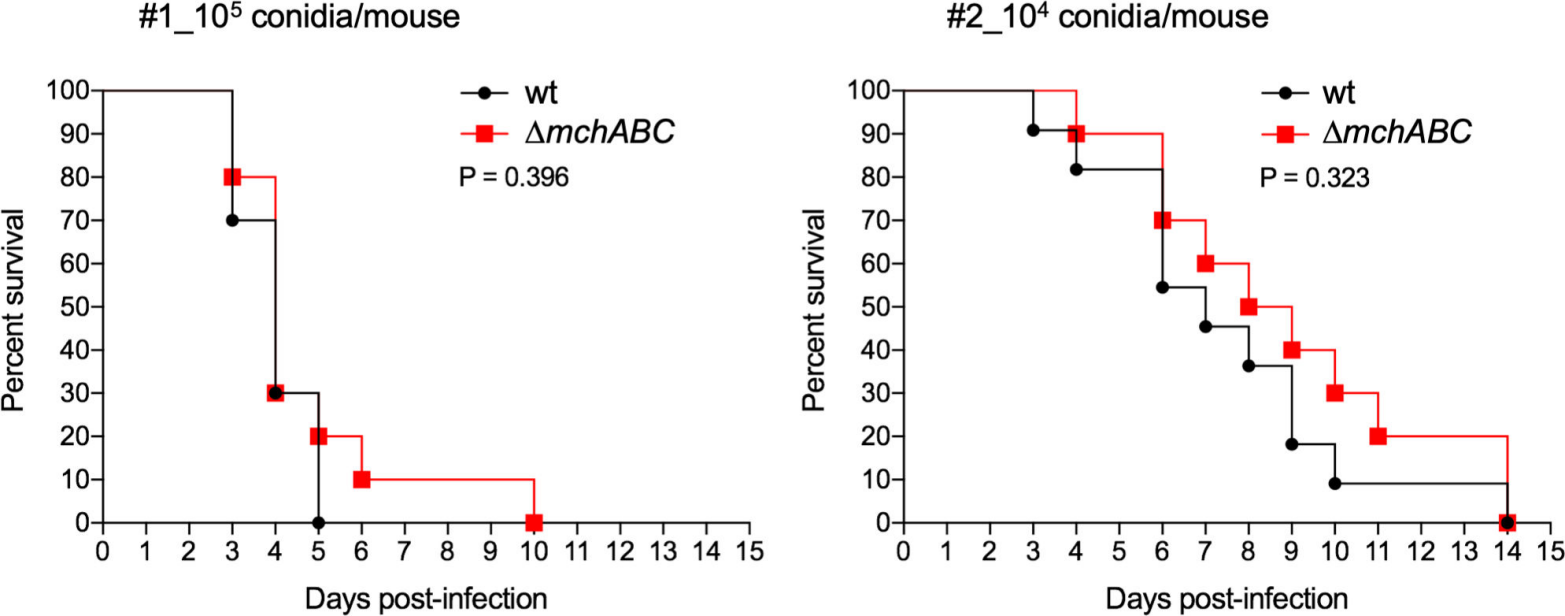
Survival of mice in a leucopenic murine model of invasive pulmonary aspergillosis. Conidia from the wild-type CEA10 strain and the uracil-uridine-prototrophic and isogenic to *pyrG*Δ*mchA*Δ*mchB*Δ*mchC* mutant strain (AF147) were used to inoculate two different cohorts of mice. Mice from cohort #1 were inoculated with 10^5^ conidia per mouse, and mice from cohort #2 were inoculated with 10^4^ conidia per mouse. Survival of mice was followed for 15 days. Survival curves were created with the Prism 7.0 Software, using the product-limit method of Kaplan-Meier, and were compared using the log-rank test.

Previous results indicated that MchA is putatively involved in normal functioning of a THF-dependent process, MchB was required for ROS production as an adaptive response to zinc deficiency, and MchC appears to have a role in biosynthesis of THF during zinc deficiency. Given that living host tissues provide a zinc-limiting condition, we reasoned that virulence of a Δ*mchA*Δ*mchB*Δ*mchC* mutant strain could be severely impaired, and it prompted us to test its virulence in a leucopenic murine model of IPA, as described in the Material and Methods section. However, no statistically significant differences were observed between the survival of mice inoculated with the wild-type and the Δ*mchA*Δ*mchB*Δ*mchC* strain (Fig. 7). Hence, the *mch* genes are not apparently essential for fungal virulence in a leucopenic murine model of IPA.

## DISCUSSION

The chaperones of the COG0523 subfamily of P-loop GTPases are universally linked to zinc homeostasis in both prokaryotic and eukaryotic organisms (27). The abundance of most zinc-dependent enzymes in cells not only decreases during zinc deficiency due to reduction in mRNA levels and/or proteolysis, but also a fraction of every zinc-protein is unmetalated (41). Despite this, the metalated fraction of most zinc proteins suffices the metabolic demand for normal cell growth under zinc deficiency. However, the amount of certain zinc proteins under zinc-limiting conditions is too low compared to zinc-replete conditions, such that the relative amount of these metalated proteins could be insufficient to meet the cellular demand unless zinc acquisition is supported by specific zinc metallochaperones. For example, the amount of MetAP-1 of *S. cerevisiae* (Map1) is more reduced under zinc deficiency (41). Although the amount of Map1 molecules is very low under zinc-limiting conditions, they are metalated properly by the Zng1 COG0523 metallochaperone (28); otherwise, the relative amount of active Map1 molecules would be too low to fulfill the methionine aminopeptidase activity for normal yeast growth during zinc deficiency.

In an attempt to ascertain whether any *mch* gene of *A. fumigatus* was able to rescue the lack of *ZNG1* in *S. cerevisiae*, we carried out a complementation analysis (supplementary text and Fig. S8 and S9). However, the expression of none of the *mch* genes was able to complement the lack of *ZNG1* in yeast. The most plausible explanation is that the actual function of the Mch protein of *A. fumigatus* is related with that of Zng1 in *S. cerevisiae*, not even that of MchA, which is the most closely related protein to Zng1. Nevertheless, it could also be possible that the Mch proteins cannot interact properly with the yeast Zng1 target proteins. In this regard, we cannot preclude that MchA is involved in metalation of the MetAP-1 in *A. fumigatus* under zinc-limiting conditions, as is Zng1 in yeast. However, the high level of redundancy of MetAPs in *A. fumigatus* complicates showing the putative role of MchA on the MetAP-1 activity. Indeed, *A. fumigatus* bears two MetAP-1 (AFUA_6G07330 and AFUA_8G00460/fpaI) and three MetAP-2 (AFUA_2G01750, AFUA_4G06930, and AFUA_8G00410/fpaII). The MetAP-1 proteins of *A. fumigatus* show 65.2% identity and 80.7% similarity. Provided that MchA had a role in Zn^2+^ acquisition by MetAP-1 proteins, most likely both MetAP-1 proteins of *A. fumigatus* rely on MchA for zinc acquisition. Besides, it is worth noting that one MetAP of each type in *A. fumigatus* is encoded by genes located in the fumagillin biosynthetic (fpaI and fpaII), although they are not involved in fumagillin biosynthesis (42). Actually, fumagillin and its structural analog TNP-470 strongly and specifically inhibit human MetAP-2 (43). In addition, anthranilic acid sulfonamides, such as 2-(4′-chlorophenylsulfonamido) benzoic acid (CPS-BA), reversibly inhibit the human MetAP-2 (44). Thus, it would be expected that simultaneous inhibition of the *A. fumigatus* MetAP-2 enzymes by treatment with TNP-470 or CPS-BA would render the fungus more suitable to the lack of *mchA*, particularly under zinc-limiting conditions. However, these drugs did not have any effect at all on the fungal growth ability of a Δ*mchA* mutant strain (Fig. S10). Nevertheless, we cannot totally preclude a role of MchA in metalation of MetAP-1 enzymes in *A. fumigatus* since these drugs may not readily enter the fungal cells or inhibit fungal MetAP-2 as efficiently as they inhibit the human MetAP-2. In addition, although *a priori* both MetAP-1 proteins of *A. fumigatus* could be MchA targets, the effect of the Δ*mchA* deletion on fungal growth under zinc-limiting conditions could be hidden if only one of these MetAP-1 enzymes is the actual target of MchA. In addition, this would explain that the Δ*mchA* mutation does not reduce significantly fungal growth in a liquid zinc-limiting medium after a long time of incubation (Fig. 3B), and that only in an acidic zinc-limiting medium supplemented with EDTA does the ΔmchA mutation impact fungal growth very slightly compared to the wild-type (Fig. 4C). Moreover, these results would be compatible with a role of MchA in metalation of one or more proteins operating in a biosynthetic pathway that uses THF as a cofactor (e.g., biosynthesis of purines and thymidylate, pantothenate, serine, glycine, or methionine), as suggested previously.

Regarding the putative function of MchB, our results clearly indicated that this putative metallochaperone may upregulate ROS production as an adaptive response to oxidative stress caused by H_2_O_2_ produced intracellularly during zinc deficiency, which would be consistent with the reduced growth ability under zinc-limiting conditions of strains lacking *mchB* (Fig. 3 and 4). In this regard, it is well known that *S. cerevisiae* increases slightly the production of superoxide radicals in the mitochondria as a defense mechanism in response to H_2_O_2_-induced stress (45). However, neither H_2_O_2_ nor O_2_^•–^ oxidize directly the DCFH probe used to detect ROS (46). Hence, it is unknown whether total ROS detected using DCFH is generated in its entirety as a consequence of zinc deficiency or it is the sum of H_2_O_2_ produced during zinc deficiency in addition to ROS produced as a defense mechanism against it.

Based on phenotypic analyses of the Δ*mchC* mutant strain in the presence of different inhibitors of THF biosynthesis (TMP and SMX), we concluded that MchC is most likely required for THF biosynthesis during zinc starvation. For instance, deletion of *nudB* in *E. coli*, which encodes the enzyme that catalyzes the second step of THF biosynthesis (Fig. S7), showed over 300-fold enhanced susceptibility to SMX and 60-fold enhanced susceptibility to TMP (39). By analogy with this and provided that GchA of *A. fumigatus*, which catalyzes the first step of THF biosynthesis (Fig. S7), is a MchC target, the fact that TMP slightly reduced the growth ability of the Δ*mchC* mutant in the presence of SMX (Fig. 6C) is consistent with an enhanced susceptibility to SMX by inhibiting HMDHP-P_2_ biosynthesis at a low level in a TMP-mediated manner. Therefore, the partial inactivation of GchA in the Δ*mchC* mutant agrees with the enhanced susceptibility of this strain to TMP in the presence of SMX during zinc deficiency. In addition, restoration of normal fungal growth at the wild-type level under zinc-replete conditions in the presence of SMX is consistent with the importance of zinc for activity of the GTP cyclohydrolase I (47), which is the only Zn-dependent enzyme of the pathway for THF biosynthesis. Taken together, these results support the notion that GTP cyclohydrolase I is a client protein of MchC in *A. fumigatus* during zinc deficiency. It is likely that MchC delivers zinc to GchA in *A. fumigatus* as the YciC/ZagA metallochaperone delivers zinc to the zinc-dependent GTP cyclohydrolase I (GCYH-IA, FolE) of *B. subtilis* during zinc starvation (25). If, as predicted, MchC is required during zinc deficiency for the GchA enzyme to become fully active, it would be expected that a Δ*mchC* mutant would be unable to grow under zinc-limiting conditions since the GTP cyclohydrolase I activity is essential for fungal growth. However, the Δ*mchC* mutant strain only shows a minor growth defect in both liquid media (Fig. 3) and onto solid acidic zinc-limiting media supplemented with EDTA (Fig. 4C). This indicates that most GchA molecules must be able to acquire Zn^2+^ without being assisted by MchC, though this metallochaperone would facilitate this process when zinc availability becomes extremely low.

Regarding the relevance of the *mch* genes in fungal pathogenesis, it was disconcerting at first sight that a Δ*mchA*Δ*mchB*Δ*mchC* mutant strain is as virulent as a wild-type strain in a leucopenic murine model of IPA (Fig. 7). However, it is very likely that deficient metalation of certain zinc-dependent proteins in the Δ*mchA*Δ*mchB*Δ*mchC* mutant is not enough to reduce its growth ability in leucopenic mice because the ability of the Δ*mchA*Δ*mchB*Δ*mchC* mutant to acquire zinc from host tissues remains intact.

Finally, given that THF biosynthesis has been proven to be an ideal target for an antifungal therapy (48, 49), we predict that fungal sensitivity to antifolates could be enhanced by interfering with the MchC function. Actually, this notion is strongly supported by the fact that cotrimoxazole inhibits growth of some fungal pathogens that lack MchC-like proteins such as *Pneumocystis jirovecii*, *Paracoccidioides brasiliensis,* and *Histoplasma capsulatum* (Table S2). Cotrimoxazole is used to treat *P. jirovecii* pneumonia (38), whereas *P. brasiliensis* and *H. capsulatum* are susceptible *in vitro* to treatment with cotrimoxazole (50, 51). Actually, cotrimoxazole is nearly as effective as itraconazole for treatment of paracoccidioidomycosis (52). Therefore, we propose that the absence of MchC-like proteins in these fungal pathogens could be the foundation of their sensitivity to cotrimoxazole. Moreover, we anticipate the notion that inhibition of THF biosynthesis could be strongly enhanced by interfering with MchC function in case THF biosynthesis consolidates in the near future as a new target for upcoming antifungal treatments.

## MATERIALS AND METHODS

### Fungal strains and culture media

*Aspergillus fumigatus* strains used in this study are listed in Table 1. Fresh conidia used as the inoculum were harvested from fungal strains grown in PDA complex medium (20 g/L potato dextrose agar, 20 g/L sucrose, 2.5 g/L MgSO_4_-7 H_2_O, 1 g/L agar and 1 mL/L Cove’s trace element solution 1,000× (0.04 g/L Na_2_B_4_O_7_-10 H_2_O, 8.0 g/L ZnSO_4_-7 H_2_O, 0.72 g/L MnSO_4_-1 H_2_O, 1.2 g/L FeSO_4_-7 H_2_O, 0.4 g/L CuSO_4_-5 H_2_O and 0.8 g/L Na_2_MoO_4_-2 H_2_O, pH adjusted to 6.5 with NaOH). Fungal strains were cultured in the following liquid zinc-limiting media: (i) AMM–Zn (10 g/L dextrose, 3 g/L NaNO_3_, 0.52 g/L MgSO_4_ - 7 H_2_O, 0.52 g/L KCl, 1.52 g/L KH_2_PO_4_, and 1.0 mL/L of the Cove’s trace-element solution without ZnSO_4_ and pH adjusted to 6.5 with NaOH); (ii) RPMI–Zn (i.e., RPMI-1640 [Sigma, R8755] supplemented with 10 μM FeSO_4_, 1 μM CuSO_4,_ and 1 μM MnSO_4_); (iii) SDA–Zn (pH ~4.5) (1.7 g/L YNB without amino acids, without ammonium sulfate, and without zinc [CYN2401, Formedium], 20 g/L dextrose, 5 g/L (NH_4_)_2_SO_4_, 6 µM FeSO_4_ -7 H_2_O, 2 µM CuSO_4_-5 H_2_O and 2 µM Na_2_MoO_4_-2 H_2_O) and, (iv) SDN–Zn (pH ~7.5) (it has the same composition as SDA–Zn, but ammonium sulfate is replaced by 3 g/L NaNO_3_). The fungal strains were also cultured onto the solid AMME–Zn, SDAE–Zn, and SDNE–Zn zinc-limiting media, which were prepared by supplementing the liquid AMM, SDA, and SDN zinc-limiting media with 0.25 mM EDTA and 15 g/L agar (for AMME) or 20 g/L agar (for SDAE and SDNE).

*Saccharomyces cerevisiae* strains used in this study are listed in Table S1. Yeast strains were routinely grown in yeast extract-peptone-dextrose complex medium (YEPD) at 28°C. For specific experiments, yeast strains were grown onto agar plates of the SDA–Zn (pH ~4.5) medium supplemented with EDTA plus a drop-out complete supplement mixture without uracil (SDAE+CSM–URA–Zn) (1.7 g/L YNB without amino acids, without ammonium sulfate and without zinc [CYN2401, Formedium], 0.77 g/L CSM–URA [DCS0161, Formedium], 20 g/L dextrose, 5 g/L (NH_4_)_2_SO_4_, and 0.2 mM EDTA).

To prevent or reduce contamination of culture media by traces of Zn^2+^ present in dextrose, nitrogen sources, salts, and water, ultrapure compounds of the highest quality were used and dissolved in ultrapure water. Liquid cultures were carried out in ultraclean flasks that had been treated overnight with a 3 mM EDTA (pH 8.0) solution, thoroughly rinsed with ultrapure water, and sterilized by dry heat. All solid media were prepared by adding agar to the salt solution before sterilization by autoclaving, whereas the dextrose solution used to make up the media was autoclaved separately from the other components. Culture media were supplemented with zinc, as specified using a sterile stock solution of 1 mM ZnSO_4_-7H_2_O in ultrapure water.

### Chemical compounds

Culture media were supplemented as specified using stock solutions of the following chemical compounds: sulfamethoxazole (SMX) (Sigma, S7507) in DMSO or 100% ethanol (stock at 20 mg/mL); 4-aminosalicylic acid (PAS) (Sigma, A79604) in 80% ethanol (stock 45 mg/mL); trimethoprim (TMP) (Sigma, 92131) in DMSO (stock 20 mg/mL); 4-aminobenzoic acid (PABA) (Sigma, A9878) in water (stock 1 mg/mL); TNP-470 (Sigma, T1455) in DMSO (stock 20 mg/mL); 2-(4′-chlorophenylsulfonamido) benzoic acid (CPS-BA) (Merck, ENA018104415) in DMSO (stock 20 mg/mL).

### Standard molecular biology procedures and RT-qPCR measurements

DNA manipulations were performed following standard molecular biology protocols (53). Oligonucleotides used throughout this work are listed in Table S3. All DNA fragments obtained by PCR were cloned into the pGEM-T-easy plasmid (Promega, A1360). Plasmids constructed and used for transformation of *A. fumigatus* are listed and described in Table S4.

Genomic DNA from *A. fumigatus* was obtained and analyzed by Southern blot using DIG-labeled probes, as described previously (54). The DNA fragments used as probes for Southern blot analyses were obtained by PCR using as primers and templates, respectively, the pair of oligonucleotides and plasmids shown in Table S5.

RNA was obtained and analyzed by Northern blot using radiolabeled DNA probes, as described previously (55). The DNA fragments used as probes for Northern blot analyses were obtained by PCR using as primers and templates, respectively, the pair of oligonucleotides and plasmids shown in Table S6.

For RT-qPCR measurements, the RNA samples were treated, reversed-transcribed into cDNA, and analyzed by quantitative real-time PCR (qPCR) exactly as described previously (55). Primers used for qPCR are listed in Table S3. The relative expression level with respect to 18S rRNA (REL/18S) was calculated by the 2^–∆Ct^ method. The relative expression ratio (rER) was calculated by the 2^–∆∆Ct^ method using the expression level of the 18S rRNA as the internal reference.

### Generation of *Saccharomyces cerevisiae* mutant strains

Plasmids constructed and used for transformation of *S. cerevisiae* are listed and described in Table S7. To delete the *ZNG1* gene, the yeast strains DY1457 and ZHY6 were transformed with the pZCH3 plasmid digested with the BglII/SpeI restriction enzymes according to the LiAc procedure (56), and the Δ*zng1* (CSS1) and Δ*zng1*Δ*zap1* (CSS2) mutant strains were selected onto agar plates of the YEPD medium supplemented with 200 µg/mL geneticin (G418) and 0,2 mM Zn^2+^. The DY1457 and ZHY6 yeast strains were also transformed with plasmid pRS416 to generate, respectively, the ASF0 and ASF11 strains that were used as controls. The CSS1 and CSS2 yeast strains were transformed with the pRS416 derivative plasmids pZCH13, pMCH113, pMCH210, and pMCH312 to generate, respectively, the CSS11-13 and CSS21-24 strains. All yeast strains transformed with pRS416 and pRS416 derivative plasmids were selected on the appropriate SDA dropout medium supplemented with 0.2 mM Zn^2+^.

### Generation of protoplasts and transformation of *A. fumigatus*

To obtain protoplasts of any PyrG^–^ strain, we inoculated 5 × 10^8^ conidia in fresh SDN medium supplemented with 0.05% (wt/vol) uracil and 0.12% (wt/vol) uridine and 20% (vol/vol) of a sterile conditioned SDN medium containing a highly active α-glucanase. The culture was incubated at 37°C for 14 hours. Germlings were collected by filtration through a cell strainer unit (40 µm), suspended in 10 mL of protoplasting buffer (0.2 g VinoTaste, 0.75 M KCl, 25 mM citrate/phosphate; pH 5.8), sterilized by filtration, and incubated at 35°C with shaking at 120 rpm for 2 hours. The protoplasting suspension was filtered through a miracloth (22–25 µm) (475855, Calbiochem) and centrifuged at 1,200 × *g* for 10 minutes a 4°C. The protoplast pellet was washed in 15 mL of a cold KC solution (0.6 M KCl, 50 mM CaCl_2_, pH 6.0–6.5) by mixing gently and centrifuged at 1,200 × *g* for 10 minutes at 4°C. The protoplast pellet was suspended into 0.4 mL of KC solution and used for transformation, as described previously (30). To obtain the Δ*mchA* null strain, CEA17 PyrG^–^ protoplasts were transformed with plasmid pMCH106D digested with the PmlI/NdeI restriction enzymes. To obtain the Δ*mchB* null strain, CEA17 PyrG^–^ protoplasts were transformed with plasmid pMCH205D digested with the XbaI/HpaI restriction enzymes. To obtain the Δ*mchC* null strain, CEA17 PyrG^–^ protoplasts were transformed with plasmid pMCH3091D digested with the NheI/SpeI restriction enzymes. Plates were incubated at 37°C until PyrG^+^ fungal transformants had grown. About 20–30 independent transformants were isolated and re-isolated on AMM agar plates. To identify the transformants that most likely had undergone homologous recombination at the expected locus, a sample of conidia, scrapped using a cotton swab from a colony of each isolate, was inoculated into 1 mL of AMM and incubated overnight at 37°C to obtain minipreps of genomic DNA suitable for PCR analysis using appropriate pairs of oligonucleotides. Finally, samples of genomic DNA from about 3–5 independent transformants pre-selected by PCR were digested with at least two different combinations of restriction enzymes and analyzed by Southern blot using appropriate DIG-labeled probes (Table S5, Fig. S2). Spontaneous PyrG*^−^* fungal strains were selected on AMMUF agar plates, i.e., AMM plates supplemented with 0.05% (wt/vol) uracil, 0.12% (wt/vol) uridine, and 1.0 mg/mL 5-fluoroorotic acid (5′-FOA).

The Δ*mchA*Δ*mchB* (AF131) mutant strain was generated as the Δ*mchB* null strain, but transforming the uridine-uracil-auxotrophic PyrG^–^Δ*mchA* AF127 strain instead of CEA17. The Δ*mchA*Δ*mchC* (AF124) mutant strain was generated as the Δ*mchA* null strain, and the Δ*mchB*Δ*mchC* (AF119) mutant strain was generated as the Δ*mchB* null strain, but transforming the uridine-uracil-auxotrophic PyrG^–^Δ*mchC* AF115 strain instead of CEA17. The uridine-uracil-auxotrophic PyrG^–^Δ*mchA*Δ*mchC* (AF133) strain was selected on AMMUF agar plates inoculated with the AF124 mutant strain. The Δ*mchA*Δ*mchB*Δ*mchC* (AF136) mutant strain was generated as the Δ*mchB* AF117 strain, but transforming the uridine-uracil-auxotrophic PyrG^–^ AF133 strain instead of CEA17. The AF136 mutant strain was cultured onto AMMUF agar plates to select the uridine-uracil-auxotrophic PyrG^–^Δ*mchA*Δ*mchB*Δ*mchC* (AF145) strain. This strain was transformed with a 1.15 kb DNA fragment excised from plasmid pPYRG0 digested with BamHI/XhoI to generate the Δ*mchA*Δ*mchB*Δ*mchC* (AF147) strain, which is a uridine-uracil-prototrophic, *pyrG* isogenic to the CEA10 strain.

### ROS measurement

To ascertain whether the Mch proteins were involved in promoting ROS production during zinc deficiency, we determined the total amount of intracellular ROS produced by every Δ*mch* mutant strain grown under both zinc-replete and zinc-limiting conditions using the nonfluorescent lipophilic DCFH_2_-DA as a probe. It is known that DCFH_2_-DA diffuses through the cell membrane and enters into the cells where it is deacetylated by intracellular esterases to form DCFH_2_, which is a membrane-impermeable nonfluorescent molecule that reacts with intracellular ROS to give the yellow DCF compound that shows intense fluorescence upon excitation at 485–500 nm (46). Fungal strains were inoculated (5 × 10^5^ conidia/mL) in 50 mL of the AMM–Zn zinc-limiting medium or in 20 mL of this medium supplemented with 100 µM zinc and incubated at 37°C for 22 h with shaking at 200 rpm. The DCFH_2_-DA probe (Sigma, D6883) was added at a final concentration of 10 µM (using a stock 10 mM in pure ethanol stored at –20°C), and cultures were incubated for 2.5 h longer. The mycelia were harvested by filtration on filter paper, washed three times with sterile PBS, and vacuum-dried. Mycelia samples were disposed of in 1.5 mL microtubes and were added a volume of 0,5 mL of glass beads (425–600 μm, Sigma, G8772) and 0.5 mL PBS. Mycelia was lysed in a FastPrep machine (3 × 23 s, power 5.5 at 4°C, MP biomedicals). The bottom of each tube was perforated using a hot needle, disposed within a new tube and centrifuged at 2,000 rpm for 30 s at room temperature. Lysates were clarified by centrifugation at 16,000 × *g* for 10 minutes a 4°C and transferred to clean tubes. Aliquots of 0.1 mL were dispensed by triplicate for each strain and culture condition in a 96-well flat-bottom black microplate. Samples were excited at 485 nm, and fluorescence emitted at 530 nm was measured in a spectrofluorimeter (Varioskan Flash, Thermo Scientific). The amount of ROS in each sample was calculated with respect to the total amount of protein in the same sample measured using the Bradford Protein Assay (BIO-RAD, 5000205) and expressed as relative fluorescence units.

### Murine model of invasive pulmonary aspergillosis

*SPF-CD1* male mice (~25 g each) were grouped in cohorts of 10 mice. Spores were harvested from PDA plates and washed twice with a saline buffer plus 0.01% Tween-20. A cyclophosphamide-/cortisone-based immunosuppressive regimen was used, which causes a depletion of circulating white blood cells (i.e., a leukopenic regime). Mice were immunosuppressed on days –3 and –1 before inoculation with cyclophosphamide (150 mg/kg) and cortisone 21-acetate (112 mg/kg). Afterward, only cyclophosphamide (150 mg/kg) was administered on days +3, +6, +9, and +12 after inoculation. Before infection, each mouse was anesthetized with 0.1 mL of a mix of ketamine plus xylazine. Conidia of the Δ*mchA*Δ*mchB*Δ*mchC* mutant strain were used to inoculate two different cohorts of leucopenic mice; mice of one cohort were inoculated with 10^5^ conidia per mouse (c/m), whereas mice of the other cohort were inoculated with 10^4^ c/m. Each mouse was inoculated intranasally with 30 µL of a suspension with 6.5 × 10^6^ conidia/mL or 6.5 × 10^5^ conidia/mL to ensure that during the instillation process, each mouse was inoculated with no less than 10^5^ c/m or 10^4^ c/m. Non-inoculated control immunosuppressed mice only received saline buffer. Survival of mice in both cohorts was followed for 15 days. Mice were euthanized to avoid suffering when they developed symptoms of severe respiratory distress.

### Statistical analyses

Fungal growth and ROS production were analyzed by using a non-paired, two-tailed *t*-test with 95% confidence intervals using Prism 7.0 software (GraphPad Software). Survival of mice was analyzed by the Mantel-Cox log-rank test using Prism 7.0 software. A *P* value of < 0.05 was considered statistically significant.

## SUPPLEMENTAL MATERIAL

Fig. S1 spectrum.02279-25-s0001.pdf Distinctive structural features and phylogeny of the Mch proteins of *Aspergillus fumigatus*.

Supplemental figures spectrum.02279-25-s0002.pdf Fig. S2 to S11.

Supplemental material spectrum.02279-25-s0003.pdf Supplemental text; Tables S1 to S7.

ASM does not own the copyrights to Supplemental Material that may be linked to, or accessed through, an article. The authors have granted ASM a non-exclusive, world-wide license to publish the Supplemental Material files. Please contact the corresponding author directly for reuse.
