# Supplementary material for: Zinc-metallochaperones of Aspergillus fumigatus are involved in ROS production and folate biosynthesis during zinc deficiency
Source: Microbiol Spectr. 2025 Sep 23;13(11):e02279-25. doi: 10.1128/spectrum.02279-25 (PMC12584730; doi:10.1128/spectrum.02279-25)
Supplement: Fig. S1 — Distinctive structural features and phylogeny of the Mch proteins of Aspergillus fumigatus. [file spectrum.02279-25-s0001.pdf]

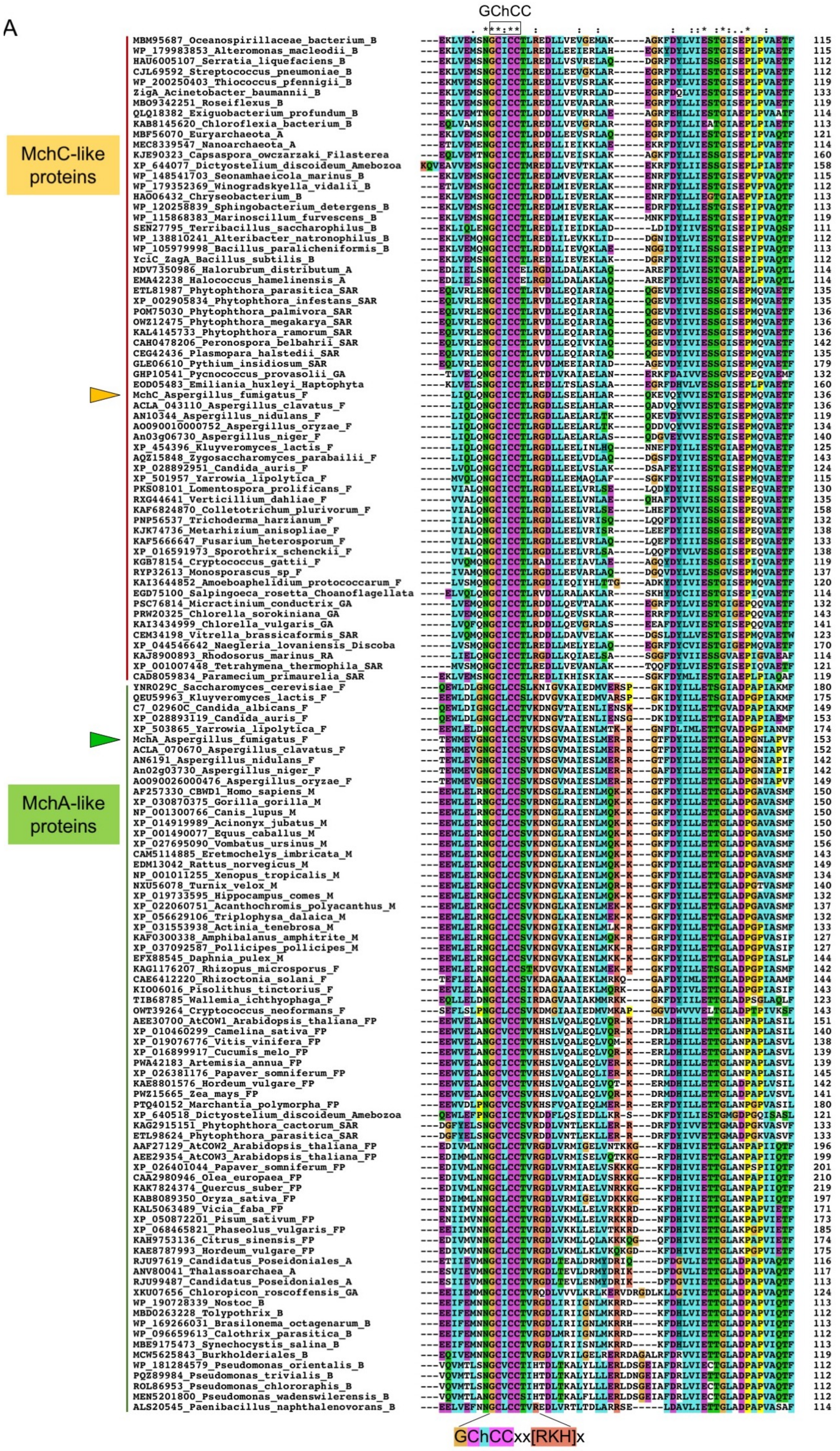
A
GChCC
MchC-like proteins
MchA-like proteins
GChCCxx[RKH]x

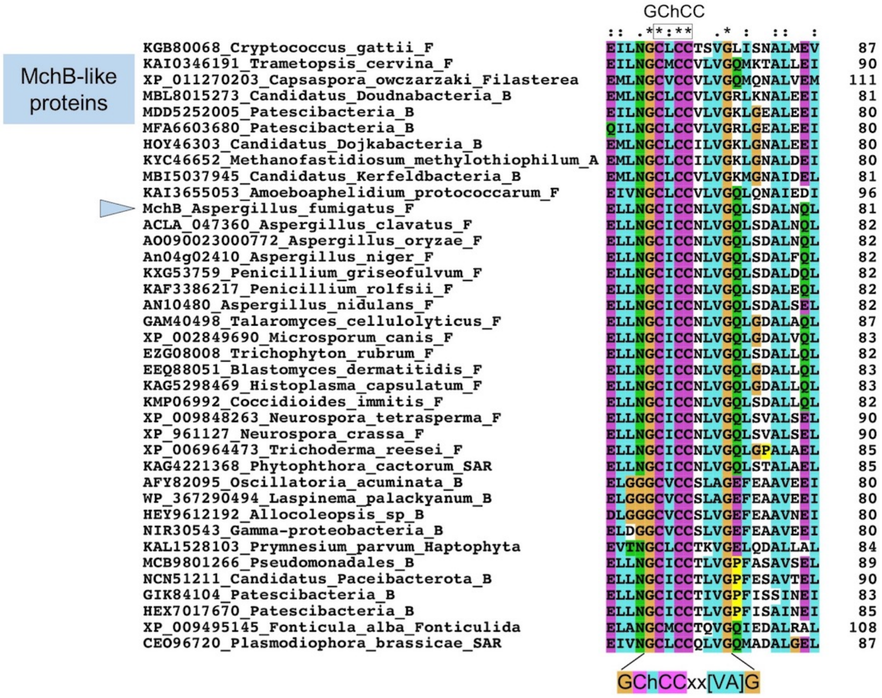

Figure S1. Distinctive structural features and phylogeny of the Mch proteins of *Aspergillus fumigatus*. (A) Conserved sequences downstream of the GChCC motif present in the Mch proteins of *A. fumigatus* and in all other COG0523 proteins used in this analysis, including 70 MchA-like proteins, 38 MchB-like proteins and 62 MchC-like proteins from different prokaryotic (A, Archaea; B, bacteria) and eukaryotic organisms (F, fungi; SAR, protist of the Stramenopila-Alveolata-Rhizaria supergroup; GA, green algae; RA, red algae; FP, flowering plants; M, metazoan). The MchA, MchB and MchC proteins of *A. fumigatus* have been indicated respectively with green, blue and orange arrowheads. These conserved sequences are useful as signature sequences to differentiate the MchA- and MchC-like proteins (both carrying the GChCCxx[RKH]x motif) from the MchB-like proteins (carrying the GChCCxx[VA]G motif).

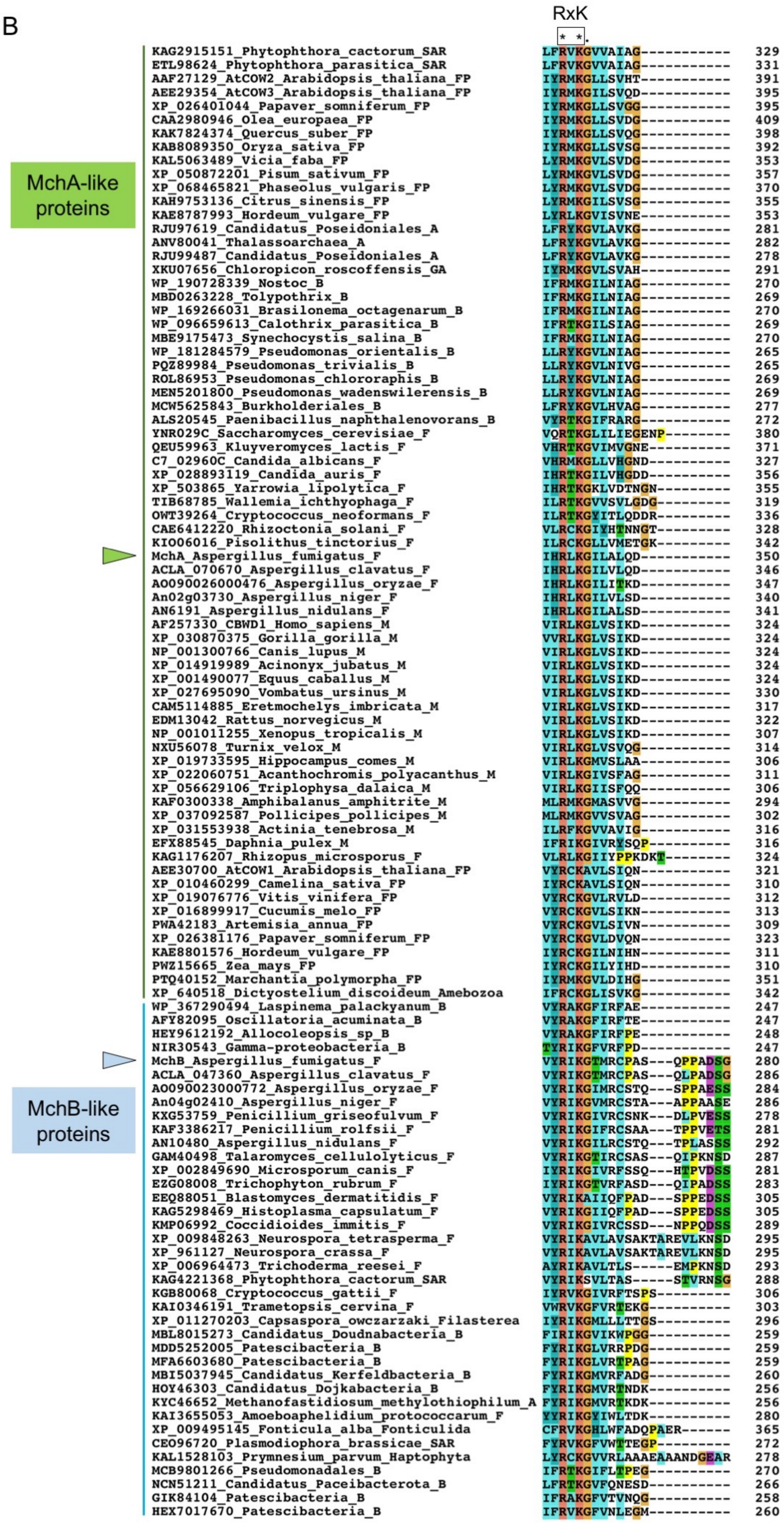

B
RxK
MchA-like proteins
MchB-like proteins
KAG2915151_Phytophthora_cactorum_SAR LFRVKGVVAIAG----------- 329
ETL98624_Phytophthora_parasitica_SAR LFRVKGVVAIAG----------- 331
AAF27129_AtCOW2_Arabidopsis_thaliana_FP IYRMKGLLSVHT----------- 391
AEE29354_AtCOW3_Arabidopsis_thaliana_FP IYRMKGLLSVQD----------- 395
XP_026401044_Papaver_somniferum_FP IYRMKGLLSVGG----------- 395
CAA2980946_Olea_europaea_FP IYRMKGLLSVDG----------- 409
KAK7824374_Quercus_suber_FP IYRMKGLLSVQG----------- 398
KAB8089350_Oryza_sativa_FP IYRMKGLLSVSG----------- 392
KAL5063489_Vicia_faba_FP LYRMKGVLSVDG----------- 353
XP_050872201_Pisum_sativum_FP LYRMKGVLSVDG----------- 357
XP_068465821_Phaseolus_vulgaris_FP LYRMKGVLSVDG----------- 370
KAH9753136_Citrus_sinensis_FP LYRMKGILSVSG----------- 355
KAE8787993_Hordeum_vulgare_FP LYRLKGVISVNE----------- 353
RJU97619_Candidatus_Poseidoniales_A LFRYKGVLAVKG----------- 281
ANV80041_Thalassoarchaea_A LFRYKGVLAVKG----------- 282
RJU99487_Candidatus_Poseidoniales_A LFRYKGVLAVKG----------- 278
XKU07656_Chloropicon_roscoffensis_GA IYRMKGVLSVAH----------- 291
WP_190728339_Nostoc_B IFRMKGILNIAG----------- 270
MBD0263228_Tolypothrix_B IFRMKGILNIAG----------- 269
WP_169266031_Brasilonema_octagenarum_B IFRMKGILNIAG----------- 270
WP_096659613_Calothrix_parasitica_B IFRTKGILSIAG----------- 269
MBE9175473_Synechocystis_salina_B IFRMKGILNIAG----------- 270
WP_181284579_Pseudomonas_orientalis_B LLRYKGVLNIAG----------- 265
PQZ89984_Pseudomonas_trivialis_B LLRYKGVLNIVG----------- 265
ROL86953_Pseudomonas_chlororaphis_B LLRYKGVLNIAG----------- 269
MEN5201800_Pseudomonas_wadenswilerensis_B LLRYKGVLNIAG----------- 269
MCW5625843_Burkholderiales_B LFRYKGVLHVAG----------- 277
ALS20545_Paenibacillus_naphthalenovorans_B VYRTKGIFRARG----------- 272
YNR029C_Saccharomyces_cerevisiae_F VQRTKGLILIEGENP-------- 380
QEU59963_Kluyveromyces_lactis_F VHRTKGVIMVGNE---------- 371
C7_02960C_Candida_albicans_F VHRMKGLLVHGND---------- 327
XP_028893119_Candida_auris_F IHRTKGILVHGDD---------- 356
XP_503865_Yarrowia_lipolytica_F IHRTKGKLVDTNGN--------- 355
TIB68785_Wallemia_ichthyophaga_F ILRTKGVVSVLGDG--------- 319
OWT39264_Cryptococcus_neoformans_F ILRTKGYITLQDDR--------- 336
CAE6412220_Rhizoctonia_solani_F VLRCKGIYHTNNGT--------- 328
KIO06016_Pisolithus_tinctorius_F ILRCKGLLVMETGK--------- 342
MchA_Aspergillus_fumigatus_F IHRLKGILALQD----------- 350
ACLA_070670_Aspergillus_clavatus_F IHRLKGILVLQD----------- 346
AO090026000476_Aspergillus_oryzae_F IHRLKGILITKD----------- 347
An02g03730_Aspergillus_niger_F IHRLKGILVLSD----------- 340
AN6191_Aspergillus_nidulans_F IHRLKGILALSD----------- 341
AF257330_CBWD1_Homo_sapiens_M VIRLKGLVSIKD----------- 324
XP_030870375_Gorilla_gorilla_M VVRLKGLVSIKD----------- 324
NP_001300766_Canis_lupus_M VIRLKGLVSIKD----------- 324
XP_014919989_Acinonyx_jubatus_M VIRLKGLVSIKD----------- 324
XP_001490077_Equus_caballus_M VIRLKGLVSIKD----------- 324
XP_027695090_Vombatus_ursinus_M VIRLKGLVSIKD----------- 330
CAM5114885_Eretmochelys_imbricata_M VIRLKGLVSIKD----------- 317
EDM13042_Rattus_norvegicus_M VIRLKGLVSIKD----------- 322
NP_001011255_Xenopus_tropicalis_M VIRLKGLVSIKD----------- 307
NXU56078_Turnix_velox_M VIRLKGLVSVQG----------- 314
XP_019733595_Hippocampus_comes_M VIRLKGMVSLAA----------- 306
XP_022060751_Acanthochromis_polyacanthus_M VIRLKGIVSFAG----------- 311
XP_056629106_Triplophysa_dalaica_M VIRLKGIISFQQ----------- 306
KAF0300338_Amphibalanus_amphitrite_M MLRMKGMASVVG----------- 294
XP_037092587_Pollicipes_pollicipes_M MLRMKGVVSVAG----------- 302
XP_031553938_Actinia_tenebrosa_M ILRFKGVVAVIG----------- 316
EFX88545_Daphnia_pulex_M IFRIKGIVRYSQP---------- 316
KAG1176207_Rhizopus_microsporus_F VLRLKGIIYPPKDKT-------- 324
AEE30700_AtCOW1_Arabidopsis_thaliana_FP VYRCKAVLSIQN----------- 321
XP_010460299_Camelina_sativa_FP IYRCKAVLSIQN----------- 310
XP_019076776_Vitis_vinifera_FP VYRCKGVLRVLD----------- 312
XP_016899917_Cucumis_melo_FP VYRCKGVLSIKN----------- 313
PWA42183_Artemisia_annua_FP VYRCKGVLSIVN----------- 309
XP_026381176_Papaver_somniferum_FP VYRCKGVLDVQN----------- 323
KAE8801576_Hordeum_vulgare_FP IYRCKGILNIHN----------- 311
PWZ15665_Zea_mays_FP IYRCKGILYIHD----------- 310
PTQ40152_Marchantia_polymorpha_FP IYRMKGVLDIHG----------- 351
XP_640518_Dictyostelium_discoideum_Amebozoa IFRCKGLISVKG----------- 342
WP_367290494_Laspinema_palackyanum_B VYRAKGFIRFAE----------- 247
AFY82095_Oscillatoria_acuminata_B VYRAKGFIRFTE----------- 247
HEY9612192_Allocoleopsis_sp_B VYRAKGFIRFPE----------- 248
NIR30543_Gamma-proteobacteria_B TYRIKGFVRFPD----------- 247
MchB_Aspergillus_fumigatus_F VYRIKGTMRCPAS---QPPADSG 280
ACLA_047360_Aspergillus_clavatus_F VYRIKGTMRCPAS---QLPADSG 286
AO090023000772_Aspergillus_oryzae_F VYRIKGIMRCSTQ---SPPAESS 284
An04g02410_Aspergillus_niger_F VYRIKGVMRCSTA---APPAASE 286
KXG53759_Penicillium_griseofulvum_F VYRIKGIVRCSNK---DLPVESS 278
KAF3386217_Penicillium_rolfsii_F VYRIKGIFRCSAA---TPPVETS 281
AN10480_Aspergillus_nidulans_F VYRIKGILRCSTQ---TPLASSS 292
GAM40498_Talaromyces_cellulolyticus_F VYRIKGTIRCSAS---QIPKNSD 287
XP_002849690_Microsporum_canis_F IYRIKGIVRFSSQ---HTPVDSS 281
EZG08008_Trichophyton_rubrum_F IYRIKGTVRFASD---QIPADSS 283
EEQ88051_Blastomyces_dermatitidis_F VYRIKAIIQFPAD---SPPEDSS 305
KAG5298469_Histoplasma_capsulatum_F VYRIKGIIQFPAD---SPPEDSS 305
KMP06992_Coccidioides_immitis_F VYRIKGIVRCSSD---NPPQDSS 289
XP_009848263_Neurospora_tetrasperma_F VYRIKAVLAVSAKTAREVLKNSD 295
XP_961127_Neurospora_crassa_F VYRIKAVLAVSAKTAREVLKNSD 295
XP_006964473_Trichoderma_reesei_F AYRIKAVLTLS-----EMPKNSD 293
KAG4221368_Phytophthora_cactorum_SAR VYRIKSVLTAS-----STVRNSG 288
KGB80068_Cryptococcus_gattii_F IYRVKGIVRFTSPS--------- 306
KAI0346191_Trametopsis_cervina_F VWRVKGFVRTEKG---------- 303
XP_011270203_Capsaspora_owczarzaki_Filasterea IYRIKGMLLLTTGS--------- 296
MBL8015273_Candidatus_Doudnabacteria_B FIRIKGVIKWPGG---------- 259
MDD5252005_Patescibacteria_B FYRIKGLVRRPDG---------- 259
MFA6603680_Patescibacteria_B FYRIKGLVRTPAG---------- 259
MBI5037945_Candidatus_Kerfeldbacteria_B FYRIKGMVRFADG---------- 260
HOY46303_Candidatus_Dojkabacteria_B FYRIKGMVRTNDK---------- 256
KYC46652_Methanofastidiosum_methylothiophilum_A FYRIKGMVRTKDK---------- 256
KAI3655053_Amoeboaphelidium_protococcarum_F YYRIKGYIWLTDK---------- 280
XP_009495145_Fonticula_alba_Fonticulida CFRVKGHLWFADQPAER------ 365
CEO96720_Plasmodiophora_brassicae_SAR FYRVKGFVWTTEGP--------- 272
KAL1528103_Prymnesium_parvum_Haptophyta LYRCKGVVRLAAAEAAANDGEAR 278
MCB9801266_Pseudomonadales_B IFRTKGIFLTPEG---------- 270
NCN51211_Candidatus_Paceibacterota_B LFRTKGVFQNESD---------- 266
GIK84104_Patescibacteria_B IFRAKGFVTVNQG---------- 258
HEX7017670_Patescibacteria_B IFRVKGFVNLEGM---------- 260

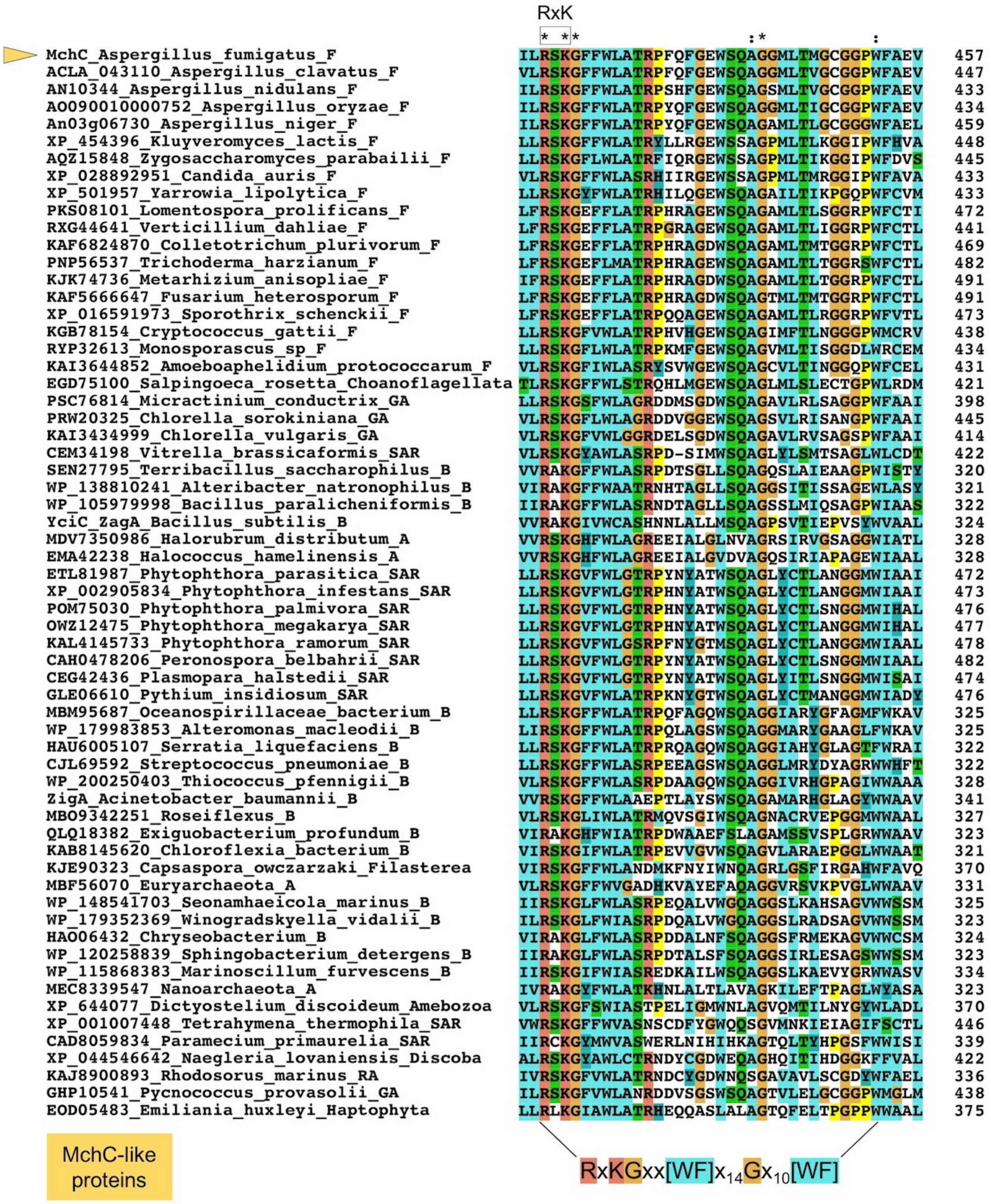

Figure S1. Distinctive structural features and phylogeny of the Mch proteins of *Aspergillus fumigatus*. (B) Conserved RxK motif in the Mch-like proteins. The MchA, MchB and MchC proteins of *A. fumigatus* have been indicated respectively with green, blue and orange arrowheads. The conserved sequence motif RxKGxx[WF]x$_{14}$Gx$_{10}$[WF] located downstream of the RxK motif is only present in the MchC-like proteins and is useful as a signature sequence to differentiate MchC-like proteins from the MchA- and MchB-like proteins.

C

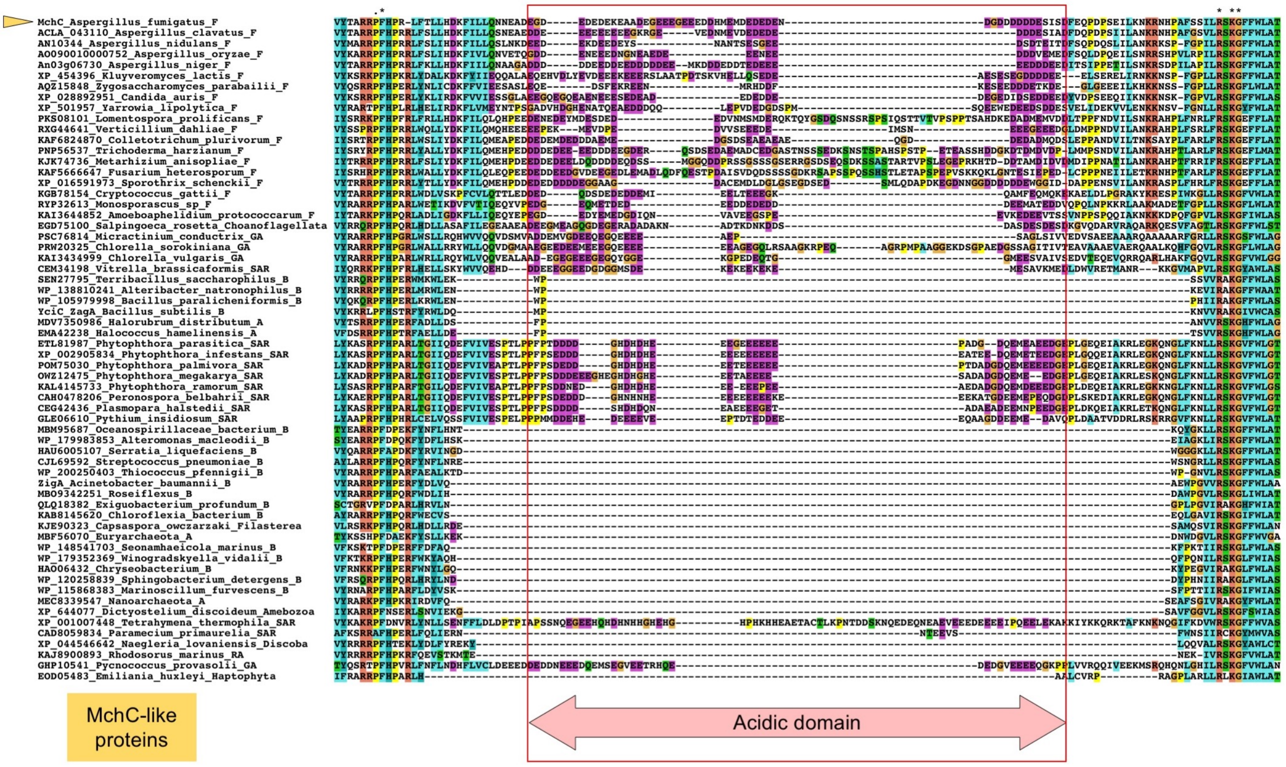

D

```
MchB_A_luchuensis      194 VLLGIDGALFGE---EGVDGVDGHGHSHGDGEGHKHEHGHQSEVEVL
MchB_A_niger           194 VLLGIDGALFGE---EGVDGHE-HGHEHNHGEGHKHEHGHQSEVEVL
MchB_A_sydowii         201 VLLGIDGALFAKGGEEDLDSGKVDGHDHGHG--HGHEHGHQSEVEVL
MchB_A_versicolor      201 VLLGIDGALFAKGGEEDLDSGKVDGHDHGHGHEHGHEHGHQSEVEVL
MchB_A_nidulans        200 VLLGIDGALFAK---QVIENGHEHGHEHADG--HGHEHGHQSEVEVL
MchB_A_carbonarius     196 VLLGIDGALVAK---EGGVELLGEGHGHGEN-GHEHQHGHQSEVEVL
MchB_A_fumigatus       193 VLLGIDGALFAKEDA---EDALRLTDDH-----HQHRHDHQSEVEVL
MchB_A_novofumigatus   193 VLLGLDGALFAKEDA---EDALRLTDDH-----HQHRHDHQSEVEVL
MchB_A_clavatus        194 VLLGIDGALFAKKGD---EEALTLTEHHSHEHGHEHHHDHQSEVEVL
MchB_A_ochraceoroseus  194 VLLGIDGALFAKEVD---WKSNHHHHHH--EHEHEHDHDHQSEVEVL
MchB_A_parasiticus     194 VLLGIDGALFTK--D---DGAGLTDGHHDHDHGHGHKHDHQSEVEVL
MchB_A_oryzae          194 VLLGIDGALFTK--D---DGAGLTDGHHDHDHGHGHKHDHQSEVEVL
MchB_A_terreus         194 VLLGIDGALFAKVVDGHREEEALGQHHHDHDHGDGHAH-HQSEVEVL
                           ****:****. :                *     . * * ********

MchC_A_luchuensis      540 DGWEDWPDL---EDDDIKDEEEEVK-GEKSGKRKASELLNG-HGHG--HAHGHSHAHGPGHKSRPVVKK
MchC_A_niger           542 DGWEDWPDL---EEDDIKDEEEEKKNGEKSGKRKASELMNG-HGHGHAHAHGHSHAHGQAHKTRPVVKE
MchC_A_carbonarius     545 DGWEDWPGL---DEDEIEMNEEDEG-----KKRKASELLHG-HGHG--HNH-HGHAHAQKK--------
MchC_A_terreus         535 DGWEGWVSPEYADEDEDEMQTET----ETKGKHRISEHLGHNHNHGPGHSHSHSHTHPRKKAQK-----
MchC_A_parasiticus     517 DGWEEWPS---LEEEEDEPEQE-------KRKHRISEHLGH------GHSHNHNHSH-RMGIKA----
MchC_A_oryzae          517 DGWEEWPS---LEEE-DEPEQE-------KGKHRISEHLGH------GHSHNHNHSH-RMGIKA-----
MchC_A_fumigatus       542 DGWEAWPEFGIVDEDEEDNNEGG----KKKHKHYTSEYQGR------VHSHSHK---------------
MchC_A_novofumigatus   537 DGWEAWPEFGIVDEDEEDNKEGG----KKKHKHHTSEYLGH------AHSHSHSHSHSHK---------
MchC_A_clavatus        530 DGWEAWPEFGIEDEEEEEEEEEEEKKKKKQKKHRTSEFQKH------NHSHGHVHTHK-----------
MchC_A_nidulans        516 DGWEDWPE----VEMEIEER-IWFKFILSTVTRHASEIIFV----------------------------
MchC_A_ochraceoroseus  505 DGWEDWPG----PGEEHED--------------------------------------------------
MchC_A_versicolor      520 DGWEDWPDG---EDLEI----------------------------------------------------
MchC_A_sydowii         521 DGWEDWP-----DADEVEI--------------------------------------------------
                           **** *
```

Figure S1. Distinctive structural features and phylogeny of the Mch proteins of *Aspergillus fumigatus.* **(C)** Some MchC-like proteins from eukaryotic organisms such as fungi, green algae and protists of the SAR supergroup, show an acidic domain with a high content in Asp and Glu residues located just upstream of their RxK motifs. The MchC protein of *A. fumigatus* has been indicated with an orange arrowhead. **(D)** His-rich motifs in the MchB- and MchC-like proteins from different *Aspergilli.* In the MchB proteins nearly all His residues are located about 35-42 amino acids upstream of their RxK motifs. In most MchC proteins the His-rich sequences are located in their C-terminal ends.

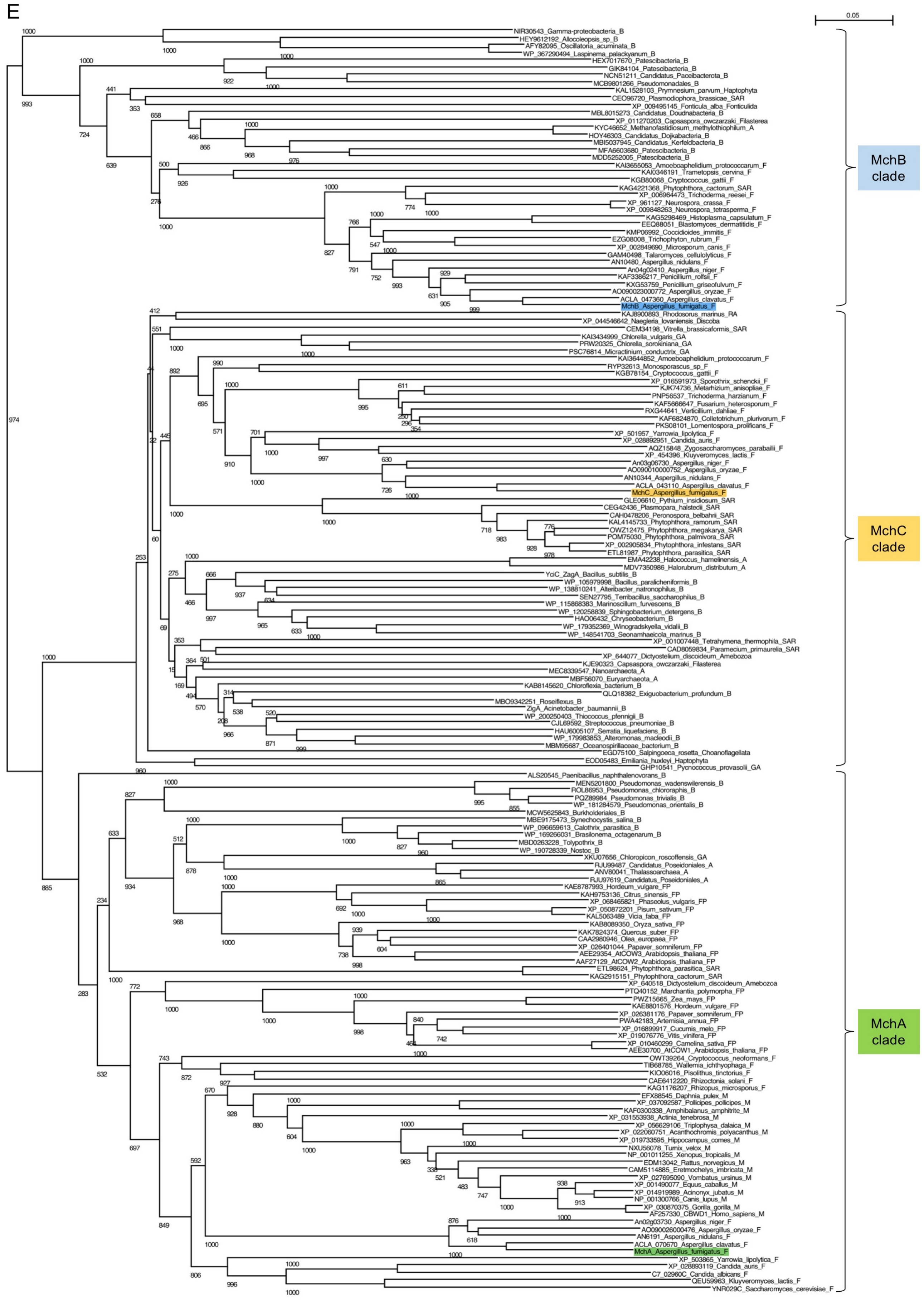

Figure S1. Distinctive structural features and phylogeny of the Mch proteins of *Aspergillus fumigatus*. (E) Phylogenetic tree constructed with Clustal X 2.0 (randon number generator seed set at 111 and number of bootstrap trials set at 1000) using the same proteins indicated in Fig. S1A. The MchA, MchB and MchC proteins of *A. fumigatus* that have been highlighted respectively in green, blue and orange.
