# Supplementary material for: Zinc-metallochaperones of Aspergillus fumigatus are involved in ROS production and folate biosynthesis during zinc deficiency
Source: Microbiol Spectr. 2025 Sep 23;13(11):e02279-25. doi: 10.1128/spectrum.02279-25 (PMC12584730; doi:10.1128/spectrum.02279-25)
Supplement: Supplemental figures — Fig. S2 to S11. [file spectrum.02279-25-s0002.pdf]

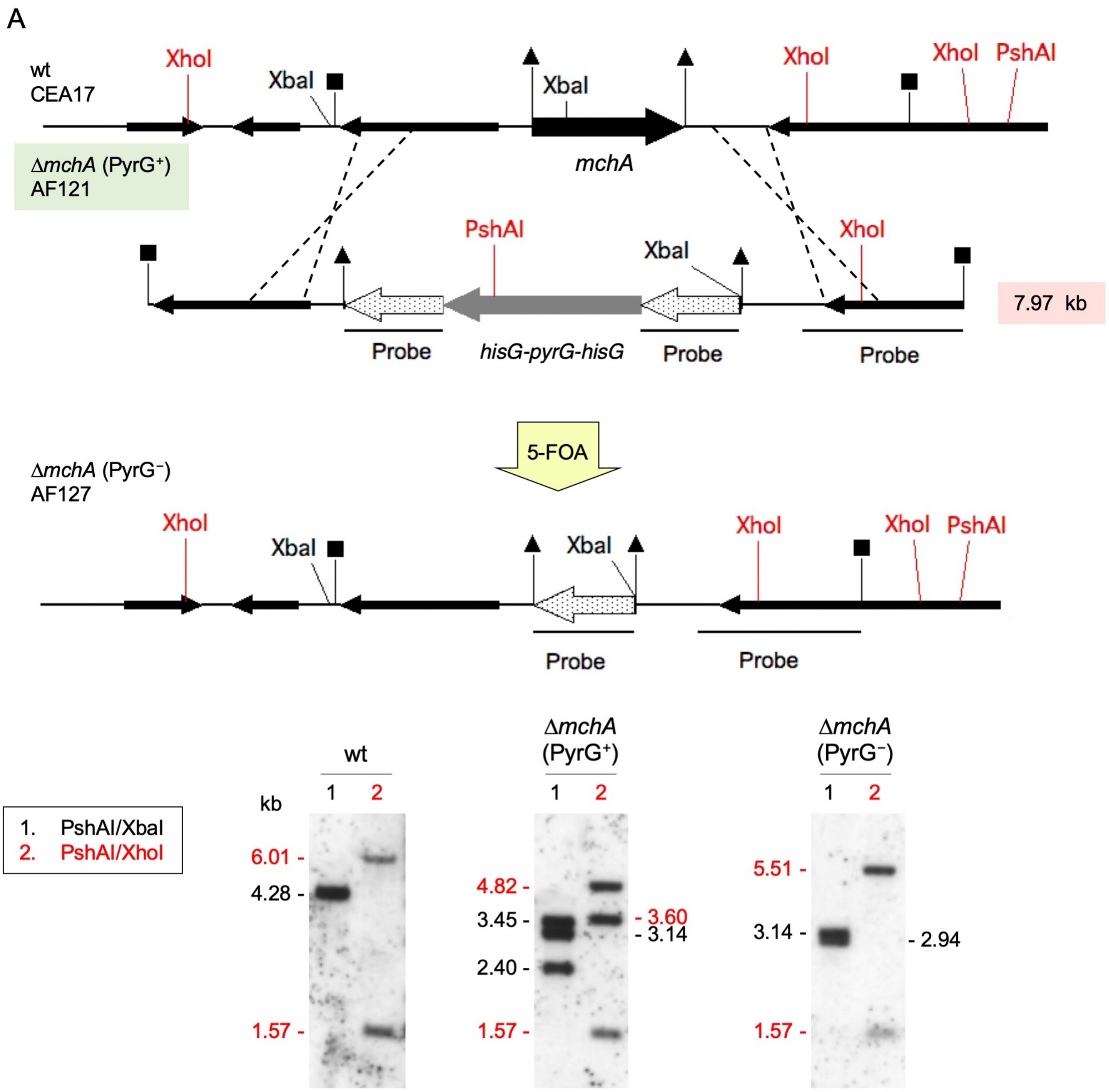

Figure S2. Construction of the Δ*mchA*, Δ*mchB* and Δ*mchC* mutant strains of *Aspergillus fumigatus*. (A) A genomic DNA fragment of the uracil-uridine auxotrophic PyrG⁻ CEA17 strain containing the complete coding sequence of *mchA* (delimited with triangles) was replaced in by the *hisG-pyrG-hisG* cassette (grey arrow flanked by doted arrows) using as transforming DNA a 7.97 kb DNA fragment, which was excised from plasmid pMCH106D by digestion with PmlI/NdeI, to generate the **Δ*mchA* AF121 strain**. The *pyrG* gene (grey arrow) of AF121 was removed by spontaneous intra-chromosomal recombination between the direct repeat *hisG* sequences that flanked the auxotrophic *pyrG* selection marker to generate the uridine-uracil-auxotrophic **PyrG⁻ Δ*mchA* AF127 strain** that was selected on AMMUF agar plates. The strains AF121 and AF127 harboured the expected mutated DNA fragments in their *mchA* loci, as verified by southern-blot using as a probe a mixture of two different DNA fragments of 1578 and 867 bp obtained by PCR using, respectively, the pairs of oligonucleotides JA469/JA470 and JA493/JA494 and the plasmids pMCH104 and pHISG as templates. DNA sequences detected by the probes are indicated by black lines. In all schemes the thinner arrows indicate putative open reading frames surrounding the *mchA* gene. Only relevant restriction sites are indicated. The source of the genomic DNA, the restriction enzymes used in the digestions, and the sizes of the fragments detected that match the expected sizes are specifically indicated in each blot. The size of fragments produced by the two different double digestions labelled as 1 and 2 in each panel are in black and red, respectively.

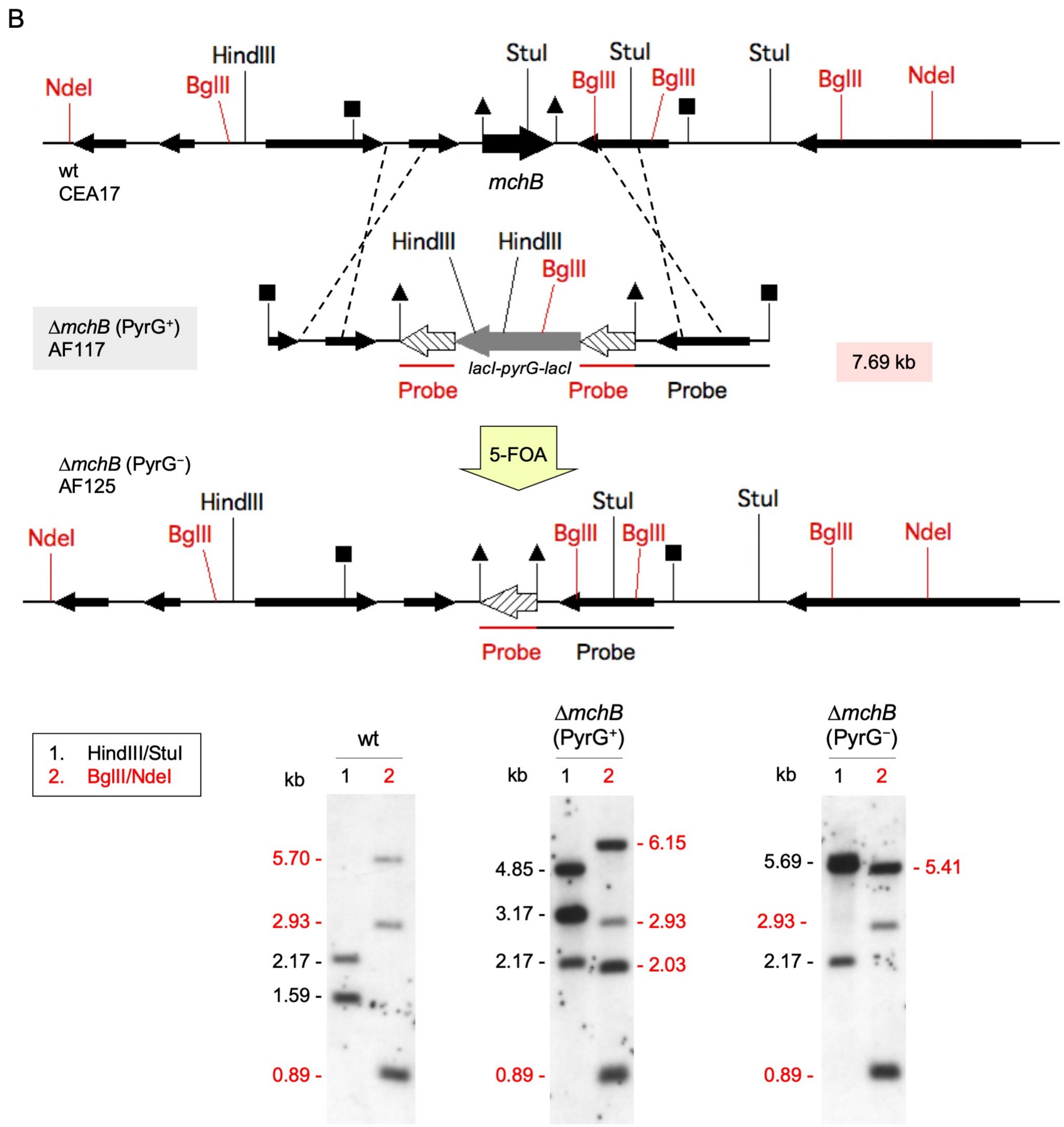

Figure S2. Construction of the Δ*mchA*, Δ*mchB* and Δ*mchC* mutant strains of *Aspergillus fumigatus*. (B) A genomic DNA fragment of the CEA17 strain containing the complete coding sequence of *mchB* (delimited with triangles) was replaced in by the *lacI-pyrG-lacI* cassette (grey arrow flanked by dashed arrows) using as transforming DNA a 7.69 kb DNA fragment, which was excised from plasmid pMCH205D by digestion with XbaI/HpaI, to generate the **Δ*mchB* AF117 strain**. The *pyrG* gene (grey arrow) of AF117 was removed by spontaneous intra-chromosomal recombination between the direct repeat *lacI* sequences that flanked the auxotrophic *pyrG* selection marker to generate the uridine-uracil-auxotrophic **PyrG^{-} Δ*mchB* AF125 strain** that was selected on AMMUF agar plates. The strains AF117 and AF125 harboured the expected mutated DNA fragments in their *mchB* loci, as verified by southern-blot using as a probe a mixture of two different DNA fragments of 2078 and 844 bp obtained by PCR using, respectively, the pairs of oligonucleotides JA474/JA475 and JA90/JA91 and the plasmids pMCH203 and pLAC as templates. DNA sequences detected by the probes are indicated by red and black lines. In all schemes the thinner arrows indicate putative open reading frames surrounding the *mchB* gene. Only relevant restriction sites are indicated. The source of the genomic DNA, the restriction enzymes used in the digestions, and the sizes of the fragments detected that match the expected sizes are specifically indicated in each blot. The size of fragments produced by the two different double digestions labelled as 1 and 2 in each panel are in black and red, respectively.

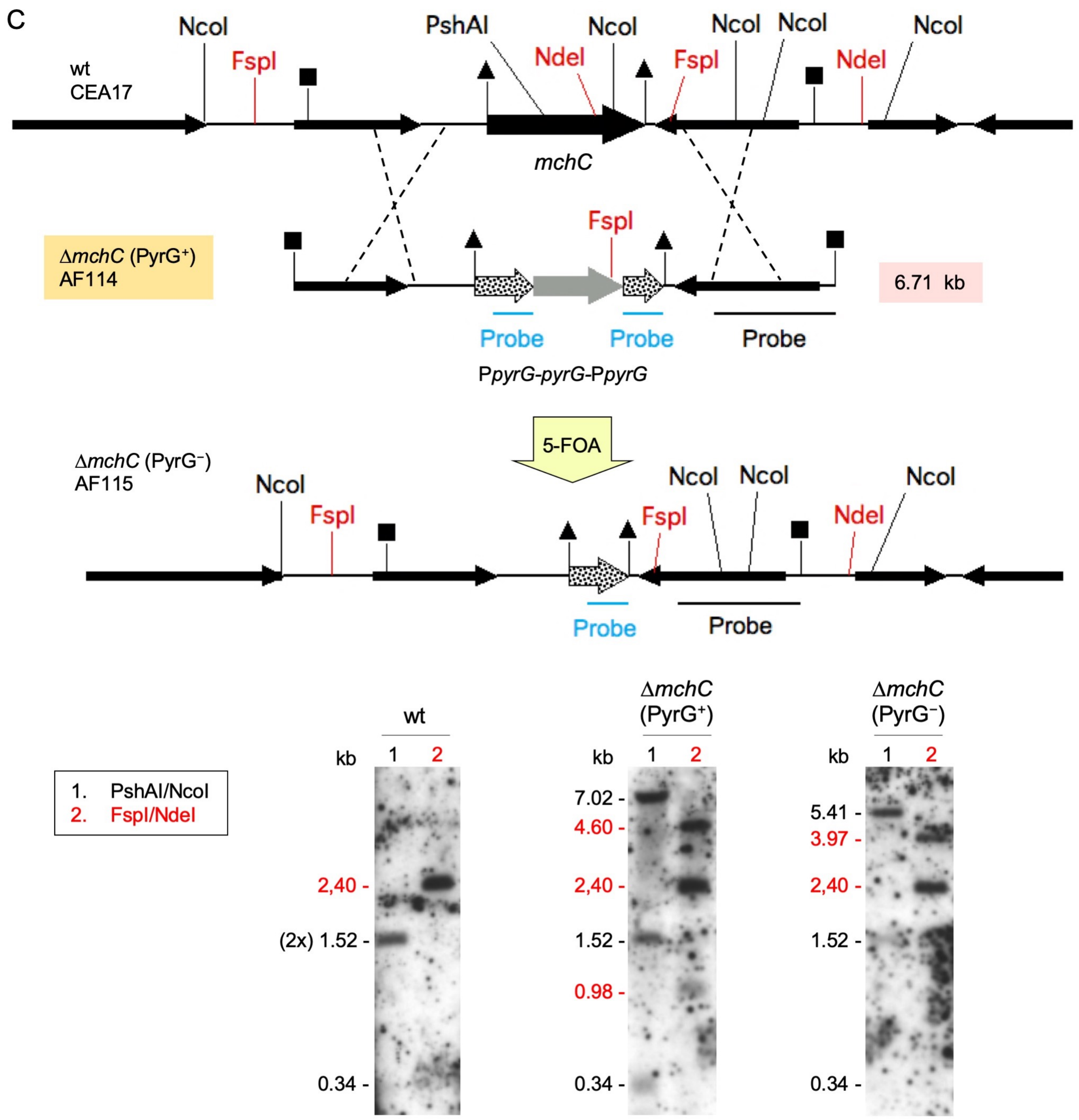

Figure S2. Construction of the Δ*mchA*, Δ*mchB* and Δ*mchC* mutant strains of *Aspergillus fumigatus*. (C) A genomic DNA fragment of the CEA17 strain containing the complete coding sequence of *mchC* (delimited with triangles) was replaced in by the *PpyrG-pyrG-PpyrG* cassette (grey arrow flanked by textured arrows) using as transforming DNA a 6.71 kb DNA fragment, which was excised from plasmid pMCH3091D by digestion with NheI/SpeI, to generate the **Δ*mchC* AF114 strain**. The *pyrG* gene (grey arrow) of AF114 was removed by spontaneous intra-chromosomal recombination between the direct repeat *PpyrG* sequences that flanked the auxotrophic *pyrG* selection marker to generate the uridine-uracil-auxotrophic **PyrG^{-} Δ*mchC* AF115 strain** that was selected on AMMUF agar plates. The strains AF114 and AF115 harboured the expected mutated DNA fragments in their *mchC* loci, as verified by Southern-blot using as a probe a mixture of two different DNA fragments of 1505 and 508 bp obtained by PCR using the pairs of oligonucleotides JA481/JA482 and JA373/JA374 and the plasmids pMCH304 and pPYRG11 as templates, respectively. DNA sequences detected by the probes are indicated by blue and black lines. In all schemes the thinner arrows indicate putative open reading frames surrounding the *mchC* gene. Only relevant restriction sites are indicated. The source of the genomic DNA, the restriction enzymes used in the digestions, and the sizes of the fragments detected that match the expected sizes are specifically indicated in each blot. The size of fragments produced by the two different double digestions labelled as 1 and 2 in each panel are in black and red, respectively.

A

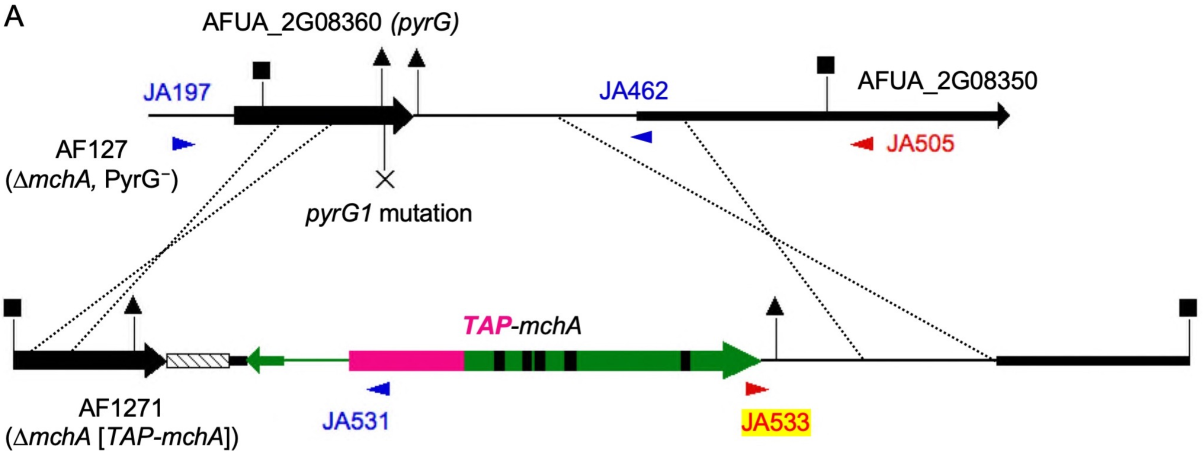

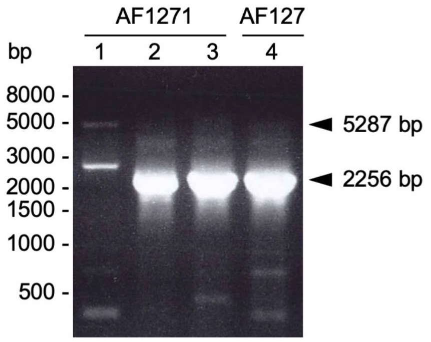

Lane	Oligonucleotides	Expected size of fragments
1	JA197/JA462	5287 bp in AF1271
2	JA197/JA531	2161 bp in AF1271
3	JA533/JA505	2285 bp in AF1271
4	JA197/JA462	2256 bp in AF127

B

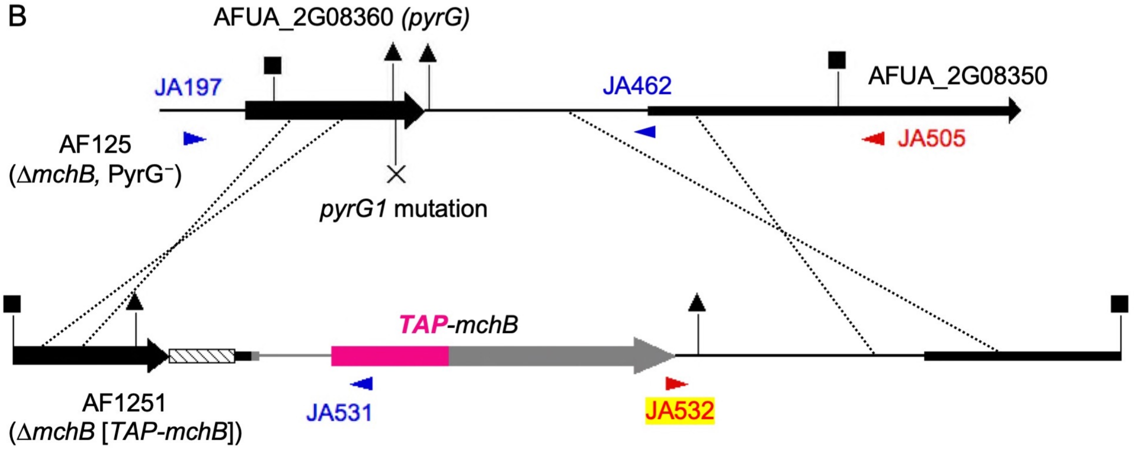

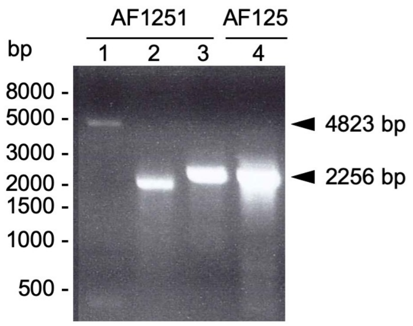

Lane	Oligonucleotides	Expected size of fragments
1	JA197/JA462	4823 bp in AF1251
2	JA197/JA531	2037 bp in AF1251
3	JA532/JA505	2289 bp in AF1251
4	JA197/JA462	2256 bp in AF125

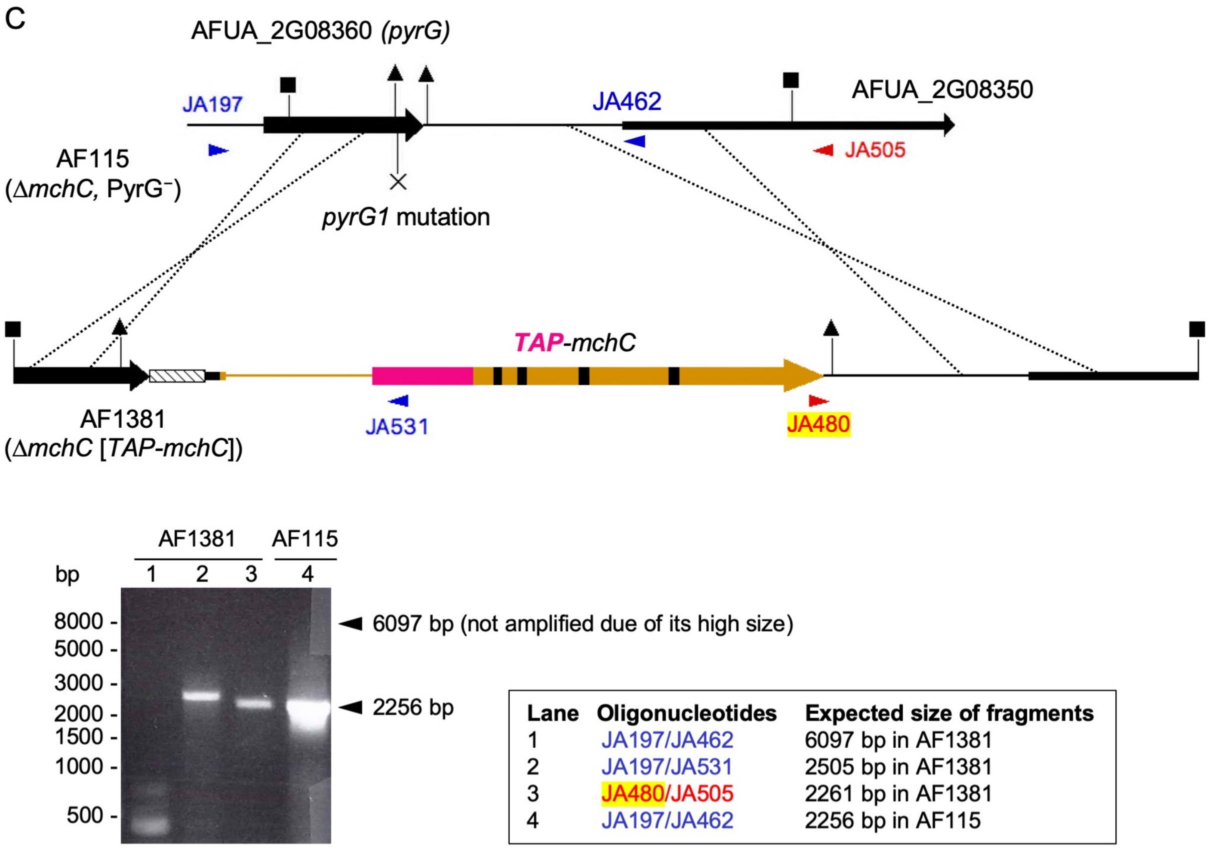

Lane	Oligonucleotides	Expected size of fragments
1	JA197/JA462	6097 bp in AF1381
2	JA197/JA531	2505 bp in AF1381
3	JA480/JA505	2261 bp in AF1381
4	JA197/JA462	2256 bp in AF115

Figure S3. Construction of the ∆*mchA*^{R} ∆*mchB*^{R} and ∆*mchC*^{R} reconstituted fungal strains of *Aspergillus fumigatus*. (A) To generate the **∆*mchA*^{R} AF1217** uridine-uracil-prototrophic PyrG^{+} strain, which expresses the *mchA* coding sequence (highlighted in green) fused to the TAP-tag (highlighted in pink) under the control of the wild-type *mchA* promoter region (in green), the AF127 uridine-uracil-auxotrophic PyrG^{-} ∆*mchA* mutant strain was transformed with a 5.87 kb DNA fragment that was excised from plasmid pMCH109R by digestion with EcoRI. **(B)** To generate the **∆*mchB*^{R} AF1251** uridine-uracil-prototrophic PyrG^{+} strain, which expressed the *mchB* coding sequence (highlighted in grey) fused to the TAP-tag (in pink) under the control of the wild-type *mchB* promoter region (in grey), the AF125 uridine-uracil-auxotrophic PyrG^{-} ∆*mchB* mutant strain was transformed with a 5.41 kb DNA fragment that was excised from plasmid pMCH209R by digestion with SpeI. **(B)** To generate the **∆*mchC*^{R} AF1381** uridine-uracil-prototrophic PyrG^{+} strain, which expressed the *mchC* coding sequence (highlighted in orange) fused to the TAP-tag (in pink) under the control of the wild-type *mchB* promoter region (in orange), the AF115 uridine-uracil-auxotrophic PyrG^{-} ∆*mchC* mutant strain was transformed with a 6.68 kb DNA fragment that was excised from plasmid pMCH3091R by digestion with EcoRI. All transformant DNA fragments were designed to be inserted between the AFUA_2G08360 (*pyrG*) and AFUA_2G08350 genes of any uridine-uracil-auxotrophic PyrG^{-} strain carrying the *pyrG1* mutation. The ∆*mchA*^{R} ∆*mchB*^{R} and ∆*mchC*^{R} reconstituted strains harboured the expected DNA fragments in their *pyrG* loci, as verified by PCR using as primers three pairs of oligonucleotides. The JA197/JA462 oligonucleotides pair amplify a DNA fragment of 2256 bp exclusively in the non-transformed parental strains.

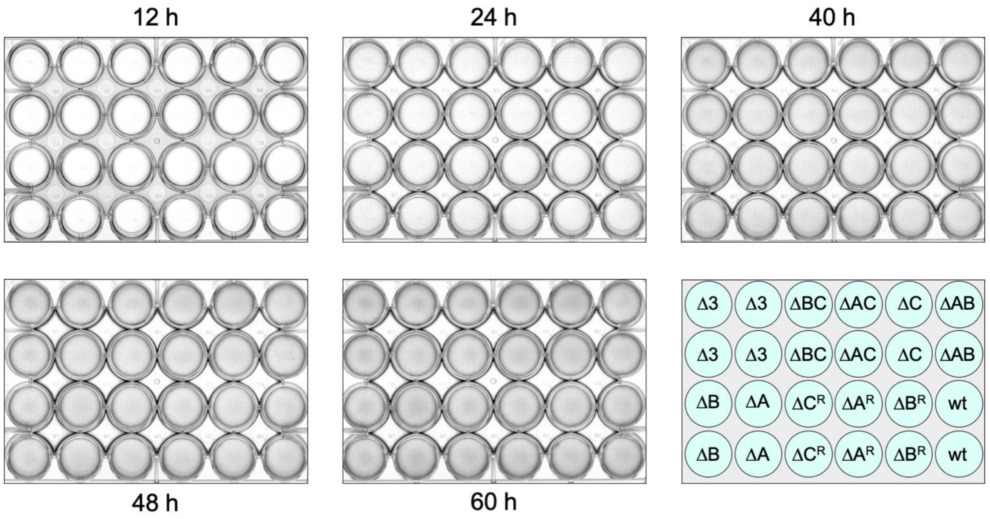

wt	wild-type	CEA10
ΔA	Δ*mchA*	AF121
ΔB	Δ*mchB*	AF117
ΔC	Δ*mchC*	AF114

ΔAB	Δ*mchA*Δ*mchB*	AF131
ΔBC	Δ*mchB*Δ*mchC*	AF119
ΔAC	Δ*mchA*Δ*mchC*	AF124
Δ3	Δ*mchA*Δ*mchB*Δ*mchC*	AF147
ΔA^R	Δ*mchA*[*TAP-mchA*]	AF1271
ΔB^R	Δ*mchB*[*TAP-mchB*]	AF1251
ΔC^R	Δ*mchC*[*TAP-mchC*]	AF1381

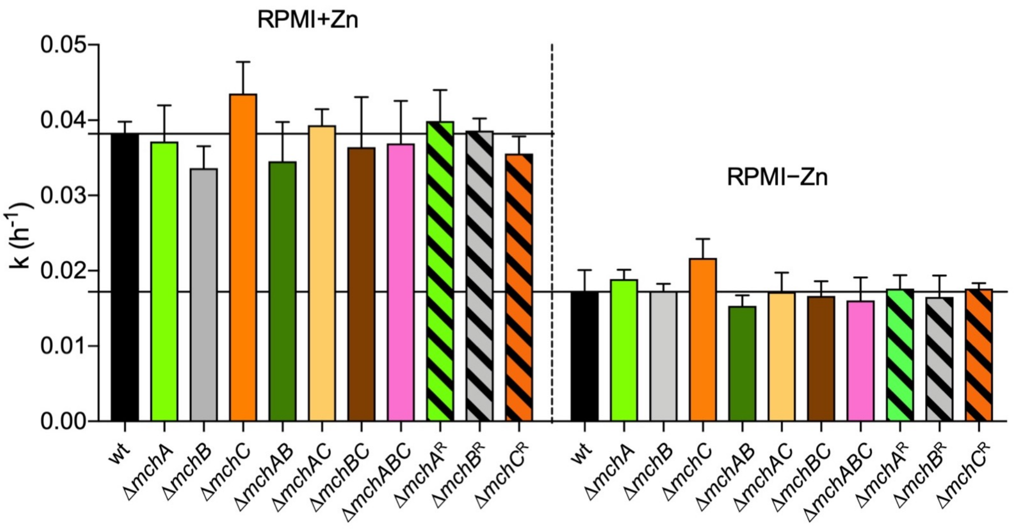

Figure S4. Growth rate of the Δ*mch* mutant strain of *Aspergillus fumigatus*. 10^5 conidia from each mutant strain were inoculated in 1 mL of the zinc-limiting RPMI-1640 complete medium (i.e., RPMI supplemented with 10 μM Fe^{2+}, 1 μM Cu^{2+}, 1 μM Mn^{2+} and 0,05% Tween 20) (RPMI–Zn) and in this medium supplemented with 10 μM Zn^{2+} (RPMI+Zn) and cultured by duplicate in a 24-well plate at 37ºC without shaking. Plates were scanned every 4 hours in the range from 12 to 48 h (in RPMI+Zn) and from 12 to 72 h (in RPMI–Zn) after inoculation. In the figure are only shown plates with cultures in RPMI–Zn. Images were processed with ImageJ/Fiji software to measure density of cultures. Growth rates (k, h^{-1}) were calculated after delimiting the exponential growth phase for each mutant strain. Results are the average of three independent experiments. Bars indicate standard deviation. Data were analyzed statistically by applying a non-paired, two-tailed T-test and taking the growth rate of the wild-type strain as a reference. For all comparisons P values were > 0.05.

A

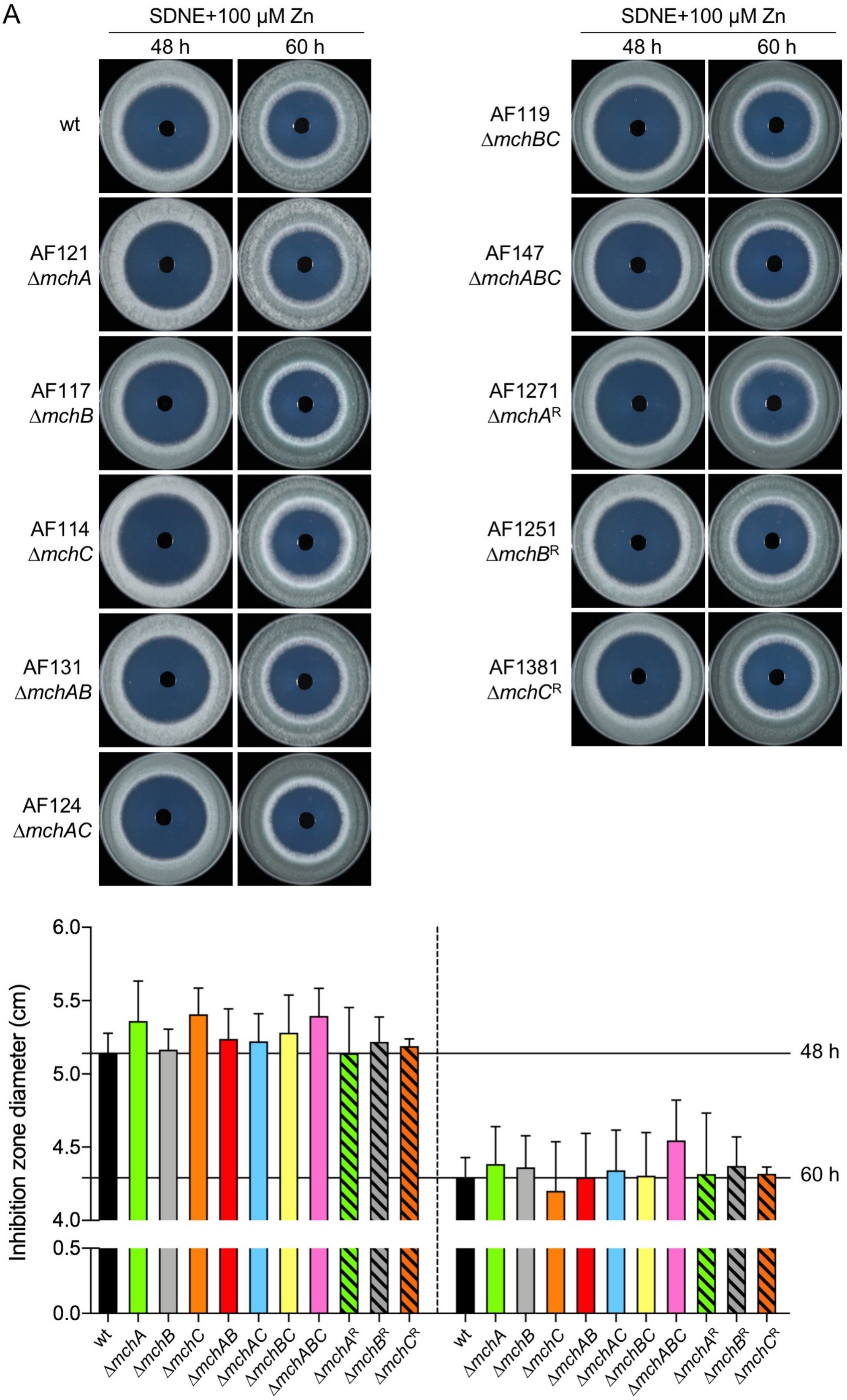
SDNE+100 µM Zn
48 h
60 h
wt
AF121 ΔmchA
AF117 ΔmchB
AF114 ΔmchC
AF131 ΔmchAB
AF124 ΔmchAC
AF119 ΔmchBC
AF147 ΔmchABC
AF1271 ΔmchA^R
AF1251 ΔmchB^R
AF1381 ΔmchC^R
Inhibition zone diameter (cm)
6.0
5.5
5.0
4.5
4.0
0.5
0.0
48 h
60 h
wt
ΔmchA
ΔmchB
ΔmchC
ΔmchAB
ΔmchAC
ΔmchBC
ΔmchABC
ΔmchA^R
ΔmchB^R
ΔmchC^R

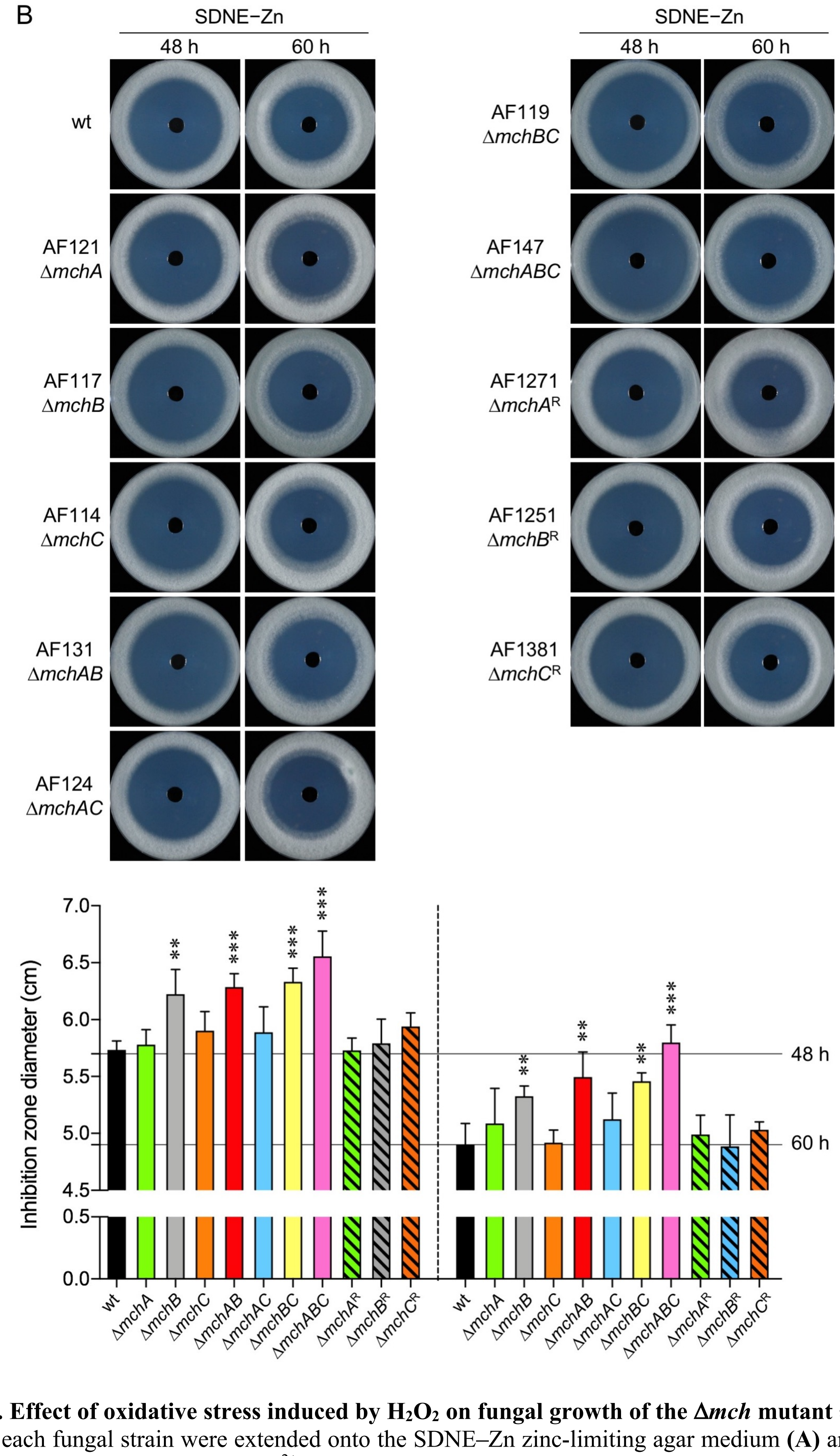

Figure S5. Effect of oxidative stress induced by H_2O_2 on fungal growth of the Δ*mch* mutant strains. 10^6 conidia of each fungal strain were extended onto the SDNE–Zn zinc-limiting agar medium **(A)** and onto this medium supplemented with 100 µM Zn^{2+} **(B)**. A well in the middle of every plate was filled with 0.3 mL of a fresh 3% H_2O_2 solution (0.98 M). Plates were incubated at 37ºC in a humid atmosphere and photographed 48 and 60 hours after inoculation. The zone inhibition diameters were measured using the ImageJ/Fiji software. Data shown in graphs are the average of three independent experiments. Bars indicated standard deviation. Data were analyzed statistically by applying a non-paired, two-tailed T-test taking the data for the wild-type strain as a reference (*P = 0,05-0,01; **P = 0,01-0,001; ***P = 0,001-0,0001; ****P < 0.0001).

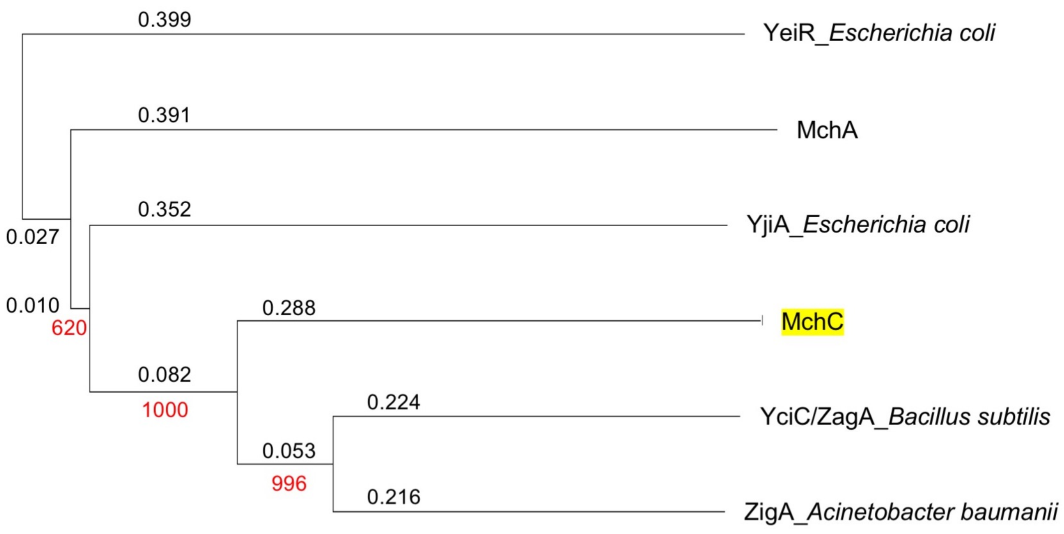

Figure S6. Phylogenetic relationship of the Mch proteins with the best characterized COG0523 proteins from bacteria involved in zinc homeostasis. Only four prokaryotic COG0523 proteins with a proven role in adaptation of bacteria to zinc deficiency have been reported to date, including YeiR and YjiA from *Escherichia coli*, ZigA from *Acinetobacter baumanii* and ZagA from *Bacillus subtilis*. ZigA and ZagA exhibit a 56% identity and 69% similarity (with a query cover of 92%). Phylogenetic tree constructed with Clustal X 2.0 (randon number generator seed set at 111 and number of bootstrap trials set at 1000). Branch length values (evolutionary distance) are in black. Number of bootstrap trials are in red.

E. coli	*S. cerevisiae*	*A. fumigatus*	Enzymatic activity
FolE	Fol2	AFUA_5G03140	GTP cyclohydrolase I (GCYH-I)
NudB	?	?	7,8-di-hydroneopterin triphosphate pyrophosphatase
FolB	Fol1	AFUA_3G10090	7,8-di-hydroneopterin aldolase (DHNA)
FolK	Fol1	AFUA_2G09840	6-hydroxymethyl dihydropteridine pyrophosphokinase (HPPK)
FolP	Fol1	AFUA_2G09840	7,8-dihydropteroate synthase (DHPS)
FolC	Fol3	AFUA_4G06740	7,8-dihydrofolate synthase
FolA	Dfr1	AFUA_2G08890	5,6,7,8-dihydrofolate reductase

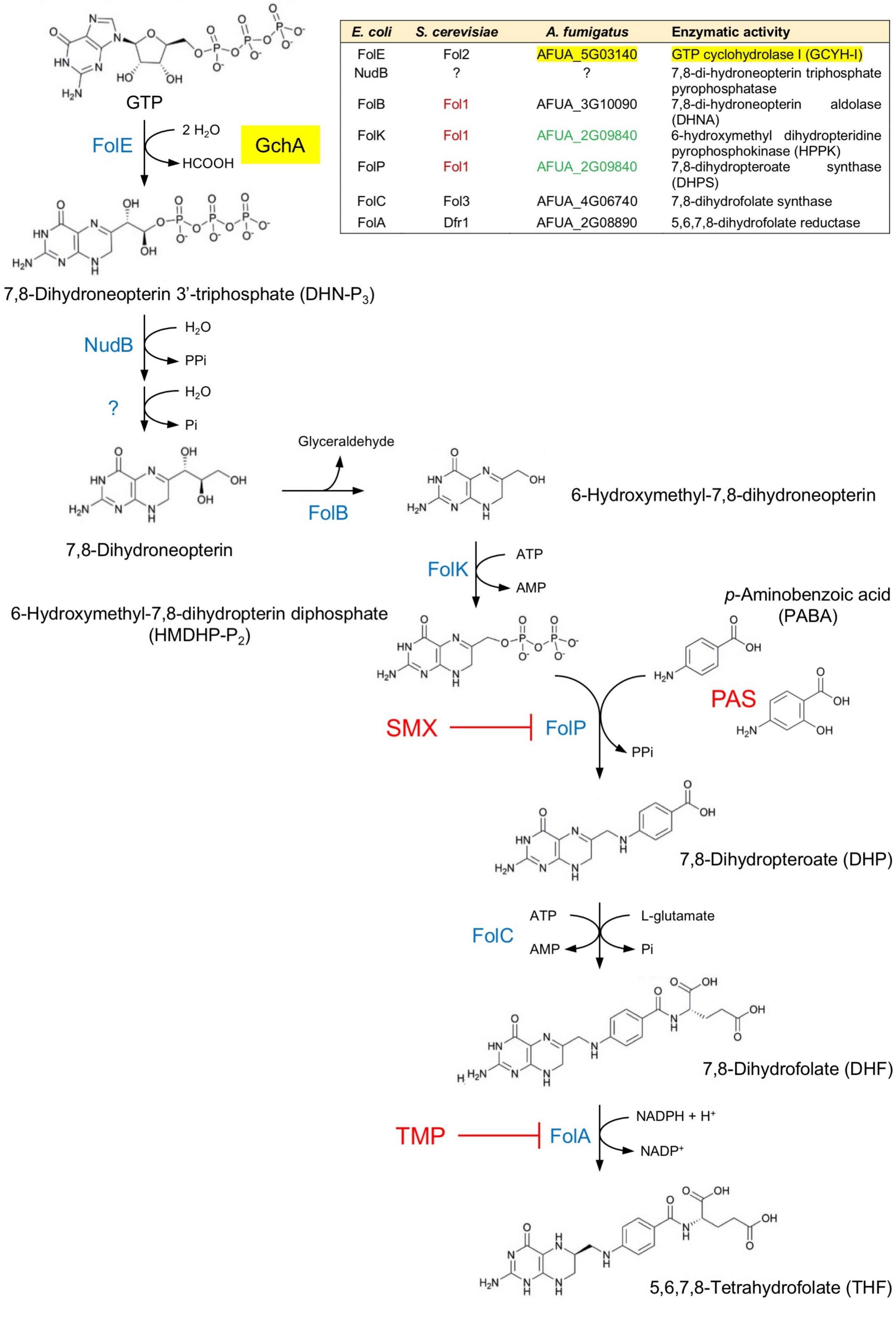

Figure S7. Tetrahydrofolate biosynthetic pathway in bacteria and fungi. Enzymes from *Escherichia coli* are indicated in blue and are used as a reference for their orthologous in *S. cerevisiae* and *A. fumigatus*. The putative target of MchC in *A. fumigatus* has been highlighted in yellow.

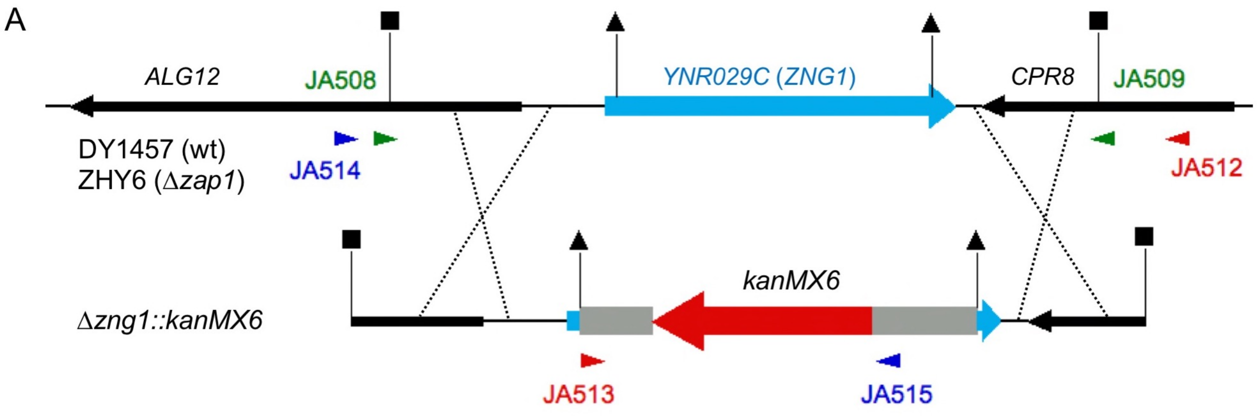

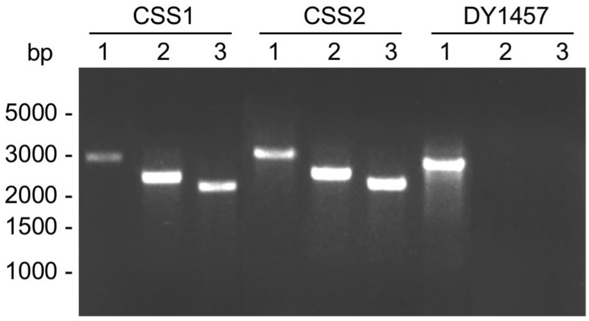

Strain	Parental	Relevant genotype
CSS1	DY1457	*ura3-52 Δzng1*
CSS2	ZHY6	*ura3-52 Δzap1Δzng1*

Lane	Oligonucleotides	Expected size of fragments
1.	JA508/JA509	2910 bp (Δ*zng1*); 2609 bp (wt)
2.	JA513/JA512	2289 bp
3.	JA514/JA515	2098 bp

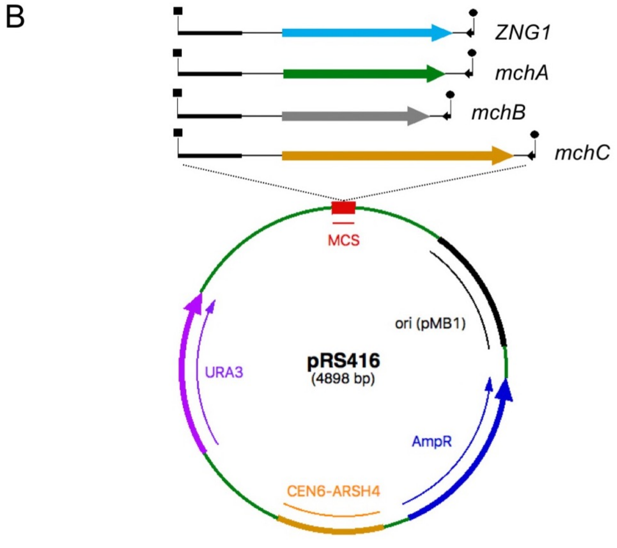

Strain	Relevant genotype
CSS10	*ura3-52 Δzng1 [URA3]*
CSS11	*ura3-52 Δzng1 [URA3, ZNG1]*
CSS12	*ura3-52 Δzng1 [URA3, mchA]*
CSS13	*ura3-52 Δzng1 [URA3, mchB]*
CSS14	*ura3-52 Δzng1 [URA3, mchC]*
CSS20	*ura3-52 Δzap1Δzng1[URA3]*
CSS21	*ura3-52 Δzap1Δzng1[URA3, ZNG1]*
CSS22	*ura3-52 Δzap1Δzng1[URA3, mchA]*
CSS23	*ura3-52 Δzap1Δzng1[URA3, mchB]*
CSS24	*ura3-52 Δzap1Δzng1[URA3, mchC]*

Figure S8. Construction of Δ*zng1* and complemented strain of *Saccharomyces cerevisiae*. (A) Nearly all coding sequence of the *YNR029C/ZNG1* gene (blue region delimited by triangles) was replaced in the yeast strains DY1457 (formally considered as a wild-type) and ZHY6 (formally considered as a Δ*zap1* mutant) by the *KanMX6* selector module upon transforming these yeast strains with a DNA fragment of 2910 bp obtained from plasmid pZCH3 to generate the Δ*zng1* (CSS1) and Δ*zng1*Δ*zap1* (CSS2) yeast strains respectively, that were verified by PCR using the pairs of oligonucleotides shown below the scheme. **(B)** To generate the complemented prototrophic Ura$^+$ Δ*zng1*[*ZNG1*], Δ*zng1*[*mchA*], Δ*zng1*[*mchB*] and Δ*zng1*[*mchC*] yeast strains, the CSS1 and CSS2 mutant strains were transformed with pRS416 derivative plasmids that carried the coding sequence of *ZNG1* (pZCH13) or the cDNA sequence of *mchA* (pMCH113), *mchB* (pMCH210) or *mchC* (pMCH321) under control of the *ZNG1* promoter region and selected on minimal medium without uracil. The CSS1 and CSS2 mutant strains were also transformed with the empty pRS416 centromeric plasmid to generate respectively the ASF0 and ASF11 prototrophic Ura$^+$ strains to be used as controls in complementation assays.

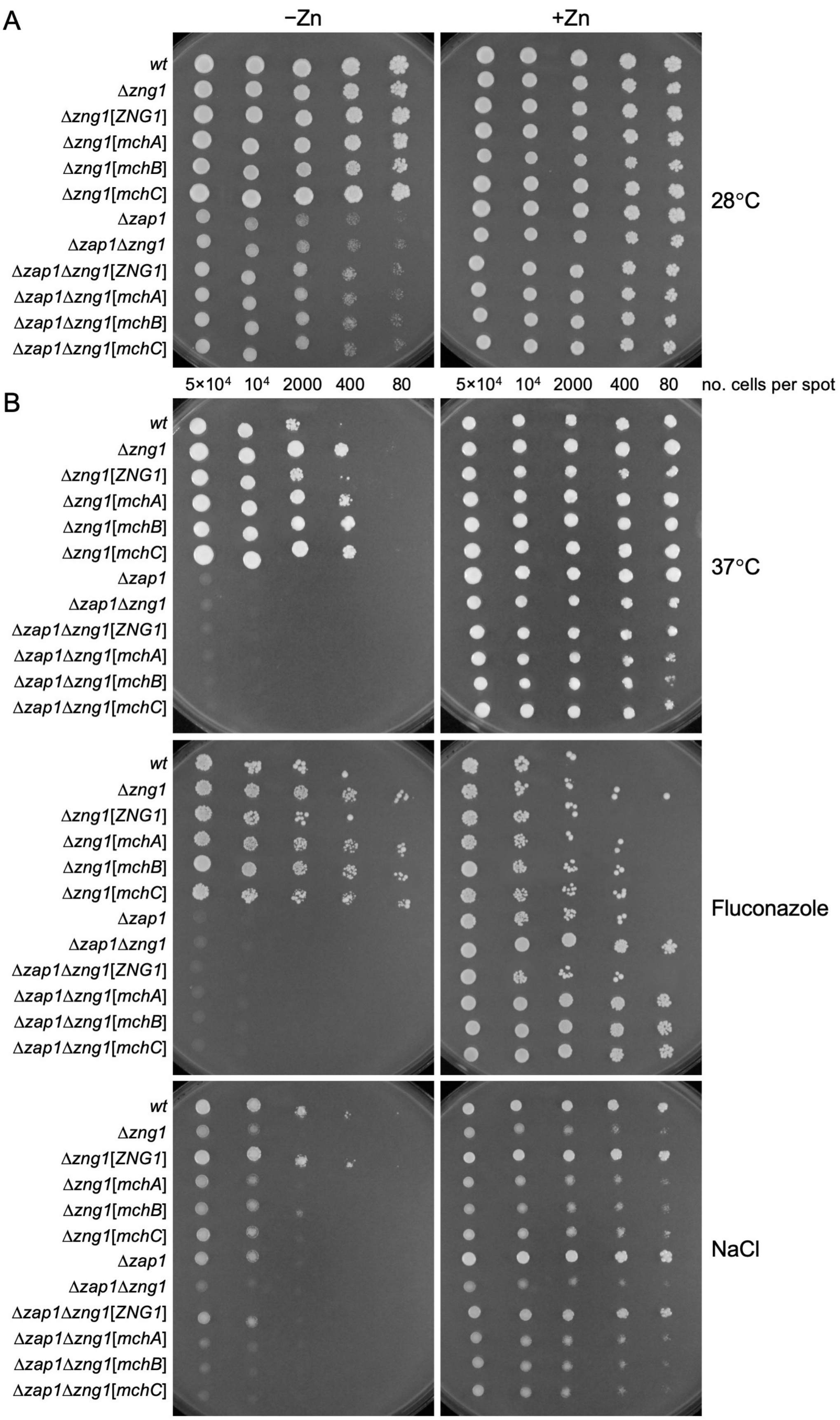

Figure S9. Functional analysis of the *mch* genes in *S. cerevisiae*. (A) The indicated number of yeast cells of the different prototrophic Ura$^+$ mutant strains were spotted onto agar plates of the SDAE+CSM–Zn–URA zinc-limiting medium (–Zn) and onto agar plates of this medium supplemented with 0.2 mM Zn^{2+} (+Zn) and incubated at 28ºC. **(B)** Yeast cells were incubated at 37ºC for growth under thermal stress and onto the same media supplemented with either fluconazole (40 µg/mL) or with NaCl (0.8 M) and incubated at 28ºC. All plates were incubated for 5 days in a humid atmosphere before pictures were taken.

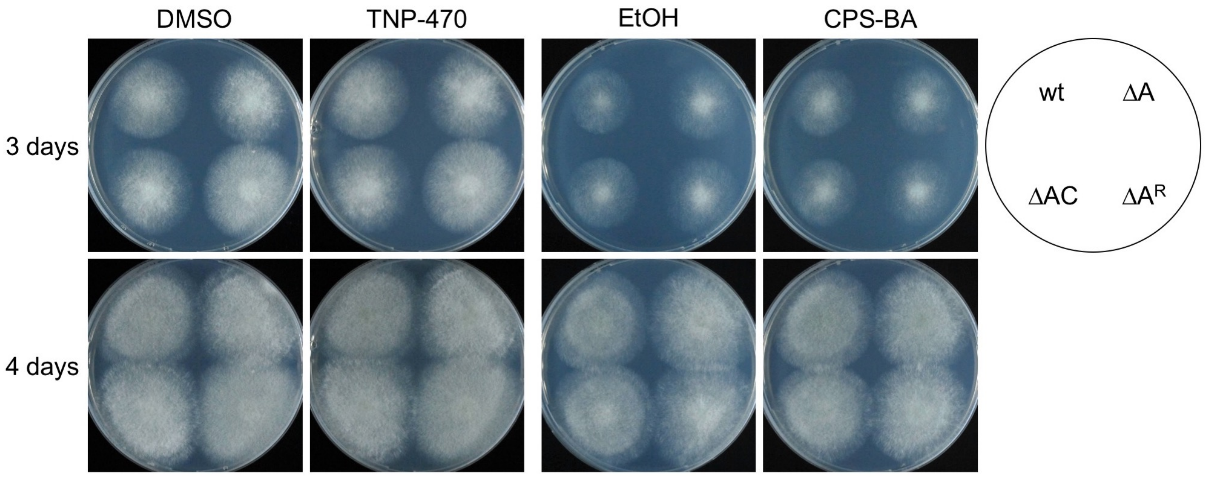

Figure S10. Effect of MetAP inhibitors on the growth ability of Δ*mchA* fungal strains. 10^3 spores of the indicated fungal strain were spotted onto agar plates of the AMME–Zn medium supplemented with 25 μM TNP-470 (dissolved in DMSO) or 320 μM CPS-BA (dissolved in ethanol). Plates were incubated at 37ºC for 3-4 days in a humid atmosphere before pictures were taken.

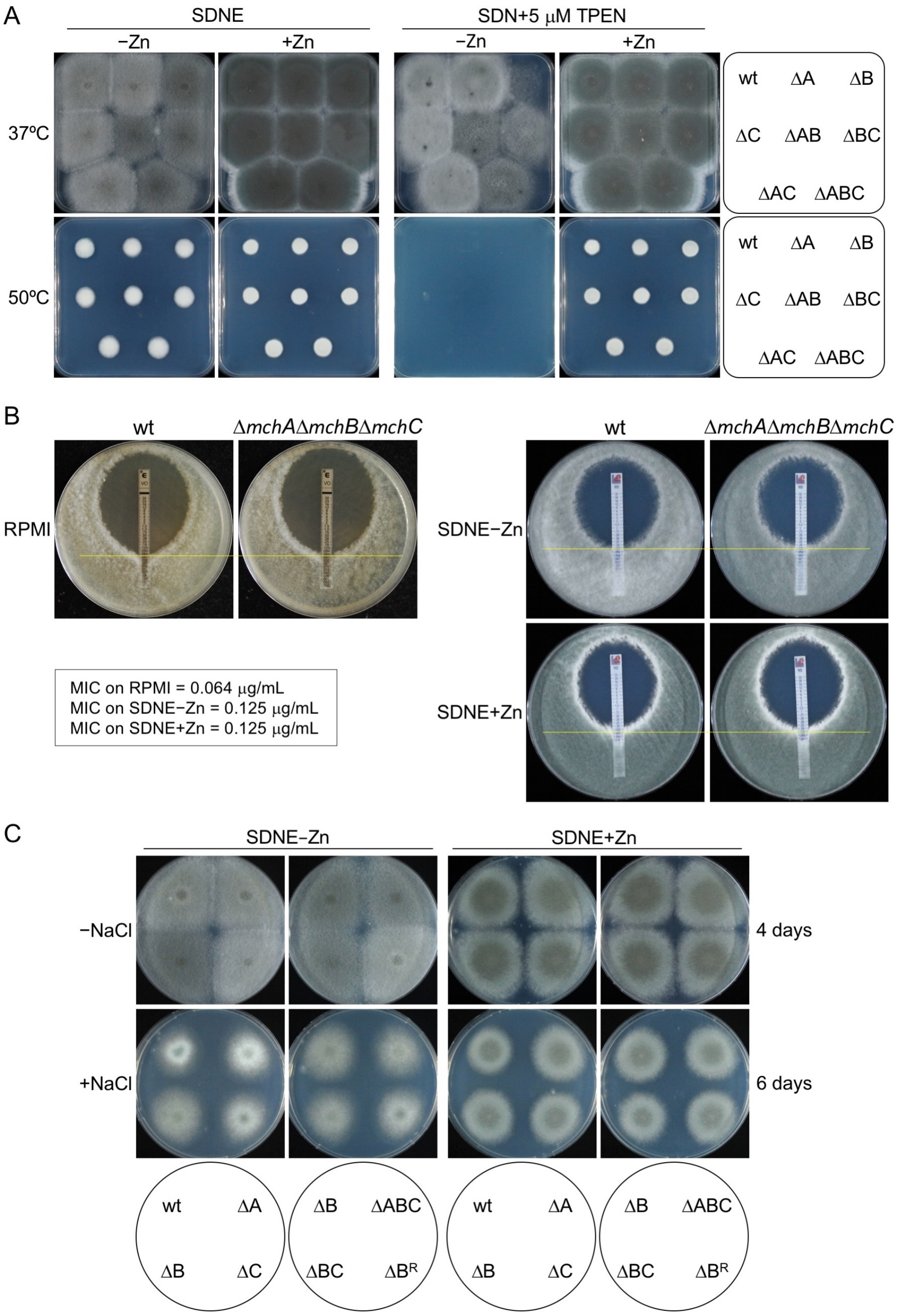

Figure S11. Effect of different stresses on fungal growth of the Δ*mch* mutant strains. (A) 10^3 conidia from the indicated mutant strains were spotted onto agar plates of the SDNE–Zn, SDN–Zn medium supplemented with 5 μM TPEN and onto these media supplemented with 100 μM Zn^{2+} (+Zn). Unlike EDTA, the specific zinc chelator TPEN is able to enter the cells growing in neutral and alkaline media. Plates were incubated at 37ºC and 50ºC for 4 days in a humid atmosphere before pictures were taken. Interestingly, sequestration of intracellular zinc with TPEN abolished fungal growth at 50ºC. **(B)** 10^6 conidia of the CEA10 (wt) and AF147 (Δ*mchA*Δ*mchB*Δ*mchC*) fungal strains were extended onto RPMI-1640 (pH 7,0) agar plates supplemented with 2% glucose. E-test strips preloaded with a voriconazole gradient (0,002-32 μg/mL; AB Bio-disk) were put on the top of the inoculated media. Similarly, these fungal strains were extended onto SDNE–Zn (pH 7,5) agar plates without a zinc supplement and supplemented with 100 μM zinc. E-test strips preloaded with a voriconazole gradient (0,002-32 μg/mL; LIOFILCHEM) were put on the top of the inoculated media. Plates were incubated at 37ºC in a humid atmosphere for 2 days before pictures were taken. **(C)** 10^3 conidia from the indicated mutant strains were spotted onto agar plates of the SDNE–Zn medium and onto this medium supplemented with 100 μM Zn^{2+}, both without NaCl or supplemented with 1.2 M NaCl, as indicated. Plates were incubated at 37ºC for 4 days (–NaCl) or 6 days (+NaCl) in a humid atmosphere before pictures were taken.
