# Supplementary material for: Zinc-metallochaperones of Aspergillus fumigatus are involved in ROS production and folate biosynthesis during zinc deficiency
Source: Microbiol Spectr. 2025 Sep 23;13(11):e02279-25. doi: 10.1128/spectrum.02279-25 (PMC12584730; doi:10.1128/spectrum.02279-25)
Supplement: Supplemental material — Supplemental text; Tables S1 to S7. [file spectrum.02279-25-s0003.pdf]

SUPPLEMENTARY TEXT

1. Distinctive structural features, distribution and phylogenetic analysis of the MchA, MchB and MchC proteins of *A. fumigatus*

The *mchA* gene spans a coding sequence of 1484 bp interrupted by five introns that encodes a putative protein of 406 amino acids (44.1 kDa). The *mchB* gene spans an intron-free coding sequence of 1110 bp that encodes a putative protein of 369 amino acids (40.5 kDa). The *mchC* gene spans a coding sequence of 1976 bp interrupted by four introns that encodes a putative protein of 585 amino acids (66.4 kDa). All sequences perfectly matched the ones annotated in FungiDB (https://fungidb.org/fungidb/app), except the coding sequence annotated for *mchC* that was 66 bp longer towards its 5'-end than the actual sequence.

The amino acid sequence downstream of the GChCC motif in the MchA- and MchC-like proteins was GChCCxx[KRH]x, whereas in MchB-like proteins was GChCCxx[VAL]G. Hence, this conserved sequence in the MchB-like proteins may constitute a signature sequence for these kind of proteins (Fig. S1A).

The C-terminal half of the Mch proteins showed an overall low degree of similarity among them. Nevertheless, we realized that in C-terminal half of all COG0523 proteins, including the Mch proteins of *A. fumigatus*, harbored a highly conserved RxK motif not reported previously (Fig. S1B). Moreover, we noticed that towards the C-terminus of the RxK motif in the MchC-like proteins was conserved the sequence motif RxKGxx[WF]x_{14}Gx_{10}[WF], which constitutes a signature sequence for the MchC-like proteins that allows to differentiate easily the MchC- from the MchA-like proteins (Fig. S1B). Besides, nearly all MchC-like proteins from fungi, green algae and protist of the SAR supergroup included in our analysis carried an acidic domain located just upstream the RxK motif (Fig. S1C).

Histidine-rich motifs in proteins have been related with their ability to bind metals (1). Most His residues in the MchA and MchC proteins from different *Aspergillus* species are scattered across their whole amino acid sequences. Nevertheless, in the MchC proteins some of their His residues are arranged alternated with small amino acids (Gly, Ala, Ser, Val) generating well-defined His-rich sequences located in their C-terminal ends (Fig. S1D). In contrast, in the MchB proteins of *Aspergillus* nearly all His residues are arranged in an alternate manner creating well-defined His-rich sequences located about 35-42 amino acids upstream of the RxK motifs (Fig. S1D).

A phylogenetic analysis of the Mch proteins of *A. fumigatus* together with other COG0523 proteins from different prokaryotic and eukaryotic organisms showed that the MchA- and MchC-like proteins represent two different branches of the same clade whereas the MchB-like proteins belongs to a separate clade (Fig. S1E). The MchA-like proteins are widely distributed from archaea and bacteria to humans. The MchB-like proteins are found scattered among fungi of subphyla Pezizomycotina (phylum *Ascomycota*), Agaromycotina (phylum *Basidiomycota*), Kickxellomycotina (phylum *Zoopagomycota*) and Mucoromycotina (phylum *Mucoromycota*), in certain archaea and bacteria and in some protists of the Opisthokonta (e.g. Filasterea and Fonticulida), Haptophyta and SAR supergroups. Finally, the MchC-like proteins are widely distributed among fungi and bacteria (mainly in α, β and γ-Proteobacteria, Firmicutes and bacteria of the PVC and FCB groups), although they are also found in archaea (mainly in halobacteria),

and scattered among green algae and protists of the Haptophyta, Discoba, Opisthokonta (e.g. Filasterea, Choanoflagellata), Amebozoa and SAR supergroups.

2. Functional investigation of the *mch* genes in the yeast *Saccharomyces cerevisiae*

It has been shown that the Zng1 metallochaperone of *S. cerevisiae* is required for proper metalation of the type-1 Zn-dependent methionine aminopeptidase (MetAP-1) of *S. cerevisiae* (Map1) during zinc deficiency (2). It has been reported that the Δ*zng1* mutant strain shows a reduction in its growth ability onto agar zinc-limiting media (2). The yeast Zng1 protein is more closely related to MchA (identity 36.7%, query cover 86%, E-value 5×10^{-81}) than to MchB (identity 32.1%, query cover 41%, E-value 3×10^{-19}) or MchC (identity 25.5%, query cover 55%, E-value 3×10^{-24}). Hence, to ascertain whether the MchA protein or any other Mch protein of *A. fumigatus* could be also involved in metalation of MetAP-1, we carry out a complementation analysis using the Δ*zng1* and Δ*zap1*Δ*zng1* yeast mutant strains (Fig. S8). In addition, since the Zap1 transcription factor regulates the homeostatic and adaptive response to zinc deficiency in *S. cerevisiae* (3, 4), it would be expected that its lack in the Δ*zap1*Δ*zng1* mutant strain will exacerbate any phenotypic trait of the Δ*zng1* mutant strain under zinc deficiency. The *ZNG1* gene and the cDNA coding sequences of the *mchA*, *mchB* or *mchC* genes, all under control of the *ZNG1* promoter, were introduced into the Δ*zng1* and Δ*zap1*Δ*zng1* mutant strains (Fig. S8; Table S1), and their growth ability was tested onto agar plates of zinc-limiting (–Zn) and zinc-replete (+Zn) media under standard culture conditions at 28ºC (Fig. S9A). In addition, we also tested their growth ability on zinc-limiting and zinc-replete media under different stressing conditions, including incubation at 37ºC (for thermal stress) and in the presence of 0.8 M NaCl (for saline stress) or 40 µg/mL fluconazole (for membrane stress) (Fig. S9B). As expected, the Δ*zap1* and Δ*zap1*Δ*zng1* strains grew respectively at a lower extent than the wild-type and Δ*zng1* strains in zinc-limiting media at 28ºC but, unexpectedly and striking contrast to results obtained by Pasquini *et al.* (2), deletion of *ZNG1* did not impair yeast growth ability neither in the wild-type nor in the Δ*zap1* mutant strains on zinc-limiting media at 28ºC (Fig. S9A). Nevertheless, the different genetic background of the yeast strains used by us (DY1457 and ZHY6) and Pasquini (BY4742) could be at the basis of this inconsistency (our yeast strains carry the Δ*ade6* mutation whereas Pasquini's strain carries the Δ*lys2* mutation). In either case, we uncover that deletion of *ZNG1* increases slightly both the thermal resistance at 37ºC on zinc-limiting media and resistance to fluconazole on both zinc-limiting and zinc-replete media, while it enhances sensitivity to salt stress on both zinc-limiting and zinc-replete media. However, the expression of none *mch* gene of *A. fumigatus* in the Δ*zng1* and Δ*zap1*Δ*zng1* yeast strains was able to complement the lack of *ZNG1* (Fig. S9B).

It was reported previously, after screening thousands of Tn3 transposon insertion mutants of the *S. cerevisiae* W303-1A strain, that disruption of the *YNR029C*/*ZNG1* gene by Tn3 reduced slightly the yeast tolerance to salt stress (0.65 M), whereas deletion of this gene in a BY4741 strain made this yeast strain extremely sensitive to salt stress (5). In addition, in another independent study based on a high-resolution yeast phenomics approach, it was also detected that mutation of *YNR029C*/*ZNG1* in a BY4741 strain causes a growth defect in the presence of 0.85 M NaCl (6). Hence, the *ZNG1* gene appears to play a role in regulating the adaptive response to salt stress in *S. cerevisiae* regardless of zinc availability. In addition,

our results also suggest that the *ZNG1* gene could be involved, under both zinc-replete and zinc-limiting conditions, in regulating resistance to azoles and, hence, in ergosterol biosynthesis (Fig. S9B). It is known that accumulation of a high amount of ergosterol becomes noxious for yeast growth under salt stressing conditions and that repression of ergosterol biosynthesis set aside for reducing ergosterol content is physiologically important for yeast to adapt and/or resist properly to salt stress (7). Therefore, the sensitivity to salt stress of the Δ*zng1* mutant strain is consistent with its resistance to fluconazole conferred by an abnormally high level of ergosterol biosynthesis.

Finally, we detected not even negative or positive genetic interactions between any *mch* gene and *ZNG1*, such that we could not get any hint about the putative function of the *mch* genes expressed in a Δ*zng1* mutant strain. Although we cannot preclude the possibility that translation of the *mch* transcripts is reduced in yeasts compared to *ZNG1* transcripts due to differences in codon usage between *S. cerevisiae* and *A. fumigatus*, this is very unlikely since expression of other *A. fumigatus* genes, such as *zrfA, zrfB, zrfC* and *zafA*, in different yeast mutant strains with the same genetic background than the Δ*zng1* and Δ*zap1*Δ*zng1* yeast strains was able to rescue yeast grow defects under zinc-limiting conditions (3, 8, 9).

3. The *mch* genes are required neither for ergosterol biosynthesis, adaptation to saline stress induced by NaCl nor fungal growth at high temperature on solid zinc-limiting or zinc-replete media

Although complementation assays in yeast failed in providing any clue about the function of the *mch* genes, it could be still possible that the *mch* genes were involved in regulating the adaptive response to thermal stress during zinc deficiency and/or the adaptive response to membrane damage or salt stress under both zinc-limiting and zinc-replete conditions, as does Zng1 in *S. cerevisiae*. To investigate this, the Δ*mch* mutant strains were cultured on both zinc-limiting and zinc-replete media and incubated at 50ºC (Fig. S11A), in presence the presence of E-test strips pre-loaded with voriconazole (Fig. S11B), and onto agar plates supplemented with 1.2 M NaCl (Fig. S11C). However, none of these stressing conditions influenced the growth ability of the Δ*mch* mutant strains regardless on zinc availability. Hence, it is unlikely that the *mch* genes are involved in regulating the adaptive response to ergosterol biosynthesis, salt and thermal stress in *A. fumigatus*.

SUPPLEMENTARY TABLES

Table S1. ***Saccharomyces cerevisiae*** **strains used in this study**

Strain	Parental strain	Genotype	Brief genotype (uracil auxotrophy)
DY1457	-	*MATα ade6 his3-11,15 leu2-3,112 trp1-1 ura3-52* (4)	wt (Ura⁻)
CSS1	DY1457	*MATα ade6 his3 leu2, trp1 ura3 Δzng1::KanMX6*	*Δzng1* (Ura⁻)
ZHY6	-	*MATα ade6 his3-11,15 leu2-3,112 trp1-1 ura3-52 Δzap1::TRP1* (4)	*Δzap1* (Ura⁻)
CSS2	ZHY6	*MATα ade6 his3 leu2 trp1 ura3 Δzap1::TRP1 Δzng1::KanMX6*	*Δzap1* (Ura⁻)
ASF0	DY1457	*MATα ade6 his3 leu2, trp1 ura3* [pRS416, *URA3*]	wt
ASF11	ZHY6	*MATα ade6 his3 leu2 trp1 ura3 Δzap1::TRP1* [pRS416, *URA3*]	*Δzap1*
CSS10	CSS1	*MATα ade6 his3 leu2, trp1 ura3 Δzng1::KanMX6* [pRS416]	*Δzng1*
CSS11	CSS1	*MATα ade6 his3 leu2, trp1 ura3 Δzng1::KanMX6* [pRS416-*ZNG1*]	*Δzng1* [*ZNG1*]
CSS12	CSS1	*MATα ade6 his3 leu2, trp1 ura3 Δzng1::KanMX6* [pRS416-*mchA*]	*Δzng1* [*mchA*]
CSS13	CSS1	*MATα ade6 his3 leu2, trp1 ura3 Δzng1* [pRS416-*mchB*]	*Δzng1* [*mchB*]
CSS14	CSS1	*MATα ade6 his3 leu2, trp1 ura3 Δzng1* [pRS416-*mchC*]	*Δzng1* [*mchC*]
CSS20	CSS2	*MATα ade6 his3 leu2 trp1 ura3 Δzap1::TRP1 Δzng1::KanMX6* [pRS416]	*Δzap1Δzng1*
CSS21	CSS2	*MATα ade6 his3 leu2 trp1 ura3 Δzap1::TRP1 Δzng1::KanMX6* [pRS416-*ZNG1*]	*Δzap1Δzng1* [*ZNG1*]
CSS22	CSS2	*MATα ade6 his3 leu2 trp1 ura3 Δzap1::TRP1 Δzng1::KanMX6* [pRS416-*mchA*]	*Δzap1Δzng1* [*mchA*]
CSS23	CSS2	*MATα ade6 his3 leu2 trp1 ura3 Δzap1::TRP1 Δzng1::KanMX6* [pRS416-*mchB*]	*Δzap1Δzng1* [*mchB*]
CSS24	CSS2	*MATα ade6 his3 leu2 trp1 ura3 Δzap1::TRP1 Δzng1::KanMX6* [pRS416-*mchC*]	*Δzap1Δzng1* [*mchC*]

Table S2. Distribution of Mch proteins in the most common fungal pathogens.

Fungal pathogen	Strain	MchA	MchB	MchC
Aspergillus fumigatus	*A1163*	+	+	+
Cryptococcus gattii	*VGII R265*	+	+	+
Sporothrix brasiliensis	*5110*	+	+	+
Sporothrix schenckii	*1099-18*	+	+	+
Coccidioides immitis	*RS*	+	+	+
Trichophyton tonsurans	*CBS 112818*	+	+	+
Trichophyton mentagrophytes	*TIMM2789*	+	+	+
Fonsecaea pedrosoi	*CBS271.37*	+	+	+
Exophiala dermatitidis	*NIH UT8656*	+	+	+
Blastomyces dermatitidis	*ER-3*	+	+	–
Trichophyton rubrum	*CBS 118892*	+	+	–
Microsporum canis	*CBS 113480*	+	+	–
Mucor lusitanicus	*CBS 277.49*	+	+	–
Rhizopus microsporus var. microsporus	*ATCC 52814*	+	+	–
Paracoccidioides brasiliensis	*Pb03*	+	+	–
Histoplasma capsulatum	*G217B*	+	+	–
Pneumocystis jirovecii	*SE8*	+	–	–
Candida albicans	*SC5314*	+	–	–
Candida dubliniensis	*CD36*	+	–	–
Candida metapsilosis	*BP57*	+	–	–
Candida parapsilosis	*CDC317*	+	–	–
Candida tropicalis	*MYA-3404*	+	–	–
Candida auris	*B8441*	+	–	+
[Candida] duobushaemulonis	*B09383*	+	–	+
[Candida] haemulonis	*B11899*	+	–	+
[Candida] pseudohaemulonis	*B12108*	+	–	+
Cryptococcus neoformans var. neoformans	*JEC21*	+	–	+
Malassezia globose	*CBS 7966*	–	–	+
Malassezia pachydermatis	*CBS 1879*	–	–	+
Malassezia restricta	*KCTC 27527*	–	–	+
Malassezia sympodialis	*ATCC 42132*	–	–	+

Table S3. Oligonucleotides used in this study

Oligonucleotide	Sequence (5' → 3')	Observation
JA73	CCTGGACCTCGCTGACCGTACGG	
JA74	CTCGAGGCATACGGTGTCTAATCCAGG	XhoI
JA90	CTAGCGATGTCGCAGAGTATGCCGG	
JA91	GTTAACGGCGGGATATAACATGAGC	
JA197	TGTCTCCATCCAGATTGGCACGC	
JA264	TCAATCTAGGAGGCCTTCACACGC	
JA265	AGTGGACACGTCCCTGATACTCGC	
JA373	CGGTCGTTTGTACGGCAGCTTC	
JA374	AGGCCTGAGAAATGTAGCAGTGACTG	StuI
JA456	TGGCGCCATGCCGGCGGATATTATCG	EheI
JA457	GACTAGTACTAAGCGGCGTGTACACTCG	SpeI
JA458	ACCCGGGATGCCCGTCCCCATAACAAT	SmaI
JA459	CTCTAGAACAATCATAAGGAGCCAGCTCCA	XbaI
JA460	AGGTACCGGTGCCGGTGCTGGCGCTGGCGGCGCC**ATG**GTTATCAA CTCTGGAACTGACT	KpnI/EheI
JA461	TACTAGTCTAGACAAATCACCTAGTTACTGCGGTT	SpeI/XbaI
JA462	GCCATTTCTAAGATGAGGAGTGTCGCAG	
JA465	GCACGTGGAAGTGATCGCATAAGAGAG	PmlI
JA466	GTTAACATCTCTAGACATGGTTTGAGTTTAAGATGAAAGCCCACTC	HpaI/XbaI
JA468	GTCTAGAAGCGGTTAACGGCGTAATAGCCAAGCG	XbaI/HpaI
JA469	GCACGTGCATATGTGCAAGGAGACTTCAC	PmlI/NdeI
JA470	CTGCAAGTCTCGTTCCATTCG	
JA471	ATCTAGACTTTGCAGACAGTGATGCCCCCGAC	XbaI
JA472	TGTTAACTATGCTAGCGGGCATTTTTCTCTTTGT	HpaI/NheI
JA474	TGCTAGCAGACAAACGTCCTAGGAGCTGGCTCCTTATGAT	NheI/PshAI/AvrII
JA475	GTTAACAACATTCCACCACCTCCTGCTG	HpaI
JA477	CGCTAGCAGTGATGAAGACGACGTCTC	NheI
JA478	GCACGTGAGTTTCTAGA**CAT**CTCGTTAAAGTCAACGGTG	PmlI/XbaI
JA480	CTCTAGACCACACGTGACCGCAGTAACTAGGTGATTTGTC	XbaI/PmlI
JA481	ACGTTAACGCTAGCTCATCCTGGACAGA	HpaI
JA482	GATGGTTGCCAGACGGGATATG	
JA493	GCGCGATACAGACCGGTTCAGAC	
JA494	CAGAACAACGTTTCGCTGCTGACCA	
JA505	GCCGTTAGGGATATCTGCAATGAGTGCGT	
JA508	TACTAGTGCTGTAGAACATGAGGTGGAAACTG	SpeI
JA509	GACCAGTGAGACGCCACTTGAAGGTGA	
JA510	TGAAGAAGAAGATGGTTAACTACCCTGCCTCG	HpaI
JA511	CGAGGCAGGGTAGTTAACCATCTTCTTCTTCA	HpaI
JA512	CGGGACTATGGTGCCCAAGACAGTAATG	
JA513	AGCAGTATAGCGACCAGCATTCACATACG	
JA514	CAGCACTGAGAGCTGACACTTCCAGTC	
JA515	CTTGCTAGGATACAGTTCTCACATCACATCCG	
JA522	TTTAAAGCGGAAGAATGGGCGCCTTGAGAAACATCAAGTT	EheI
JA523	AACTTGATGTTTCTCAAGGCGCCCATTCTTCCGCTTTAAA	EheI
JA524	GTAAAACCCTGGAATGATGGCTAGCATAATAAAGACGAGG	NheI
JA525	CCTCGTCTTTATTATGCTAGCCATCATTCCAGGGTTTTAC	NheI

Oligonucleotide	Sequence (5' → 3')	Observation
JA531	GTTACGCTGCTCCTCGTTCAGGTTAG	
JA532	CTGCAGTGGAGCTGGCTCCTTATG	
JA533	GCGTTTAAAGGCGTAATAGCCAAGCGAG	
18SrRNA-D	TGTTAAACCCTGTCGTGCTG	Used for qPCR
18SrRNA-R	GTACAAAGGGCAGGGACGTA	Used for qPCR
MCHA-D	GTAGACACCTGGCTCCAATCCGTTC	Used for qPCR
MCHA-R	CAGCTTGGATAATCTTACAGGACCCGTC	Used for qPCR
MCHB-D	GAAGCTGCAGGCCAAGTATACGGATC	Used for qPCR
MCHB-R	CTTATCCGACTTAACCCACGGCGTC	Used for qPCR
MCHC-D	AGTTGCTGGATGAGTGTCTCGTGGATG	Used for qPCR
MCHC-R	CTACAATACCAAACTCAGGCCATGCCTC	Used for qPCR

Table S4. Plasmids constructed to generate mutant strains of *Aspergillus fumigatus*

Plasmid	Description
pPYRG0	It carries a DNA fragment (1194 bp) obtained by PCR using the pair of oligonucleotides JA73/JA74 and total gDNA of *A. fumigatus* as template that contains the 3′-end coding and downstream sequence of the *pyrG* gene from a wild-type strain of *A. fumigatus* (3).
pPYRG2	It is a pUC19 derivative plasmid that carries the *hiG-pyrG-hisG* cassette flanked by the EcoRI/SmaI/XbaI and HpaI/SpeI restriction sites (9).
pPYRG4	It is a pGEM-T-easy derivative plasmid carrying the *lacI-pyrG-lacI* cassette flanked by the EcoRI/SmaI/XbaI/BstZ17I/NheI and HpaI/StuI/XhoI/SpeI restriction sites (8).
pPYRG12	It is a pGEM-T-easy derivative plasmid carrying the P*pyrG-pyrG*-P*pyrG* cassette flanked by the NdeI/SpeI/HpaI/XbaI and StuI restriction sites (10).
pHISG	It carries a BglII-XbaI fragment (986 bp) of the *hisG* gene from *Escherichia coli* (10)
pLAC	It carries a DNA fragment (843 bp) obtained by PCR using the pair of oligonucleotides JA90/JA91 and plasmid pPYRG4 as template.
pPYRG11	It carries a DNA fragment (508 bp) obtained by PCR using the pair of oligonucleotides JA373/JA374 and gDNA from *Aspergillus niger* ATCC1015 as template.
pPYRGQ31	This plasmid was designed to revert specifically the *pyrG1* mutation (C756T) in any PyrG^{-} CEA17 derivative strain and select PyrG^{+} prototrophic strains bearing any DNA fragment of interest inserted between the AFUA_2G08360 (*pyrG*) and AFUA_2G08350 coding sequences of *A. fumigatus*.
pTAP1	It is a pUC derivative plasmid that carries a SacII/NheI-EheI DNA fragment (572 bp) encoding a TAP-tag whose codon usage frequency is adapted to that of *A. fumigatus.*
pMCH101	It carries a DNA fragment (1284 bp) obtained by PCR using the pair of oligonucleotides JA456/JA457 and total cDNA of *A. fumigatus* as template. It contains the cDNA of the *mchA* coding sequence flanked by an EheI restriction site inserted just 5'-upstream of the ATG start codon and the SpeI restriction site inserted 3'-downstream of the stop codon of *mchA*.
pMCH102	It carries a DNA fragment (1547 bp) obtained by PCR using the pair of oligonucleotides JA456/JA457 and total gDNA of *A. fumigatus* as template. It contains the gDNA of the *mchA* coding sequence flanked by an EheI restriction site inserted just 5'-upstream of the ATG start codon and the SpeI restriction site inserted 3'-downstream of the stop codon of *mchA*.
pMCH103	It carries a DNA fragment (1930 bp) obtained by PCR using the pair of oligonucleotides JA465/JA466 and total gDNA of *A. fumigatus* as template. It contains the gDNA 5'-upstream of the ATG start codon of the *mchA* gene flanked by the PmlI and HpaI/XbaI restriction sites.
pMCH104	It carries a DNA fragment (2194 bp) obtained by PCR using the pair of oligonucleotides JA468/JA469 and total gDNA of *A. fumigatus* as template. It contains the gDNA 3'-downstream of the stop codon of the *mchA* gene flanked by the XbaI/HpaI and PmlI/NdeI restriction sites.
pMCH106D	It carries a SpeI-SmaI DNA fragment (3890 bp) containing the *hisG-pyrG-hisG* cassette that was obtained from plasmid pPYRG2 and that is flanked by the 5'-upstream PmlI-XbaI DNA fragment (1912 bp), which was obtained from plasmid pMCH103, and the 3'-downstream HpaI-NdeI DNA fragment (2169 bp) that was obtained from plasmid pMCH104.
pMCH107	It carries a SacII-EheI DNA fragment (577 bp) obtained from plasmid pTAP1 that was ligated to plasmid pMCH102 digested with SacII/EheI to generate the TAP-tagged *mchA* coding sequence.
pMCH108	It carries a BamHI-XbaI DNA fragment (508 bp) obtained from plasmid pMCH103 containing the *mchA* promoter sequence that was ligated to plasmid pPYRGQ31 digested with BglII/XbaI.
pMCH109R	It carries a NheI-SpeI DNA fragment (2103 bp) obtained from pMCH107 that was ligated to plasmid pMCH108 digested with XbaI to express the TAP-*mchA* coding sequence under control of the P*mchA* wild-type promoter sequence upon reintroduction in a Δ*mchA* PyrG^{-} strain. This plasmid was digested with PmlI/NdeI and used for transformation.
pMCH201	It carries a DNA fragment (1185 bp) obtained by PCR using the pair of oligonucleotides JA458/JA459 and total gDNA of *A. fumigatus* as template. It contains the gDNA of the *mchB* coding sequence flanked by a SmaI restriction site inserted just 5'-upstream of the ATG start codon and a XbaI restriction site inserted 3'-downstream of the stop codon of *mchB*.

Plasmid	Description
pMCH202	It carries a DNA fragment (2028 bp) obtained by PCR using the pair of oligonucleotides JA471/JA472 and total gDNA of *A. fumigatus* as template. It contains the gDNA 5'-upstream of the ATG start codon of the *mchB* gene flanked by the XbaI and HpaI/NheI restriction sites.
pMCH203	It carries a DNA fragment (2078 bp) obtained by PCR using the pair of oligonucleotides JA474/JA475 and total gDNA of *A. fumigatus* as template. It contains the gDNA 3'-downstream of the stop codon of the *mchB* gene flanked by the NheI/PshAI/AvrII and HpaI restriction sites.
pMCH205D	It carries a NheI-HpaI DNA fragment (3615 bp) containing the *lacI-pyrG-lacI* cassette that was obtained from plasmid pPYRG4 and that is flanked by the 5'-upstream XbaI-HpaI DNA fragment (2022 bp), which was obtained from plasmid pMCH202, and the 3'-downstream AvrII-HpaI DNA fragment (2057 bp) that was obtained from plasmid pMCH203.
pMCH206	It carries a SacII-EheI DNA fragment (577 bp) obtained from plasmid pTAP1 that was ligated to plasmid pMCH201 digested con SacII/SmaI to generate the TAP-tagged *mchB* coding sequence.
pMCH207	It carries a BstZ17I-NheI DNA fragment (393 bp) obtained from plasmid pMCH202 containing the *mchB* promoter region that was ligated to plasmid pPYRGQ31 digested with EheI/XbaI.
pMCH208	It carries a BstZ17I-NheI DNA fragment (393 bp) obtained from pMCH202 and ligated to plasmid pMCH206 digested with ZraI/NheI to put the TAP-*mchB* coding sequence under control of the P*mchB* wild-type promoter sequence.
pMCH209R	It was generated by replacing the BsrGI-SmaI DNA fragment of plasmid pMCH207 (264 bp) by the BsrGI-Eco53kI DNA fragment from pMCH208 (2042 bp) to express the TAP-*mchB* coding sequence under control of the wild-type *mchB* promoter region upon reintroduction in a Δ*mchB* $PyrG^{-}$ strain.
pMCH3011	It carries a DNA fragment (1825 bp) obtained by PCR using the pair of oligonucleotides JA460/JA461 and total cDNA of *A. fumigatus* (cultured under zinc-limiting conditions) as template. It contains the cDNA of the *mchC* coding sequence flanked by the KpnI/EheI restriction sites inserted just 5'-upstream of the ATG start codon and the SpeI/XbaI restriction sites inserted 3'-downstream of the stop codon of *mchC*.
pMCH302	It carries a DNA fragment (2043 bp) obtained by PCR using the pair of oligonucleotides JA460/JA461 and total gDNA of *A. fumigatus* as template. It contains the gDNA of the *mchC* coding sequence flanked by the KpnI/EheI restriction sites inserted just 5'-upstream of the ATG start codon and the SpeI/XbaI restriction sites inserted 3'-downstream of the stop codon of *mchC*.
pMCH303	It carries a DNA fragment (2260 bp) obtained by PCR using the pair of oligonucleotides JA477/JA478 and total gDNA of *A. fumigatus* as template. It contains the gDNA 5'-upstream of the ATG start codon of the *mchC* gene flanked by the NheI and PmlI/XbaI restriction sites.
pMCH304	It carries a DNA fragment (2137 bp) obtained by PCR using the pair of oligonucleotides JA480/JA481 and total gDNA of *A. fumigatus* as template. It contains the gDNA 3'-downstream of the stop codon of the *mchC* gene flanked by the XbaI/PmlI and HpaI restriction sites.
pMCH306D	It carries the XbaI-StuI DNA fragment (2347 bp) containing the P*pyrG-pyrG*-P*pyrG* cassette that was obtained from plasmid pPYRG12 and that is flanked by the 5'-upstream NheI-XbaI DNA fragment (2243 bp), which was obtained from plasmid pMCH303, and the 3'-downstream HpaI-PmlI DNA fragment (2119 bp) that was obtained from plasmid pMCH304.
pMCH308	It carries a MscI-XbaI DNA fragment (861 bp) obtained from plasmid pMCH303 harboring the *mchC* promoter sequence and ligated to plasmid pPYRGQ31 digested with EheI/XbaI.
pMCH3071	It carries a SacII-EheI DNA fragment (568 bp) obtained from pTAP1 and ligated to plasmid pMCH302 digested con SacII/EheI to generate a TAP-tagged *mchC* coding sequence.
pMCH3091R	It carries a NheI-XbaI DNA fragment (2567 bp) obtained from plasmid pMCH3071 and ligated to plasmid pMCH308 digested with XbaI to allow expression of the *TAP-mchC* coding sequence under control of the wild-type *mchC* promoter.

Table S5. DNA fragments used as probes for southern blot

Probe target	Pair of oligonucleotides	Size (bp)	Plasmid used as template
mchA	JA469/JA470	1578	pMCH104
mchB	JA474/JA475	2078	pMCH203
mchC	JA481/JA482	1505	pMCH304
hisG	JA493/JA494	867	pHISG
lacI	JA90/JA91	844	pLAC
P*pyrG*	JA373/JA374	508	pPYRG11

Table S6. DNA fragments used as probes for northern blot

Probe target	Pair of oligonucleotides[1]	Size (bp)	Plasmid used as template
CDS of *mchA*	JA456/JA457*	1284	pMCH101
CDS of *mchB*	JA458/JA459*	1185	pMCH201
CDS of *mchC*	JA264/JA265*	1248	pMCH3011

[1]Oligonucleotides labelled with an asterisk were used for labelling with [α-^{32}P] dCTP.

Table S7. Plasmids constructed to generate mutant strains of *Saccharomyces cerevisiae*

Plasmid	Description
pRS416	Yeast centromeric plasmid (*URA3*) (11)
pFA6a-VC-KanMX6	It carries the KanMX6 module for resistance to geneticin (12)
pZCH1	It carries a DNA fragment (2609 bp) obtained by PCR using the pair of oligonucleotides JA508/JA509 and gDNA of *S. cerevisiae* as template. It contains the coding sequence (CDS) of the *YNR029C* (*ZNG1*) gene (1290 bp) and the 5'-upstream (794 bp) and 3'-downstream (525 bp) regions.
pZCH2	It is a pZCH1 derivative plasmid. pZCH1 was mutated by site directed mutagenesis using oligonucleotides JA510/JA511 to introduce a HpaI restriction site 45 bp downstream of the ATG star codon of *ZNG1*.
pZCH3	It was generated by replacing the HpaI-BglII DNA fragment of pZCH2, which harbors nearly the entire CDS of *ZNG1*, by the EcoRV-BglII (1460 bp) DNA fragment from pFA6a-VC-KanMX6 that carries the KanMX6 module.
pZCH12	This is a pZCH1 derivative that carries the CDS of the *ZNG1* gene flanked by an EheI restriction site that was inserted by site directed mutagenesis just after the ATG start codon using the pair of oligonucleotides JA522/JA523, and a NheI site that was inserted by site directed mutagenesis just downstream of the stop codon using the pair of oligonucleotides JA524/JA525.
pZCH13	It carries a SpeI-MscI DNA fragment (2239 bp) obtained from pZCH12 and ligated to pRS416 digested with SpeI/SmaI. This DNA fragment carries the 5'-upstream promoter sequence (794 bp) of *ZNG1*, the CDS of *ZNG1* flanked by the EheI/NheI restriction sites and the 3'-downstream terminator sequence of *ZNG1*.
pMCH113	This is a pZCH13 derivative plasmid whose EheI-NheI DNA fragment carrying the CDS of *ZNG1* has been replaced by an EheI-SpeI DNA fragment (1274 bp) obtained from the pMCH101 plasmid that harbors the cDNA coding sequence of *mchA* from *A. fumigatus.*
pMCH210	This is a pZCH13 derivative plasmid whose EheI-NheI DNA fragment carrying the CDS of *ZNG1* has been replaced by a SmaI-XbaI DNA fragment (1175 bp) obtained from the pMCH201 plasmid that harbors the cDNA coding sequence of *mchB* from *A. fumigatus.*
pMCH312	This is a pZCH13 derivative plasmid whose EheI-NheI DNA fragment carrying the CDS of *ZNG1* has been replaced by an EheI-XbaI DNA fragment (1783 bp) obtained from the pMCH3011 plasmid that harbors the cDNA coding sequence of *mchC* from *A. fumigatus.*

SUPPLEMENTARY REFERENCES

1. Rowinska-Zyrek M, Witkowska D, Remelli M, Kozlowski H. 2013. His-rich sequences – is plagiarism from nature a good idea? New J Chem 37:58-70.
2. Pasquini M, Grosjean N, Hixson KK, Nicora CD, Yee EF, Lipton M, Blaby IK, Haley JD, Blaby-Haas CE. 2022. Zng1 is a GTP-dependent zinc transferase needed for activation of methionine aminopeptidase. Cell Rep 39:110834.
3. Moreno MA, Ibrahim-Granet O, Vicentefranqueira R, Amich J, Ave P, Leal F, Latgé JP, Calera JA. 2007. The regulation of zinc homeostasis by the ZafA transcriptional activator is essential for *Aspergillus fumigatus* virulence. Mol Microbiol 64:1182-97.
4. Zhao H, Eide DJ. 1997. Zap1p, a metalloregulatory protein involved in zinc-responsive transcriptional regulation in *Saccharomyces cerevisiae*. Mol Cell Biol 17:5044-52.
5. de Jesus Ferreira MC, Bao X, Laize V, Hohmann S. 2001. Transposon mutagenesis reveals novel loci affecting tolerance to salt stress and growth at low temperature. Curr Genet 40:27-39.
6. Warringer J, Ericson E, Fernández L, Nerman O, Blomberg A. 2003. High-resolution yeast phenomics resolves different physiological features in the saline response. Proc Natl Acad Sci U S A 100:15724-9.
7. Martínez-Montañés F, Pascual-Ahuir A, Proft M. 2011. Repression of ergosterol biosynthesis is essential for stress resistance and is mediated by the Hog1 MAP kinase and the Mot3 and Rox1 transcription factors. Mol Microbiol 79:1008-23.
8. Amich J, Vicentefranqueira R, Leal F, Calera JA. 2010. *Aspergillus fumigatus* survival in alkaline and extreme zinc-limiting environments relies on the induction of a zinc homeostasis system encoded by the *zrfC* and *aspf2* genes. Eukaryot Cell 9:424-37.
9. Vicentefranqueira R, Moreno MA, Leal F, Calera JA. 2005. The *zrfA* and *zrfB* genes of *Aspergillus fumigatus* encode the zinc transporter proteins of a zinc uptake system induced in an acid, zinc-depleted environment. Eukaryot Cell 4:837-48.
10. Toledo H, Sánchez CI, Marín L, Amich J, Calera JA. 2022. Regulation of zinc homeostatic genes by environmental pH in the filamentous fungus *Aspergillus fumigatus*. Environ Microbiol 24:643-666.
11. Sikorski RS, Hieter P. 1989. A system of shuttle vectors and yeast host strains designed for efficient manipulation of DNA in Saccharomyces cerevisiae. Genetics 122:19-27.
12. Bahler J, Wu JQ, Longtine MS, Shah NG, McKenzie A, 3rd, Steever AB, Wach A, Philippsen P, Pringle JR. 1998. Heterologous modules for efficient and versatile PCR-based gene targeting in *Schizosaccharomyces pombe*. Yeast 14:943-51.
